# Supplementary material for: Functional canonical RNAi in mice expressing a truncated Dicer isoform and long dsRNA
Source: EMBO Rep. 2024 May 20;25(7):9. doi: 10.1038/s44319-024-00148-z (PMC11239679; doi:10.1038/s44319-024-00148-z)
Supplement: Supplementary file 2 — Table EV2 [file 44319_2024_148_MOESM2_ESM.pdf]

Table EV2 - RNA-seq samples used in the study

stage	type	genotype	tissue	sex	strain	age	library name	GEO GSE ID	GEO library ID	manuscript	note or reference
ESC	small RNA	$Dicer^{wt/wt}$	ESC	unknown	C57Bl/6-derived RS7	-	s_ESC_WT+MosIR_RS7.1	GSE196308	GSM5870282	Fig. 3A	transfected with MosIR
ESC	small RNA	$Dicer^{wt/wt}$	ESC	unknown	C57Bl/6-derived RS7	-	s_ESC_WT+MosIR_RS7.2	GSE196308	GSM5870283	Fig. 3A	transfected with MosIR
ESC	small RNA	$Dicer^{wt/wt}$	ESC	unknown	C57Bl/6-derived RS7	-	s_ESC_WT+MosIR_RS7.3	GSE196308	GSM5870284	Fig. 3A	transfected with MosIR
ESC	small RNA	$Dicer^{\Delta HEL1/\Delta HEL1}$	ESC	unknown	C57Bl/6-derived RS7	-	s_ESC_XHOM+MosIR_RS10.1	GSE196308	GSM5870285	Fig. 3A	transfected with MosIR
ESC	small RNA	$Dicer^{\Delta HEL1/\Delta HEL1}$	ESC	unknown	C57Bl/6-derived RS7	-	s_ESC_XHOM+MosIR_RS10.2	GSE196308	GSM5870286	Fig. 3A	transfected with MosIR
ESC	small RNA	$Dicer^{\Delta HEL1/\Delta HEL1}$	ESC	unknown	C57Bl/6-derived RS7	-	s_ESC_XHOM+MosIR_RS10.3	GSE196308	GSM5870287	Fig. 3A	transfected with MosIR
ESC	small RNA	$Dicer^{\Delta HEL1/\Delta HEL1}\ Pkr^{-/-}$	ESC	unknown	C57Bl/6-derived RS7	-	s, ESC, XHOM&PKRnull+MosIR,_RSP.1	GSE196308	GSM5870288	Fig. 3A	transfected with MosIR
ESC	small RNA	$Dicer^{\Delta HEL1/\Delta HEL1}\ Pkr^{-/-}$	ESC	unknown	C57Bl/6-derived RS7	-	s_ESC_XHOM&PKRnull+MosIR_RSP.2	GSE196308	GSM5870289	Fig. 3A	transfected with MosIR
ESC	small RNA	$Dicer^{\Delta HEL1/\Delta HEL1}\ Pkr^{-/-}$	ESC	unknown	C57Bl/6-derived RS7	-	s, ESC, XHOM&PKRnull+MosIR_RSP.3	GSE196308	GSM5870290	Fig. 3A	transfected with MosIR
embryo	small RNA	$Dicer^{wt/wt}$	embryo	unknown	C57Bl/6NCrl	E15.5	s_E15.5_WT_1	GSE243016	GSM7777467	Fig. 1D	from Zapletal et al., 2022
embryo	small RNA	$Dicer^{wt/wt}$	embryo	unknown	C57Bl/6NCrl	E15.5	s_E15.5_WT_6	GSE243016	GSM7777468	Fig. 1D	from Zapletal et al., 2022
embryo	small RNA	$Dicer^{wt/wt}$	embryo	unknown	C57Bl/6NCrl	E15.5	s_E15.5_WT_8B	GSE243016	GSM7777469	Fig. 1D	from Zapletal et al., 2022
embryo	small RNA	$Dicer^{\Delta HEL1/\Delta HEL1}$	embryo	unknown	C57Bl/6NCrl	E15.5	s_E15.5_XHOM_10B_r2	GSE243016	GSM7777470	Fig. 1D	from Zapletal et al., 2022
embryo	small RNA	$Dicer^{\Delta HEL1/\Delta HEL1}$	embryo	unknown	C57Bl/6NCrl	E15.5	s_E15.5_XHOM_2	GSE243016	GSM7777471	Fig. 1D	from Zapletal et al., 2022
embryo	small RNA	$Dicer^{\Delta HEL1/\Delta HEL1}$	embryo	unknown	C57Bl/6NCrl	E15.5	s_E15.5_XHOM_3B	GSE243016	GSM7777472	Fig. 1D	from Zapletal et al., 2022
embryo	small RNA	$Dicer^{\Delta HEL1/\Delta HEL1}$	embryo	unknown	C57Bl/6NCrl	E15.5	s_E15.5_XHOM_4	GSE243016	GSM7777473	Fig. 1D	from Zapletal et al., 2022
embryo	small RNA	$Dicer^{\Delta HEL1/\Delta HEL1}$	embryo	unknown	C57Bl/6NCrl	E15.5	s_E15.5_XHOM_7B	GSE243016	GSM7777474	Fig. 1D	from Zapletal et al., 2022
embryo	small RNA	$Dicer^{\Delta HEL1/wt}$	embryo	unknown	C57Bl/6NCrl	E15.5	s-E15.5-XHET-7	GSE243016	GSM7777475	Fig. 1D	this work
embryo	small RNA	$Dicer^{\Delta HEL1/wt}$	embryo	unknown	C57Bl/6NCrl	E15.5	s-E15.5-XHET-13B	GSE243016	GSM7777476	Fig. 1D	this work
embryo	small RNA	$Dicer^{\Delta HEL1/wt}$	embryo	unknown	C57Bl/6NCrl	E15.5	s-E15.5-XHET-2B	GSE243016	GSM7777477	Fig. 1D	this work
adult	small RNA	$Dicer^{wt/wt}$	brain	male	C57Bl/6NCrl	10 weeks	brain_wt_2211_01	GSE242866	GSM7773172	Fig. 2A, EV2B, 4	this work
adult	small RNA	$Dicer^{wt/wt}$	brain	male	C57Bl/6NCrl	10 weeks	brain_wt_2211_02	GSE242866	GSM7773173	Fig. 2A, EV2B, 4	this work
adult	small RNA	$Dicer^{wt/wt}$	brain	male	C57Bl/6NCrl	10 weeks	brain_wt_2211_03	GSE242866	GSM7773174	Fig. 2A, EV2B, 4	this work
adult	small RNA	$Dicer^{SOM/wt}\ Pkr^{-/-}\ Tg(MosIR)$	brain	male	C57Bl/6NCrl	9 weeks	brain_DicerSOMhet_PKRnull_MosIR_2306_01	GSE242866	GSM7773169	Fig. 6F	this work
adult	small RNA	$Dicer^{SOM/wt}\ Pkr^{-/-}\ Tg(MosIR)$	brain	male	C57Bl/6NCrl	9 weeks	brain_DicerSOMhet_PKRnull_MosIR_2306_02	GSE242866	GSM7773170	Fig. 6F	this work
adult	small RNA	$Dicer^{SOM/wt}\ Pkr^{-/-}\ Tg(MosIR)$	brain	male	C57Bl/6NCrl	9 weeks	brain_DicerSOMhet_PKRnull_MosIR_2306_03	GSE242866	GSM7773171	Fig. 6F	this work
adult	small RNA	$Dicer^{\Delta HEL1/wt}\ Pkr^{+/-}\ Tg(MosIR)$	brain	male	C57Bl/6NCrl	11 weeks	brain_DicerDHEL1het_PKRhet_MosIR_2211_01	GSE242866	GSM7773163	Fig. 2A, EV2B, , 4, 5D, 5E	this work
adult	small RNA	$Dicer^{\Delta HEL1/wt}\ Pkr^{+/-}\ Tg(MosIR)$	brain	male	C57Bl/6NCrl	13 weeks	brain_DicerDHEL1het_PKRhet_MosIR_2211_02	GSE242866	GSM7773164	Fig. 2A, EV2B, , 4, 5D, 5E	this work
adult	small RNA	$Dicer^{\Delta HEL1/wt}\ Pkr^{+/-}\ Tg(MosIR)$	brain	male	C57Bl/6NCrl	11 weeks	brain_DicerDHEL1het_PKRhet_MosIR_2211_03	GSE242866	GSM7773165	Fig. 2A, EV2B, , 4, 5D, 5E	this work
adult	small RNA	$Dicer^{\Delta HEL1/wt}\ Pkr^{-/-}\ Tg(MosIR)$	brain	male	C57Bl/6NCrl	10 weeks	brain_DicerDHEL1het_PKRnull_MosIR_2211_01	GSE242866	GSM7773166	Fig. 6F	this work
adult	small RNA	$Dicer^{\Delta HEL1/wt}\ Pkr^{-/-}\ Tg(MosIR)$	brain	male	C57Bl/6NCrl	10 weeks	brain_DicerDHEL1het_PKRnull_MosIR_2211_02	GSE242866	GSM7773167	Fig. 6F	this work
adult	small RNA	$Dicer^{\Delta HEL1/wt}\ Pkr^{-/-}\ Tg(MosIR)$	brain	male	C57Bl/6NCrl	12 weeks	brain_DicerDHEL1het_PKRnull_MosIR_2211_03	GSE242866	GSM7773168	Fig. 6F	this work
adult	small RNA	$Dicer^{wt/wt}$	heart	male	C57Bl/6NCrl	10 weeks	heart_wt_2110_01	GSE242867	GSM7773190	Fig. 2A, 2B, EV2B, 4	this work
adult	small RNA	$Dicer^{wt/wt}$	heart	male	C57Bl/6NCrl	10 weeks	heart_wt_2110_02	GSE242867	GSM7773191	Fig. 2A, 2B, EV2B, 4	this work
adult	small RNA	$Dicer^{wt/wt}$	heart	male	C57Bl/6NCrl	10 weeks	heart_wt_2110_03	GSE242867	GSM7773192	Fig. 2A, 2B, EV2B, 4	this work
adult	small RNA	$Dicer^{SOM/wt}\ Pkr^{+/-}\ Tg(MosIR)$	heart	male	C57Bl/6NCrl	11 weeks	heart_DicerSOMhet_PKRhet_MosIR_2110_01	GSE242867	GSM7773184	Fig. 2B, 5D, 5E	this work
adult	small RNA	$Dicer^{SOM/wt}\ Pkr^{+/-}\ Tg(MosIR)$	heart	female	C57Bl/6NCrl	13 weeks	heart_DicerSOMhet_PKRhet_MosIR_2110_02	GSE242867	GSM7773185	Fig. 2B, 5D, 5E	this work
adult	small RNA	$Dicer^{SOM/wt}\ Pkr^{+/-}\ Tg(MosIR)$	heart	male	C57Bl/6NCrl	10 weeks	heart_DicerSOMhet_PKRhet_MosIR_2110_03	GSE242867	GSM7773186	Fig. 2B, 5D, 5E	this work
adult	small RNA	$Dicer^{SOM/wt}\ Pkr^{-/-}\ Tg(MosIR)$	heart	male	C57Bl/6NCrl	10 weeks	heart_DicerSOMhet_PKRnull_MosIR_2110_01	GSE242867	GSM7773187	Fig. 5E, 6F	this work
adult	small RNA	$Dicer^{SOM/wt}\ Pkr^{-/-}\ Tg(MosIR)$	heart	male	C57Bl/6NCrl	10 weeks	heart_DicerSOMhet_PKRnull_MosIR_2110_02	GSE242867	GSM7773188	Fig. 5E, 6F	this work
adult	small RNA	$Dicer^{SOM/wt}\ Pkr^{-/-}\ Tg(MosIR)$	heart	male	C57Bl/6NCrl	10 weeks	heart_DicerSOMhet_PKRnull_MosIR_2110_03	GSE242867	GSM7773189	Fig. 5E, 6F	this work
adult	small RNA	$Dicer^{\Delta HEL1/wt}\ Pkr^{+/-}\ Tg(MosIR)$	heart	male	C57Bl/6NCrl	11 weeks	heart_DicerDHEL1het_PKRhet_MosIR_2110_01	GSE242867	GSM7773178	Fig. 2A, 2B, EV2B, 4, 5D, 5E	this work
adult	small RNA	$Dicer^{\Delta HEL1/wt}\ Pkr^{+/-}\ Tg(MosIR)$	heart	male	C57Bl/6NCrl	13 weeks	heart_DicerDHEL1het_PKRhet_MosIR_2110_02	GSE242867	GSM7773179	Fig. 2A, 2B, EV2B, 4, 5D, 5E	this work
adult	small RNA	$Dicer^{\Delta HEL1/wt}\ Pkr^{+/-}\ Tg(MosIR)$	heart	male	C57Bl/6NCrl	11 weeks	heart_DicerDHEL1het_PKRhet_MosIR_2110_03	GSE242867	GSM7773180	Fig. 2A, 2B, EV2B, 4, 5D, 5E	this work
adult	small RNA	$Dicer^{\Delta HEL1/wt}\ Pkr^{-/-}\ Tg(MosIR)$	heart	male	C57Bl/6NCrl	10 weeks	heart_DicerDHEL1het_PKRnull_MosIR_2110_01	GSE242867	GSM7773181	Fig. 5E, 6F	this work
adult	small RNA	$Dicer^{\Delta HEL1/wt}\ Pkr^{-/-}\ Tg(MosIR)$	heart	male	C57Bl/6NCrl	10 weeks	heart_DicerDHEL1het_PKRnull_MosIR_2110_02	GSE242867	GSM7773182	Fig. 5E, 6F	this work
adult	small RNA	$Dicer^{\Delta HEL1/wt}\ Pkr^{-/-}\ Tg(MosIR)$	heart	male	C57Bl/6NCrl	12 weeks	heart_DicerDHEL1het_PKRnull_MosIR_2110_03	GSE242867	GSM7773183	Fig. 5E, 6F	this work
adult	small RNA	$Dicer^{wt/wt}$	liver	male	C57Bl/6NCrl	10 weeks	liver_wt_2201_01	GSE242868	GSM7773205	Fig. 2A, 2B, EV2B, 4	this work
adult	small RNA	$Dicer^{wt/wt}$	liver	male	C57Bl/6NCrl	10 weeks	liver_wt_2201_02	GSE242868	GSM7773206	Fig. 2A, 2B, EV2B, 4	this work
adult	small RNA	$Dicer^{wt/wt}$	liver	male	C57Bl/6NCrl	10 weeks	liver_wt_2201_03	GSE242868	GSM7773207	Fig. 2A, 2B, EV2B, 4	this work
adult	small RNA	$Dicer^{SOM/wt}\ Pkr^{+/-}\ Tg(MosIR)$	liver	male	C57Bl/6NCrl	11 weeks	liver_DicerSOMhet_PKRhet_MosIR_2201_01	GSE242868	GSM7773199	Fig. 2B, 5D, 5E	this work
adult	small RNA	$Dicer^{SOM/wt}\ Pkr^{+/-}\ Tg(MosIR)$	liver	female	C57Bl/6NCrl	13 weeks	liver_DicerSOMhet_PKRhet_MosIR_2201_02	GSE242868	GSM7773200	Fig. 2B, 5D, 5E	this work
adult	small RNA	$Dicer^{SOM/wt}\ Pkr^{+/-}\ Tg(MosIR)$	liver	male	C57Bl/6NCrl	10 weeks	liver_DicerSOMhet_PKRhet_MosIR_2201_03	GSE242868	GSM7773201	Fig. 2B, 5D, 5E	this work
adult	small RNA	$Dicer^{SOM/wt}\ Pkr^{-/-}\ Tg(MosIR)$	liver	male	C57Bl/6NCrl	10 weeks	liver_DicerSOMhet_PKRnull_MosIR_2201_01	GSE242868	GSM7773202	Fig. 5E, 6F	this work
adult	small RNA	$Dicer^{SOM/wt}\ Pkr^{-/-}\ Tg(MosIR)$	liver	male	C57Bl/6NCrl	10 weeks	liver_DicerSOMhet_PKRnull_MosIR_2201_02	GSE242868	GSM7773203	Fig. 5E, 6F	this work

adult	small RNA	*Dicer*$^{SOM/wt}$ *Pkr*$^{-/-}$ *Tg(MosIR)*	liver	male	C57Bl/6NCrl	10 weeks	liver_DicerSOMhet_PKRnull_MosIR_2201_03	GSE242868	GSM7773204	Fig. 5E, 6F	this work
adult	small RNA	*Dicer*$^{\Delta HEL1/wt}$ *Pkr*$^{+/-}$ *Tg(MosIR)*	liver	male	C57Bl/6NCrl	11 weeks	liver_DicerDHEL1het_PKRhet_MosIR_2201_01	GSE242868	GSM7773193	Fig. 2A, 2B, EV2B, 4, 5D, 5E	this work
adult	small RNA	*Dicer*$^{\Delta HEL1/wt}$ *Pkr*$^{+/-}$ *Tg(MosIR)*	liver	male	C57Bl/6NCrl	13 weeks	liver_DicerDHEL1het_PKRhet_MosIR_2201_02	GSE242868	GSM7773194	Fig. 2A, 2B, EV2B, 4, 5D, 5E	this work
adult	small RNA	*Dicer*$^{\Delta HEL1/wt}$ *Pkr*$^{+/-}$ *Tg(MosIR)*	liver	male	C57Bl/6NCrl	11 weeks	liver_DicerDHEL1het_PKRhet_MosIR_2201_03	GSE242868	GSM7773195	Fig. 2A, 2B, EV2B, 4, 5D, 5E	this work
adult	small RNA	*Dicer*$^{\Delta HEL1/wt}$ *Pkr*$^{-/-}$ *Tg(MosIR)*	liver	male	C57Bl/6NCrl	10 weeks	liver_DicerDHEL1het_PKRnull_MosIR_2201_01	GSE242868	GSM7773196	Fig. 5E, 6F	this work
adult	small RNA	*Dicer*$^{\Delta HEL1/wt}$ *Pkr*$^{-/-}$ *Tg(MosIR)*	liver	male	C57Bl/6NCrl	10 weeks	liver_DicerDHEL1het_PKRnull_MosIR_2201_02	GSE242868	GSM7773197	Fig. 5E, 6F	this work
adult	small RNA	*Dicer*$^{\Delta HEL1/wt}$ *Pkr*$^{-/-}$ *Tg(MosIR)*	liver	male	C57Bl/6NCrl	12 weeks	liver_DicerDHEL1het_PKRnull_MosIR_2201_03	GSE242868	GSM7773198	Fig. 5E, 6F	this work
adult	small RNA	*Dicer*$^{wt/wt}$	muscle	male	C57Bl/6NCrl	11 weeks	skeletal_muscle_wt_r1	GSE259395	GSM8115480	Fig. 4	this work
adult	small RNA	*Dicer*$^{wt/wt}$	muscle	male	C57Bl/6NCrl	10 weeks	skeletal_muscle_wt_r2	GSE259395	GSM8115481	Fig. 4	this work
adult	small RNA	*Dicer*$^{wt/wt}$	muscle	male	C57Bl/6NCrl	11 weeks	skeletal_muscle_wt_r3	GSE259395	GSM8115482	Fig. 4	this work
adult	small RNA	*Dicer*$^{SOM/wt}$ *Pkr*$^{-/-}$ *Tg(MosIR)*	muscle	male	C57Bl/6NCrl	9 weeks	skeletal_muscle_DicerSOMhet_PKRnull_MosIR_r1	GSE259395	GSM8115477	Fig. 6F	this work
adult	small RNA	*Dicer*$^{SOM/wt}$ *Pkr*$^{-/-}$ *Tg(MosIR)*	muscle	male	C57Bl/6NCrl	9 weeks	skeletal_muscle_DicerSOMhet_PKRnull_MosIR_r2	GSE259395	GSM8115478	Fig. 6F	this work
adult	small RNA	*Dicer*$^{SOM/wt}$ *Pkr*$^{-/-}$ *Tg(MosIR)*	muscle	male	C57Bl/6NCrl	9 weeks	skeletal_muscle_DicerSOMhet_PKRnull_MosIR_r3	GSE259395	GSM8115479	Fig. 6F	this work
adult	small RNA	*Dicer*$^{\Delta HEL1/wt}$ *Pkr*$^{-/-}$ *Tg(MosIR)*	muscle	male	C57Bl/6NCrl	9 weeks	skeletal_muscle_DicerDHEL1het_PKRnull_MosIR_r1	GSE259395	GSM8115474	Fig. 6F, 4	this work
adult	small RNA	*Dicer*$^{\Delta HEL1/wt}$ *Pkr*$^{-/-}$ *Tg(MosIR)*	muscle	male	C57Bl/6NCrl	11 weeks	skeletal_muscle_DicerDHEL1het_PKRnull_MosIR_r2	GSE259395	GSM8115475	Fig. 6F, 4	this work
adult	small RNA	*Dicer*$^{\Delta HEL1/wt}$ *Pkr*$^{-/-}$ *Tg(MosIR)*	muscle	male	C57Bl/6NCrl	11 weeks	skeletal_muscle_DicerDHEL1het_PKRnull_MosIR_r3	GSE259395	GSM8115476	Fig. 6F, 4	this work
adult	small RNA	*Dicer*$^{wt/wt}$	spleen	male	C57Bl/6NCrl	10 weeks	spleen_wt_2211_01	GSE242869	GSM7773229	Fig. 2A, 2B, EV2B, 4	this work
adult	small RNA	*Dicer*$^{wt/wt}$	spleen	male	C57Bl/6NCrl	10 weeks	spleen_wt_2211_02	GSE242869	GSM7773230	Fig. 2A, 2B, EV2B, 4	this work
adult	small RNA	*Dicer*$^{wt/wt}$	spleen	male	C57Bl/6NCrl	10 weeks	spleen_wt_2211_03	GSE242869	GSM7773231	Fig. 2A, 2B, EV2B, 4	this work
adult	small RNA	*Dicer*$^{SOM/wt}$ *Pkr*$^{+/-}$ *Tg(MosIR)*	spleen	female	C57Bl/6NCrl	17 weeks	spleen_DicerSOMhet_PKRhet_MosIR_2101_01	GSE242869	GSM7773217	Fig. 2B, 5D, 5E	this work
adult	small RNA	*Dicer*$^{SOM/wt}$ *Pkr*$^{+/-}$ *Tg(MosIR)*	spleen	female	C57Bl/6NCrl	11 weeks	spleen_DicerSOMhet_PKRhet_MosIR_2101_02	GSE242869	GSM7773218	Fig. 2B, 5D, 5E	this work
adult	small RNA	*Dicer*$^{SOM/wt}$ *Pkr*$^{+/-}$ *Tg(MosIR)*	spleen	male	C57Bl/6NCrl	10 weeks	spleen_DicerSOMhet_PKRhet_MosIR_2101_03	GSE242869	GSM7773219	Fig. 2B, 5D, 5E	this work
adult	small RNA	*Dicer*$^{SOM/wt}$ *Pkr*$^{-/-}$ *Tg(MosIR)*	spleen	male	C57Bl/6NCrl	17 weeks	spleen_DicerSOMhet_PKRnull_MosIR_2101_01	GSE242869	GSM7773220	Fig. 5E, 6F	this work
adult	small RNA	*Dicer*$^{SOM/wt}$ *Pkr*$^{-/-}$ *Tg(MosIR)*	spleen	female	C57Bl/6NCrl	17 weeks	spleen_DicerSOMhet_PKRnull_MosIR_2101_02	GSE242869	GSM7773221	Fig. 5E, 6F	this work
adult	small RNA	*Dicer*$^{SOM/wt}$ *Pkr*$^{-/-}$ *Tg(MosIR)*	spleen	female	C57Bl/6NCrl	8 weeks	spleen_DicerSOMhet_PKRnull_MosIR_2101_03	GSE242869	GSM7773222	Fig. 5E, 6F	this work
adult	small RNA	*Dicer*$^{\Delta HEL1/wt}$ *Pkr*$^{+/-}$ *Tg(MosIR)*	spleen	male	C57Bl/6NCrl	11 weeks	spleen_DicerDHEL1het_PKRhet_MosIR_2211_01	GSE242869	GSM7773208	Fig. 2A , 2B, EV2B, 4, 5D, 5E	this work
adult	small RNA	*Dicer*$^{\Delta HEL1/wt}$ *Pkr*$^{+/-}$ *Tg(MosIR)*	spleen	male	C57Bl/6NCrl	13 weeks	spleen_DicerDHEL1het_PKRhet_MosIR_2211_02	GSE242869	GSM7773209	Fig. 2A , 2B, EV2B, 4, 5D, 5E	this work
adult	small RNA	*Dicer*$^{\Delta HEL1/wt}$ *Pkr*$^{+/-}$ *Tg(MosIR)*	spleen	male	C57Bl/6NCrl	11 weeks	spleen_DicerDHEL1het_PKRhet_MosIR_2211_03	GSE242869	GSM7773210	Fig. 2A , 2B, EV2B, 4, 5D, 5E	this work
adult	small RNA	*Dicer*$^{\Delta HEL1/wt}$ *Pkr*$^{-/-}$ *Tg(MosIR)*	spleen	male	C57Bl/6NCrl	11 weeks	spleen_DicerDHEL1het_PKRnull_MosIR_2101_01	GSE242869	GSM7773211	Fig. 5E, 6F	this work
adult	small RNA	*Dicer*$^{\Delta HEL1/wt}$ *Pkr*$^{-/-}$ *Tg(MosIR)*	spleen	male	C57Bl/6NCrl	11 weeks	spleen_DicerDHEL1het_PKRnull_MosIR_2101_02	GSE242869	GSM7773212	Fig. 5E, 6F	this work
adult	small RNA	*Dicer*$^{\Delta HEL1/wt}$ *Pkr*$^{-/-}$ *Tg(MosIR)*	spleen	male	C57Bl/6NCrl	10 weeks	spleen_DicerDHEL1het_PKRnull_MosIR_2101_03	GSE242869	GSM7773213	Fig. 5E, 6F	this work
adult	small RNA	*Dicer*$^{wt/wt}$	thymus	male	C57Bl/6NCrl	10 weeks	thymus_wt_2211_01	GSE242870	GSM7773241	Fig. 2A, EV2B, 4	this work
adult	small RNA	*Dicer*$^{wt/wt}$	thymus	male	C57Bl/6NCrl	10 weeks	thymus_wt_2211_02	GSE242870	GSM7773242	Fig. 2A, EV2B, 4	this work
adult	small RNA	*Dicer*$^{wt/wt}$	thymus	male	C57Bl/6NCrl	10 weeks	thymus_wt_2211_03	GSE242870	GSM7773243	Fig. 2A, EV2B, 4	this work
adult	small RNA	*Dicer*$^{SOM/wt}$ *Pkr*$^{-/-}$ *Tg(MosIR)*	thymus	male	C57Bl/6NCrl	9 weeks	thymus_DicerSOMhet_PKRnull_MosIR_2306_01	GSE242870	GSM7773238	Fig. 6F	this work
adult	small RNA	*Dicer*$^{SOM/wt}$ *Pkr*$^{-/-}$ *Tg(MosIR)*	thymus	male	C57Bl/6NCrl	9 weeks	thymus_DicerSOMhet_PKRnull_MosIR_2306_02	GSE242870	GSM7773239	Fig. 6F	this work
adult	small RNA	*Dicer*$^{SOM/wt}$ *Pkr*$^{-/-}$ *Tg(MosIR)*	thymus	male	C57Bl/6NCrl	9 weeks	thymus_DicerSOMhet_PKRnull_MosIR_2306_03	GSE242870	GSM7773240	Fig. 6F	this work
adult	small RNA	*Dicer*$^{\Delta HEL1/\Delta wt}$ *Pkr*$^{+/-}$ *Tg(MosIR)*	thymus	male	C57Bl/6NCrl	11 weeks	thymus_DicerDHEL1het_PKRhet_MosIR_2211_01	GSE242870	GSM7773232	Fig. 2A, EV2B, 4, 5D, 5E	this work
adult	small RNA	*Dicer*$^{\Delta HEL1/\Delta wt}$ *Pkr*$^{+/-}$ *Tg(MosIR)*	thymus	male	C57Bl/6NCrl	13 weeks	thymus_DicerDHEL1het_PKRhet_MosIR_2211_02	GSE242870	GSM7773233	Fig. 2A, EV2B, 4, 5D, 5E	this work
adult	small RNA	*Dicer*$^{\Delta HEL1/\Delta wt}$ *Pkr*$^{+/-}$ *Tg(MosIR)*	thymus	male	C57Bl/6NCrl	11 weeks	thymus_DicerDHEL1het_PKRhet_MosIR_2211_03	GSE242870	GSM7773234	Fig. 2A, EV2B, 4, 5D, 5E	this work
adult	small RNA	*Dicer*$^{\Delta HEL1/wt}$ *Pkr*$^{-/-}$ *Tg(MosIR)*	thymus	male	C57Bl/6NCrl	10 weeks	thymus_DicerDHEL1het_PKRnull_MosIR_2211_01	GSE242870	GSM7773235	Fig. 6F	this work
adult	small RNA	*Dicer*$^{\Delta HEL1/wt}$ *Pkr*$^{-/-}$ *Tg(MosIR)*	thymus	male	C57Bl/6NCrl	10 weeks	thymus_DicerDHEL1het_PKRnull_MosIR_2211_02	GSE242870	GSM7773236	Fig. 6F	this work
adult	small RNA	*Dicer*$^{\Delta HEL1/wt}$ *Pkr*$^{-/-}$ *Tg(MosIR)*	thymus	male	C57Bl/6NCrl	12 weeks	thymus_DicerDHEL1het_PKRnull_MosIR_2211_03	GSE242870	GSM7773237	Fig. 6F	this work
adult	small RNA	*Dicer*$^{wt/wt}$	brain		C57BL/6J	3 months		GSE119661	GSM3380483-GSM3380496	Fig. 3C, EV3A	from Isakova et al., 2020
adult	small RNA	*Dicer*$^{wt/wt}$	heart		C57BL/6J	3 months		GSE119661	GSM3380497-GSM3380509	Fig. 3C, EV3A	from Isakova et al., 2020
adult	small RNA	*Dicer*$^{wt/wt}$	intestine		C57BL/6J	3 months		GSE119661	GSM3380510-GSM3380521	Fig. 3C, EV3A	from Isakova et al., 2020
adult	small RNA	*Dicer*$^{wt/wt}$	kidney		C57BL/6J	3 months		GSE119661	GSM3380522-GSM3380527	Fig. 3C, EV3A	from Isakova et al., 2020
adult	small RNA	*Dicer*$^{wt/wt}$	liver		C57BL/6J	3 months		GSE119661	GSM3380400-GSM3380413	Fig. 3C, EV3A	from Isakova et al., 2020
adult	small RNA	*Dicer*$^{wt/wt}$	liver		C57BL/6J	3 months		GSE119661	GSM3380427-GSM3380440	Fig. 3C, EV3A	from Isakova et al., 2020
adult	long RNA	*Dicer*$^{wt/wt}$	brain		C57BL/6J	7–8 weeks		PRJEB22693	SAMEA104310429-SAMEA104310431	Fig. 3C, EV3A, B	from Sollner et al., 2017
adult	long RNA	*Dicer*$^{wt/wt}$	heart		C57BL/6J	7–8 weeks		PRJEB22693	SAMEA104310441, SAMEA104310442	Fig. 3C, EV3A, B	from Sollner et al., 2017
adult	long RNA	*Dicer*$^{wt/wt}$	ileum		C57BL/6J	7–8 weeks		PRJEB22693	SAMEA104310443-SAMEA104310445	Fig. 3C, EV3A, B	from Sollner et al., 2017
adult	long RNA	*Dicer*$^{wt/wt}$	kidney		C57BL/6J	7–8 weeks		PRJEB22693	SAMEA104310449-SAMEA104310451	Fig. 3C, EV3A, B	from Sollner et al., 2017
adult	long RNA	*Dicer*$^{wt/wt}$	liver		C57BL/6J	7–8 weeks		PRJEB22693	SAMEA104310452-SAMEA104310454	Fig. 3C, EV3A, B	from Sollner et al., 2017
adult	long RNA	*Dicer*$^{wt/wt}$	muscle		C57BL/6J	7–8 weeks		PRJEB22693	SAMEA104310458-SAMEA104310460	Fig. 3C, EV3A, B	from Sollner et al., 2017
