# Supplementary figures and images for: Functional canonical RNAi in mice expressing a truncated Dicer isoform and long dsRNA
Source: EMBO Rep. 2024 May 20;25(7):9. doi: 10.1038/s44319-024-00148-z (PMC11239679; doi:10.1038/s44319-024-00148-z)

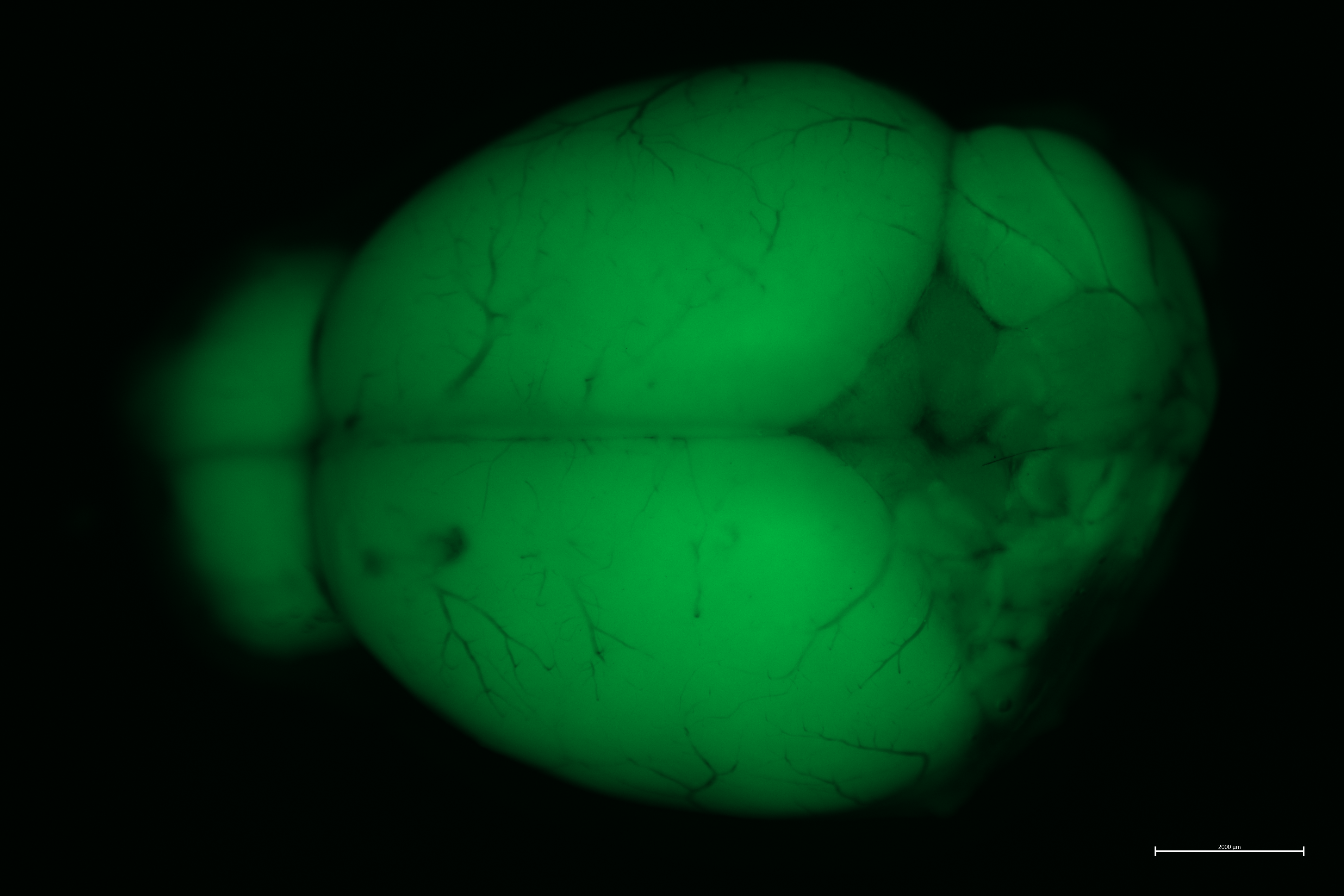

Supplement: Supplementary file 5 — Source data Fig. 5 [file 44319_2024_148_MOESM5_ESM.zip › Figure 5/Figure 5B/Brain_MosIR_400ms_EGFP.tif]

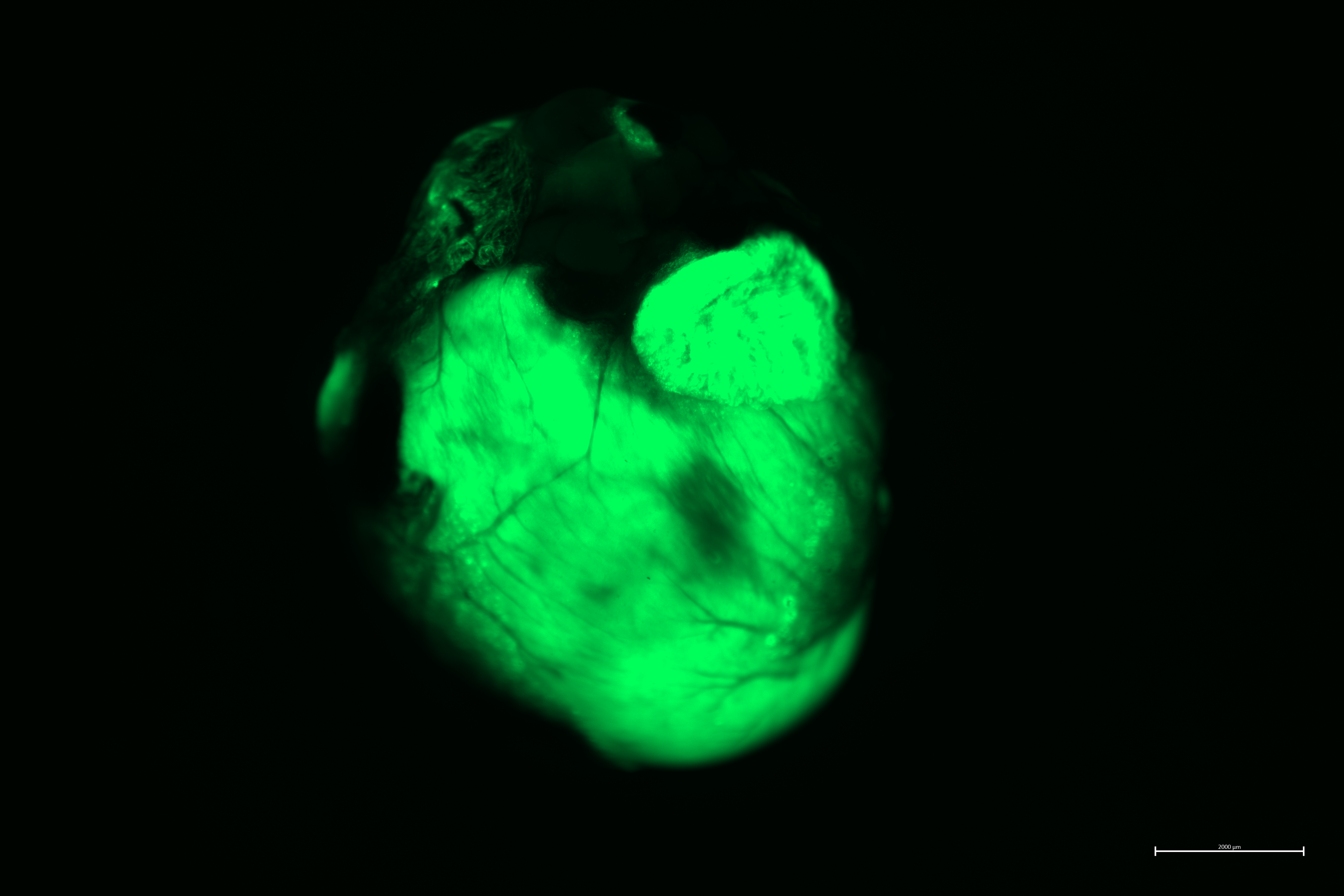

Supplement: Supplementary file 5 — Source data Fig. 5 [file 44319_2024_148_MOESM5_ESM.zip › Figure 5/Figure 5B/Heart_MosIR_400ms_EGFP.tif]

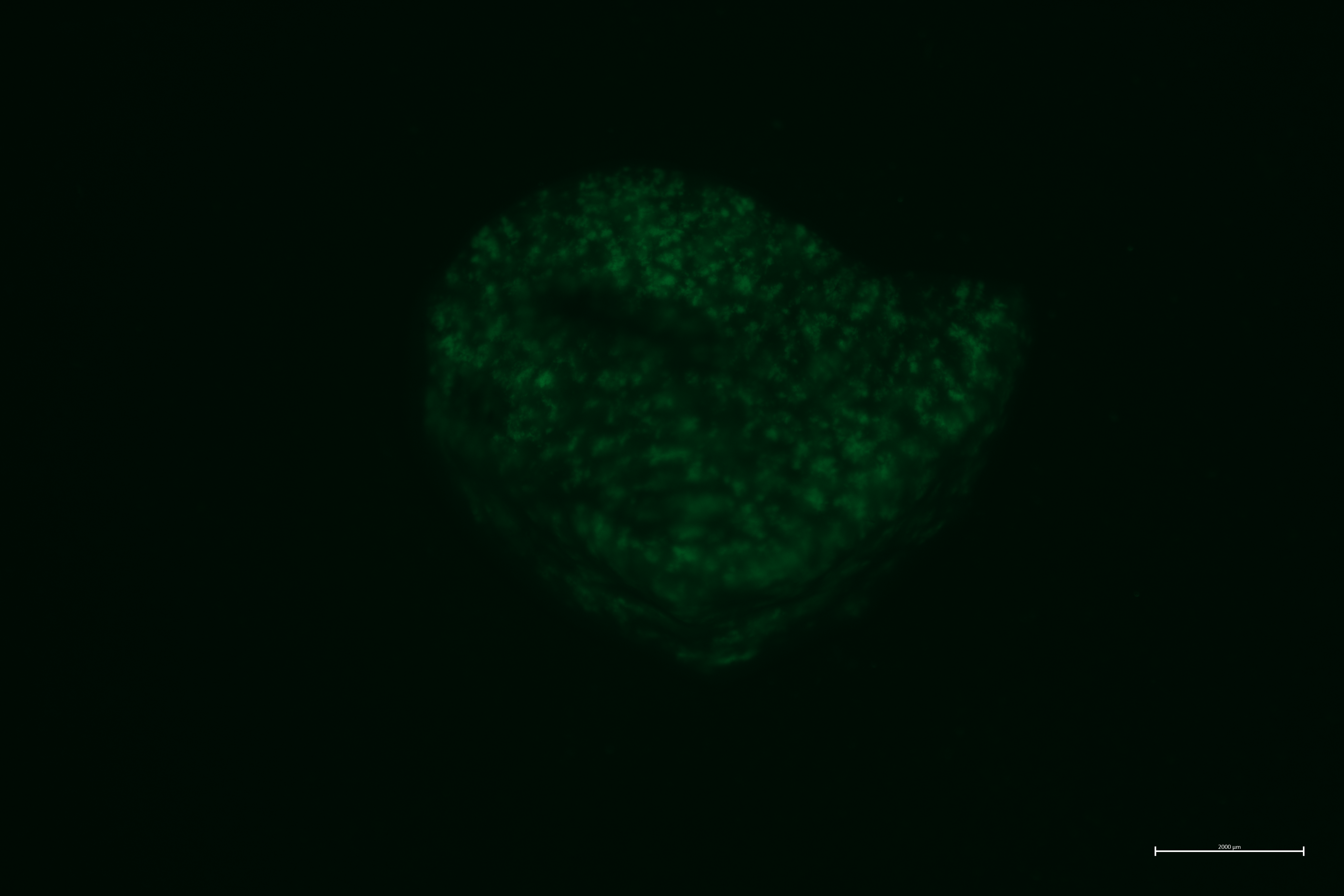

Supplement: Supplementary file 5 — Source data Fig. 5 [file 44319_2024_148_MOESM5_ESM.zip › Figure 5/Figure 5B/Liver_MosIR_400ms_EGFP.tif]

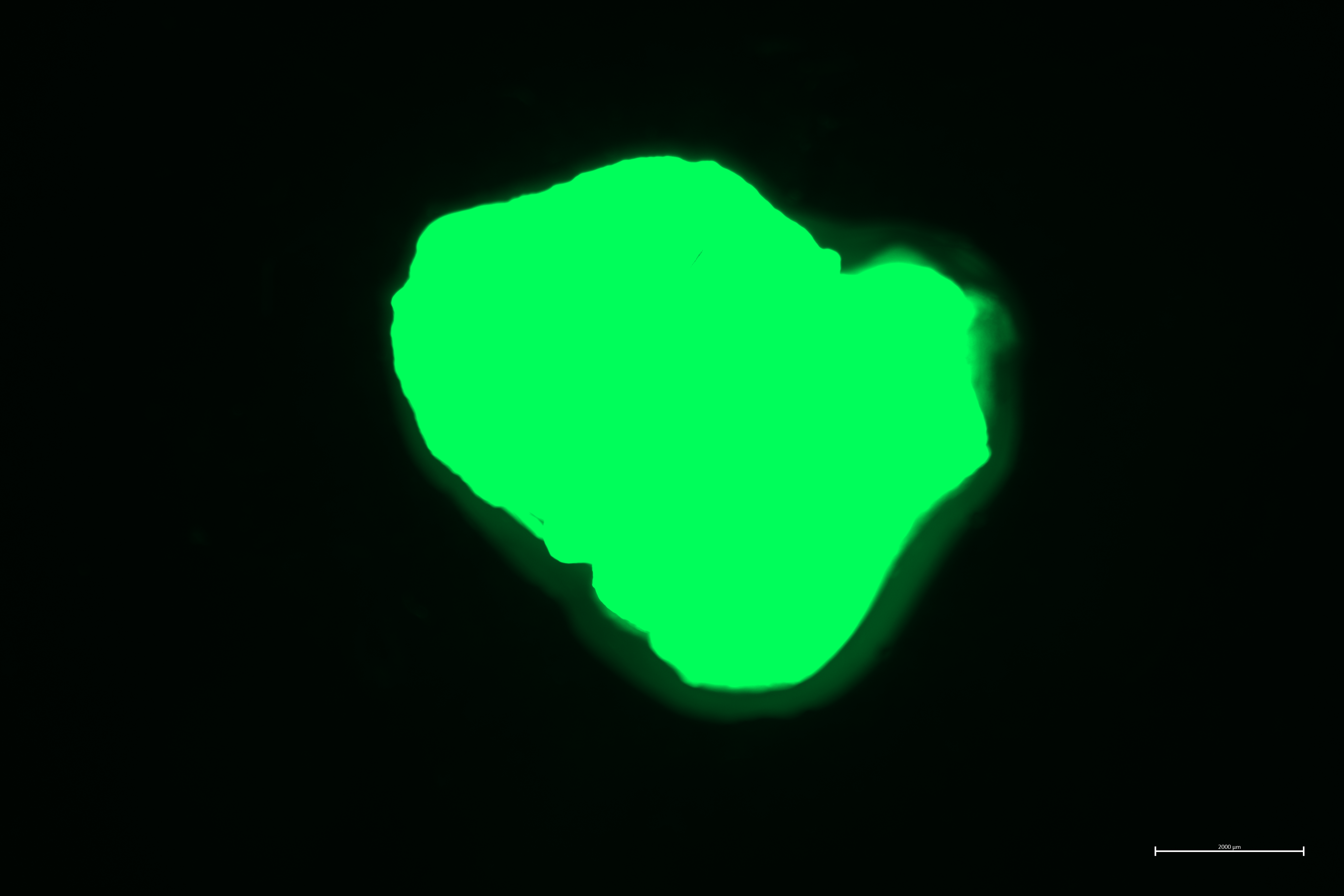

Supplement: Supplementary file 5 — Source data Fig. 5 [file 44319_2024_148_MOESM5_ESM.zip › Figure 5/Figure 5B/Skeletal_muscle_MosIR_400ms_EGFP.tif]

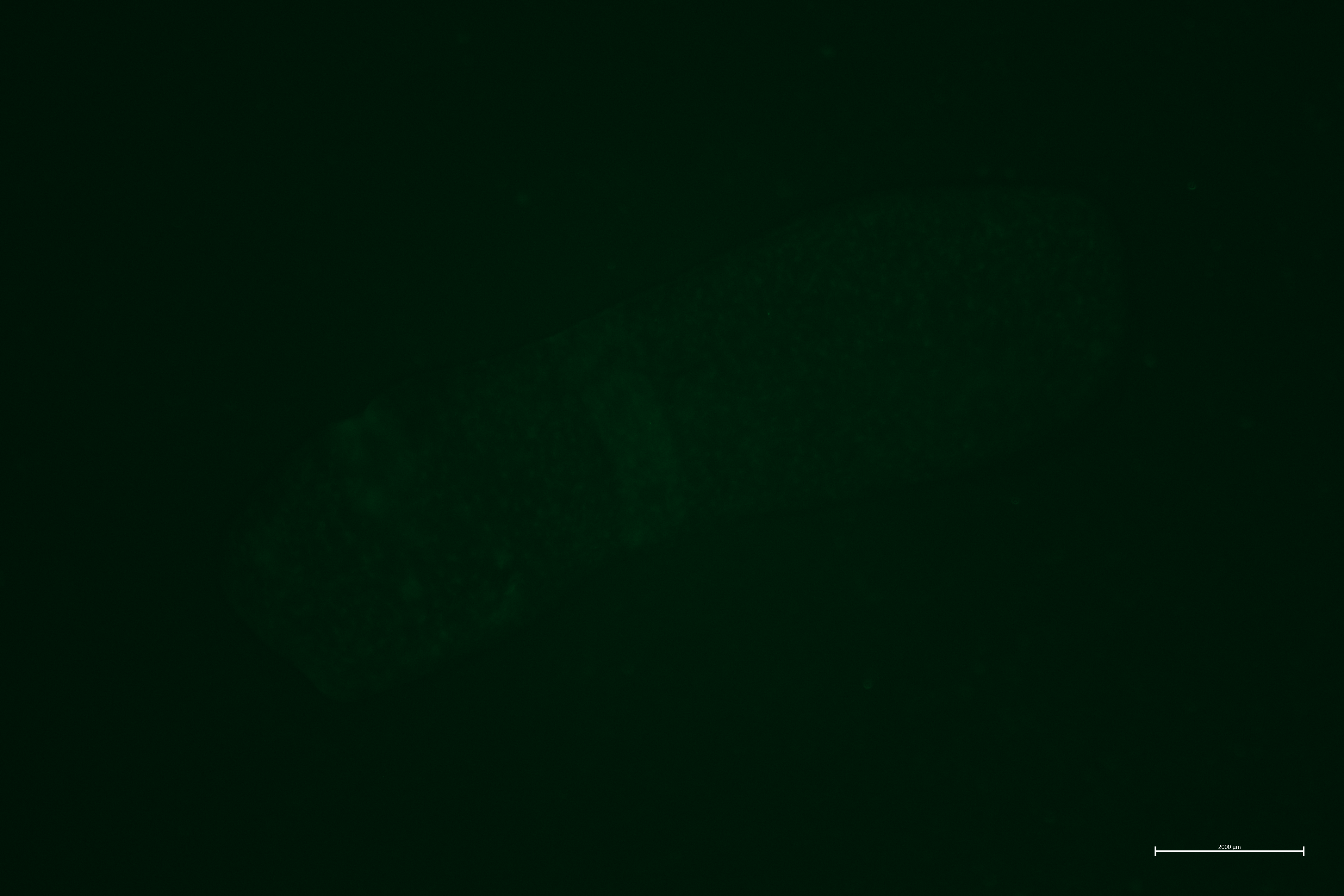

Supplement: Supplementary file 5 — Source data Fig. 5 [file 44319_2024_148_MOESM5_ESM.zip › Figure 5/Figure 5B/Spleen_MosIR_800ms_EGFP.tif]

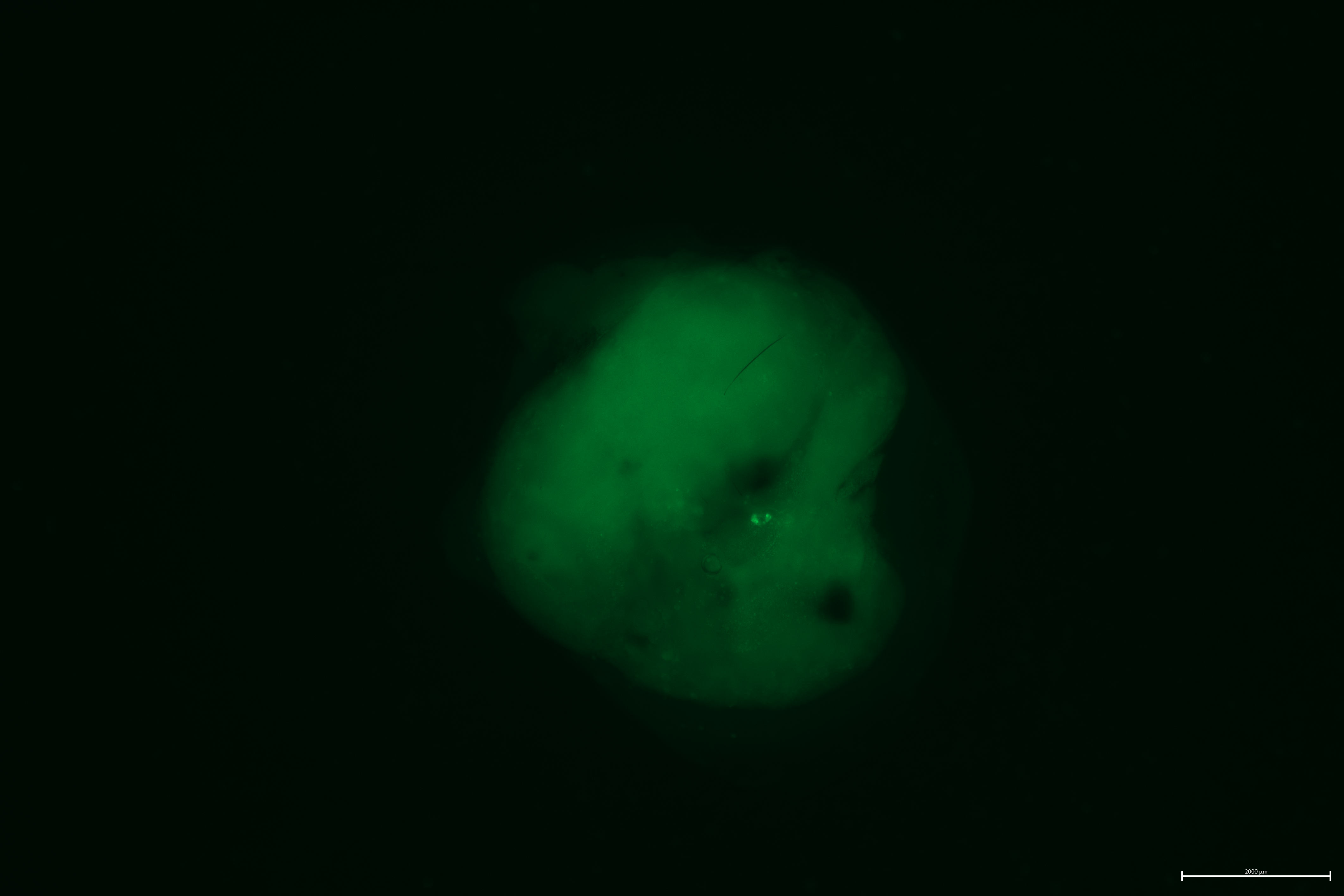

Supplement: Supplementary file 5 — Source data Fig. 5 [file 44319_2024_148_MOESM5_ESM.zip › Figure 5/Figure 5B/Thymus_MosIR_400ms_EGFP.tif]

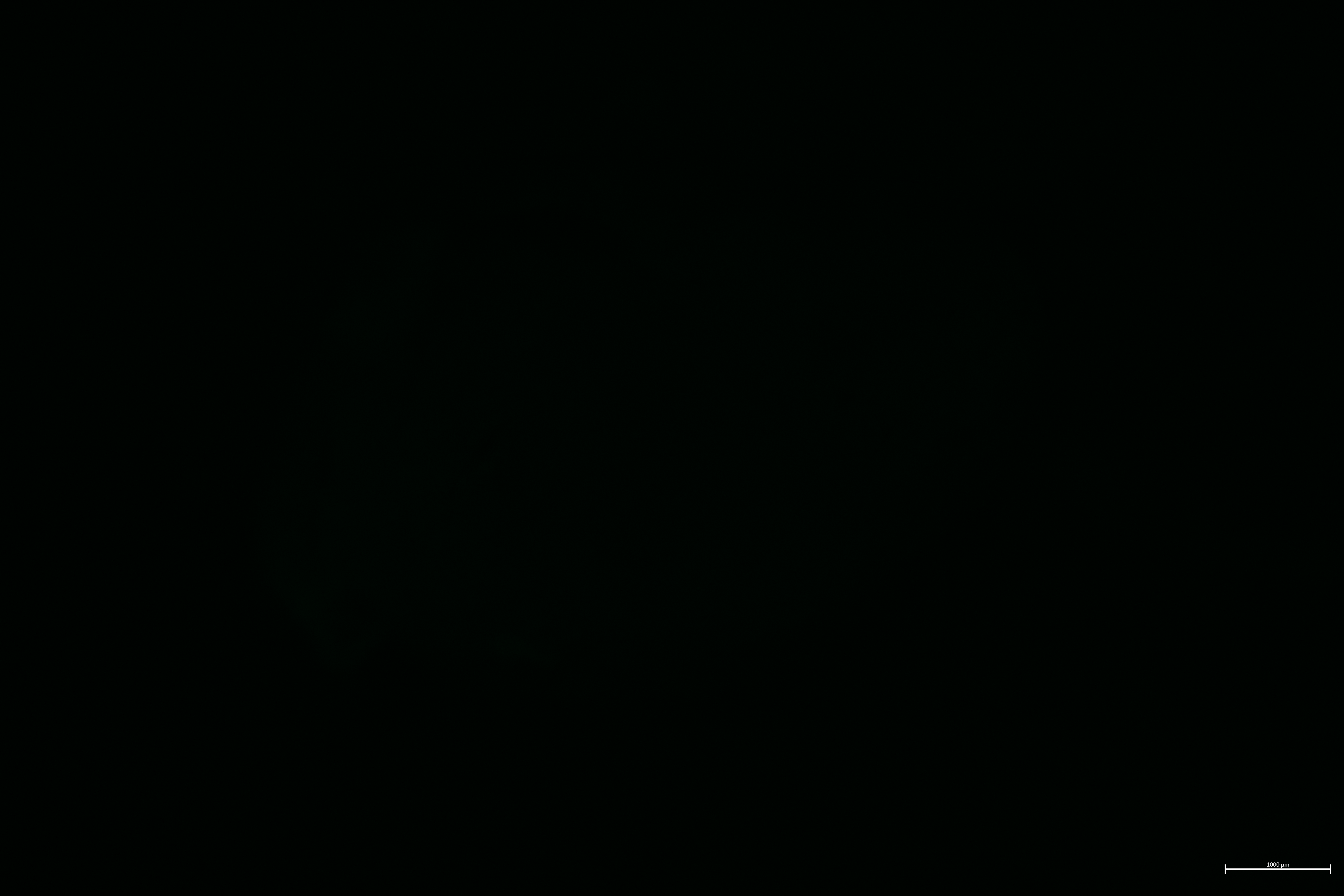

Supplement: Supplementary file 6 — Source data Fig. 6 [file 44319_2024_148_MOESM6_ESM.zip › Figure 6/Figure 6C/Heart_dHEL1_mCherry_EGFP.tif]

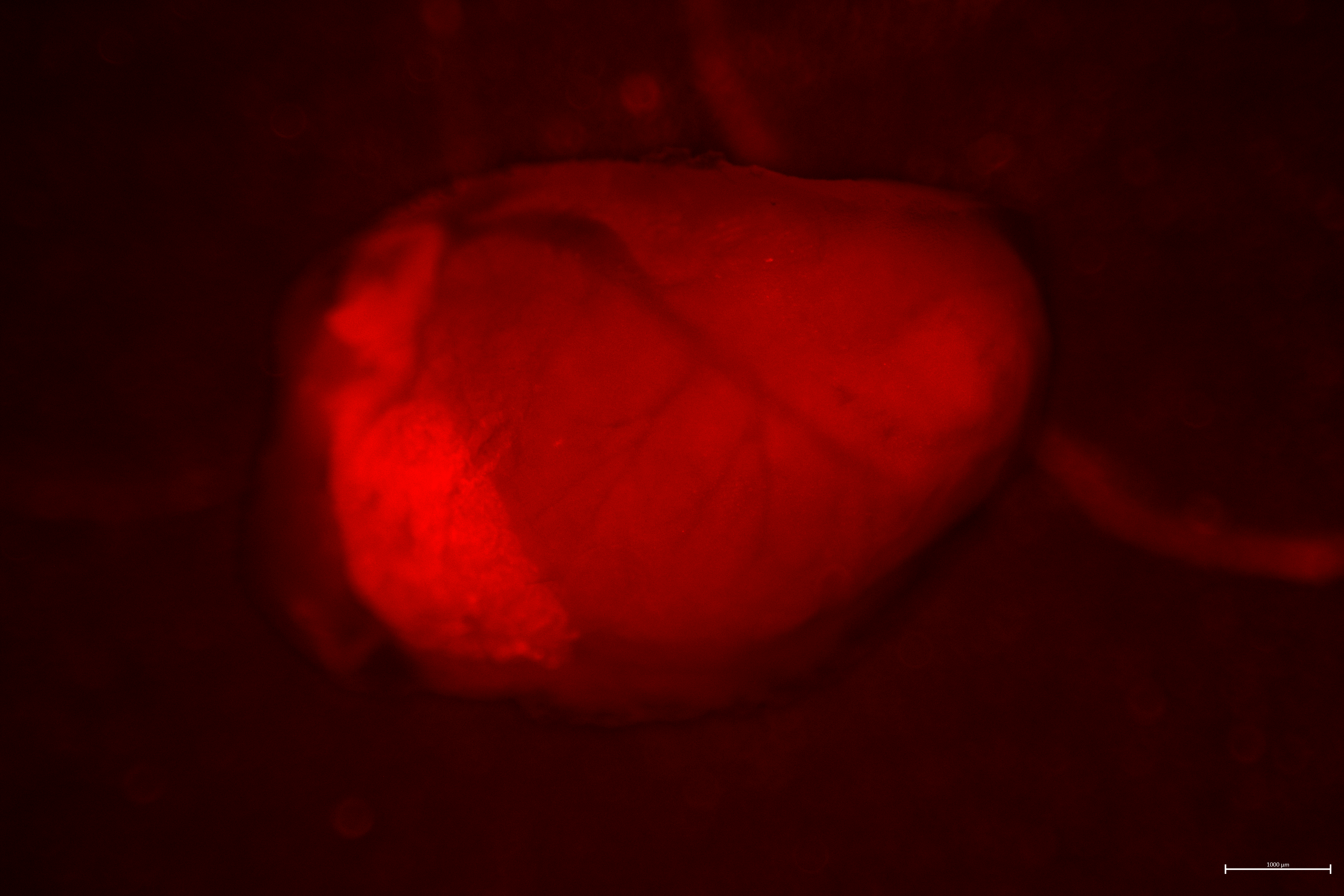

Supplement: Supplementary file 6 — Source data Fig. 6 [file 44319_2024_148_MOESM6_ESM.zip › Figure 6/Figure 6C/Heart_dHEL1_mCherry_mRF12.tif]

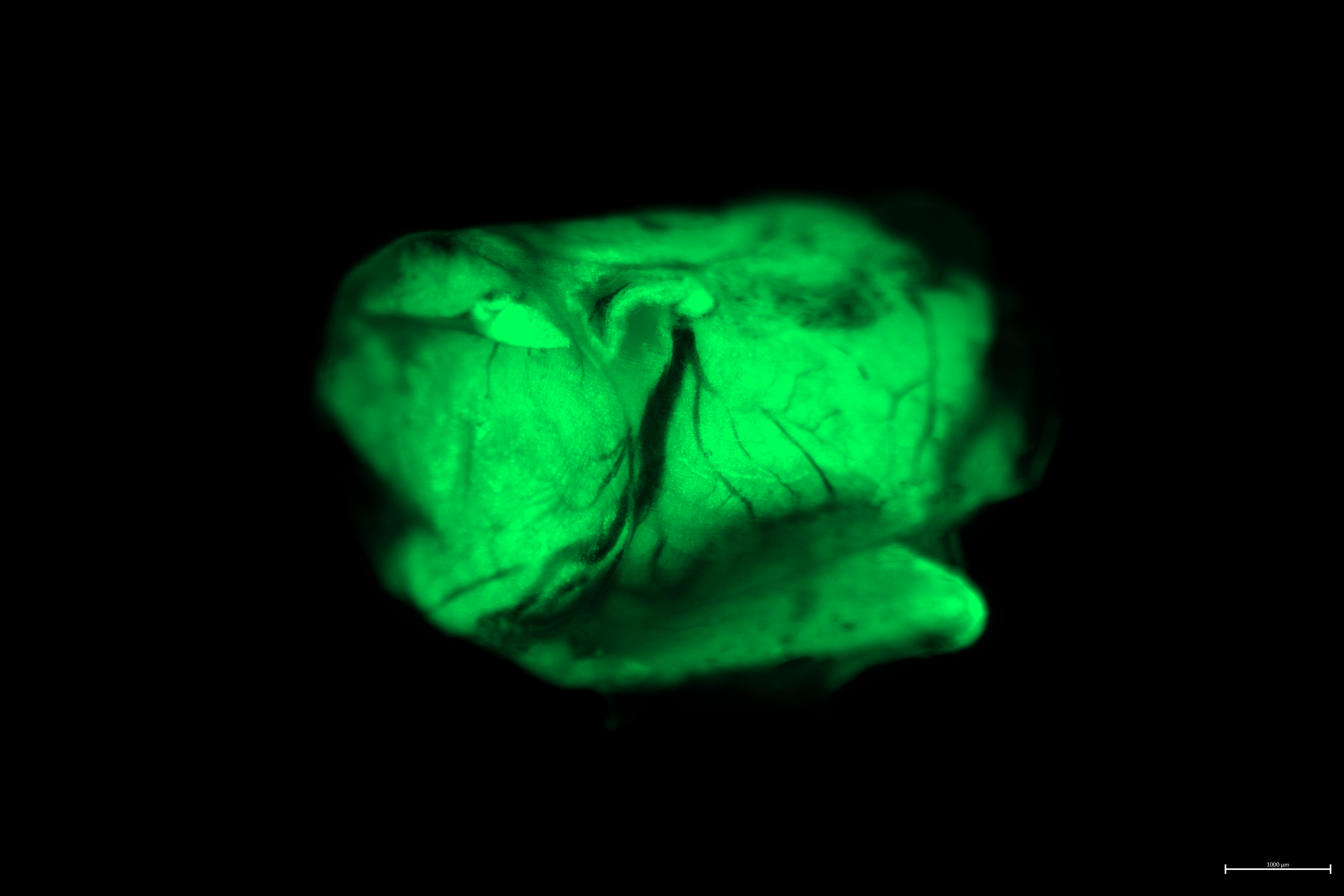

Supplement: Supplementary file 6 — Source data Fig. 6 [file 44319_2024_148_MOESM6_ESM.zip › Figure 6/Figure 6C/Heart_dHEL1_MosIR_mCherry_EGFP.tif]

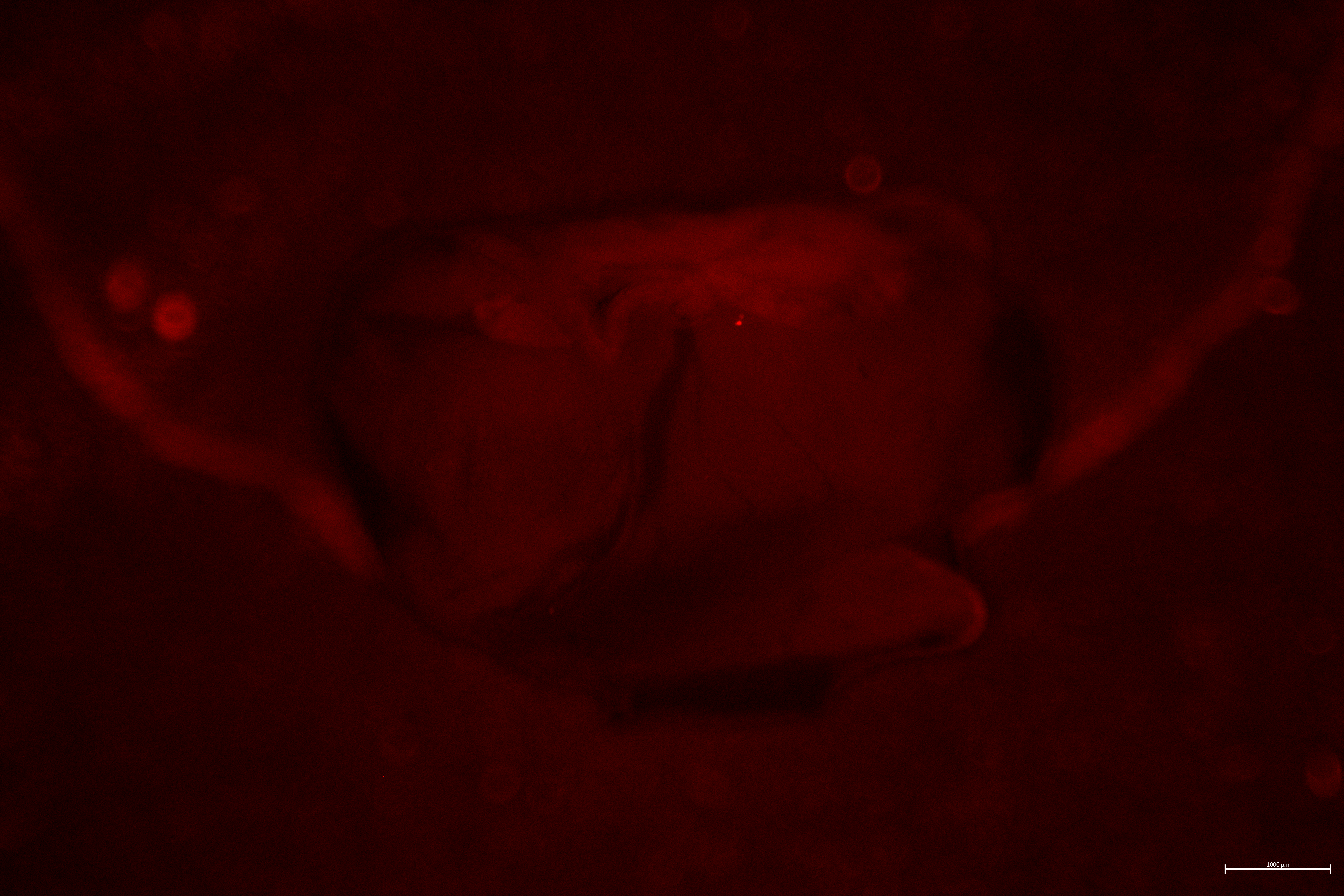

Supplement: Supplementary file 6 — Source data Fig. 6 [file 44319_2024_148_MOESM6_ESM.zip › Figure 6/Figure 6C/Heart_dHEL1_MosIR_mCherry_mRF12.tif]

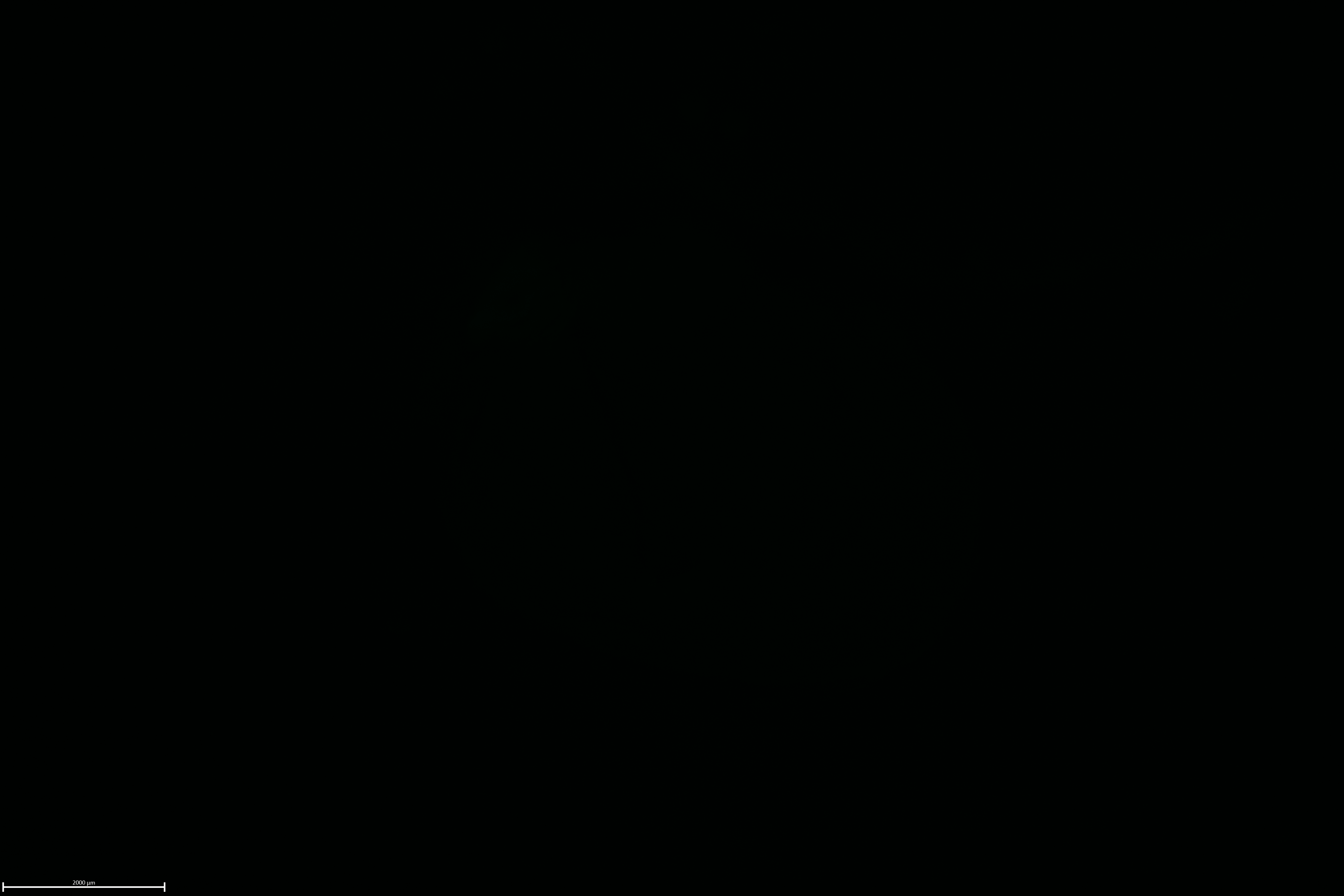

Supplement: Supplementary file 6 — Source data Fig. 6 [file 44319_2024_148_MOESM6_ESM.zip › Figure 6/Figure 6C/Heart_DicerSOM_mCherry_EGFP.tif]

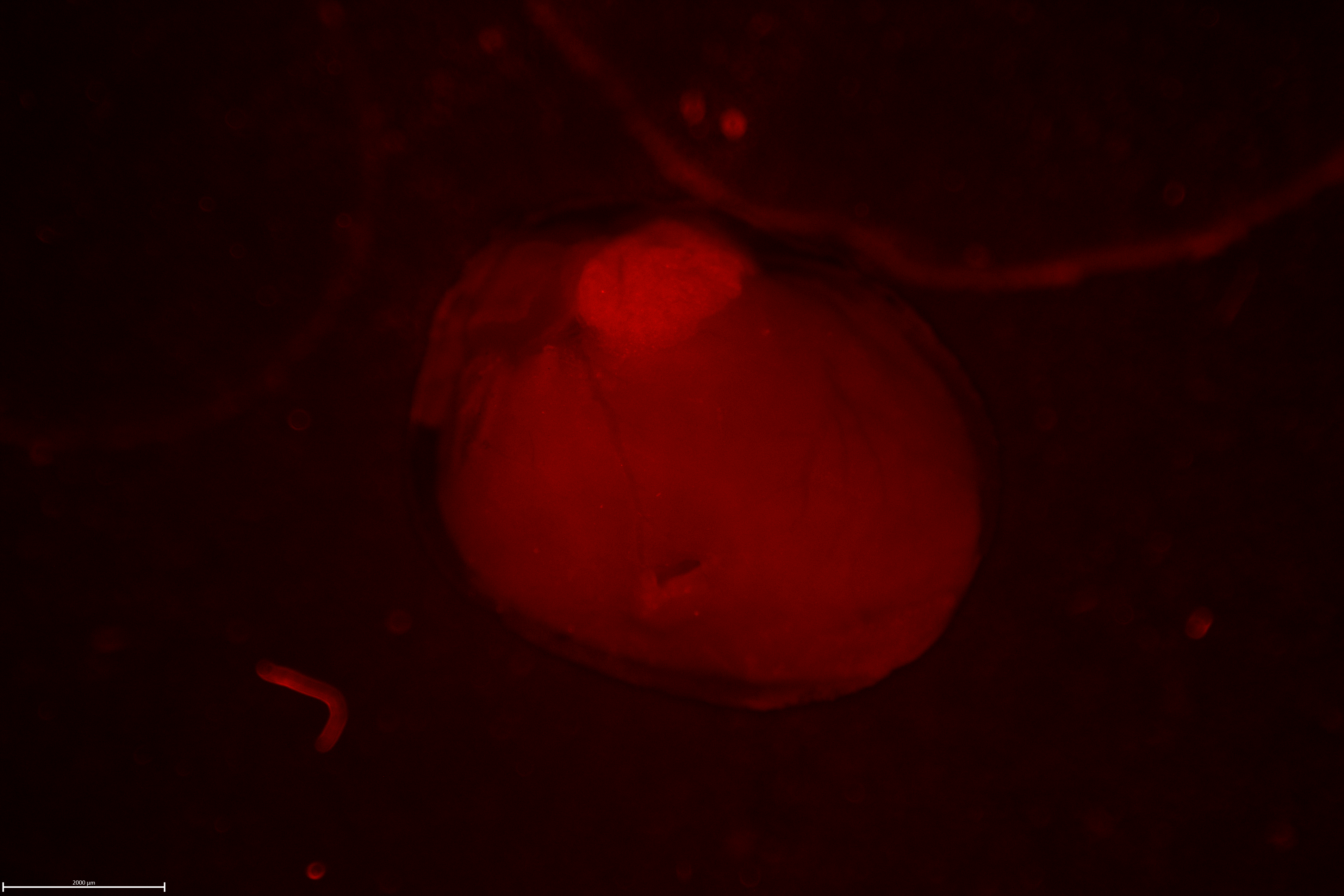

Supplement: Supplementary file 6 — Source data Fig. 6 [file 44319_2024_148_MOESM6_ESM.zip › Figure 6/Figure 6C/Heart_DicerSOM_mCherry_mRF12.tif]

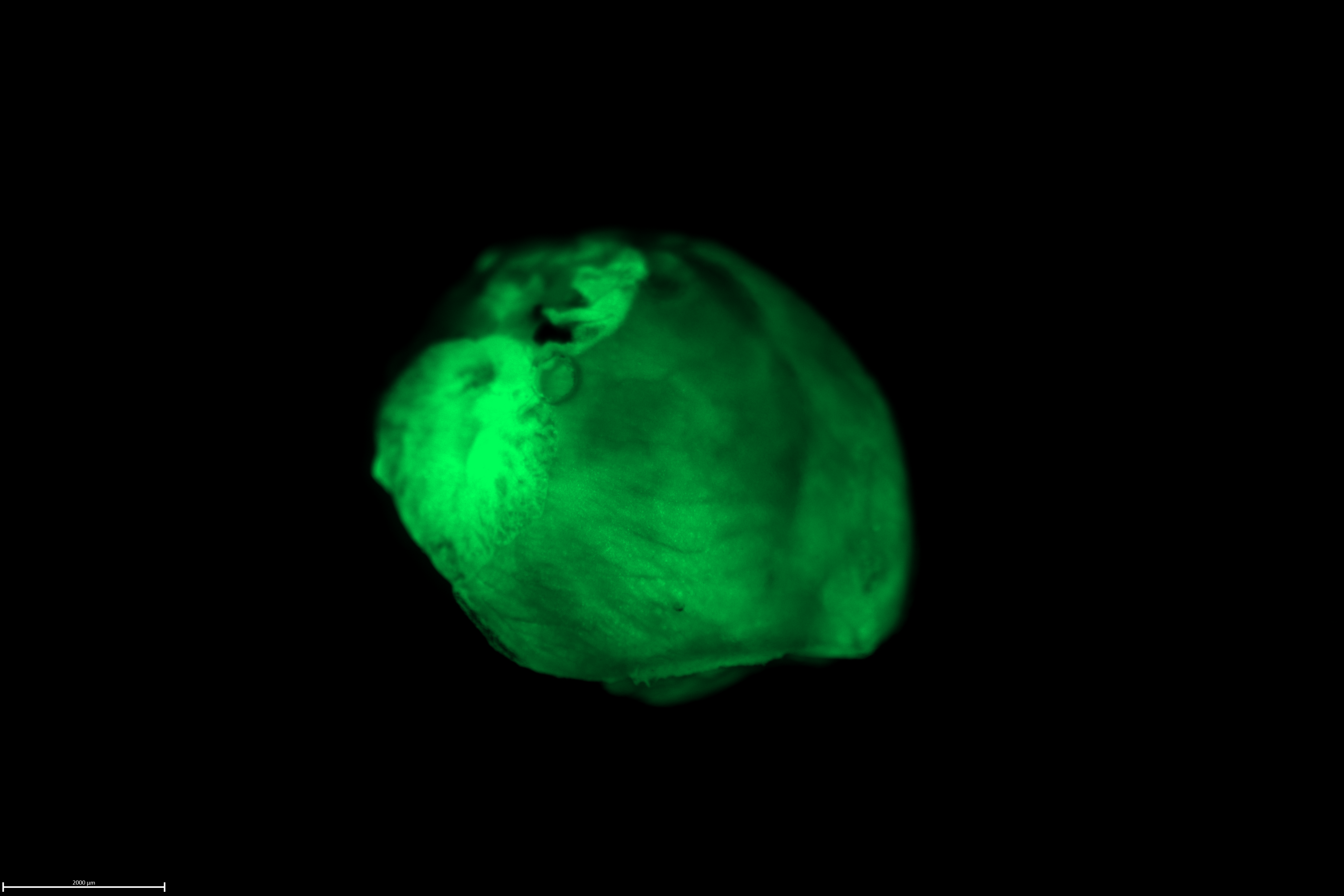

Supplement: Supplementary file 6 — Source data Fig. 6 [file 44319_2024_148_MOESM6_ESM.zip › Figure 6/Figure 6C/Heart_DicerSOM_MosIR_mCherry_EGFP.tif]

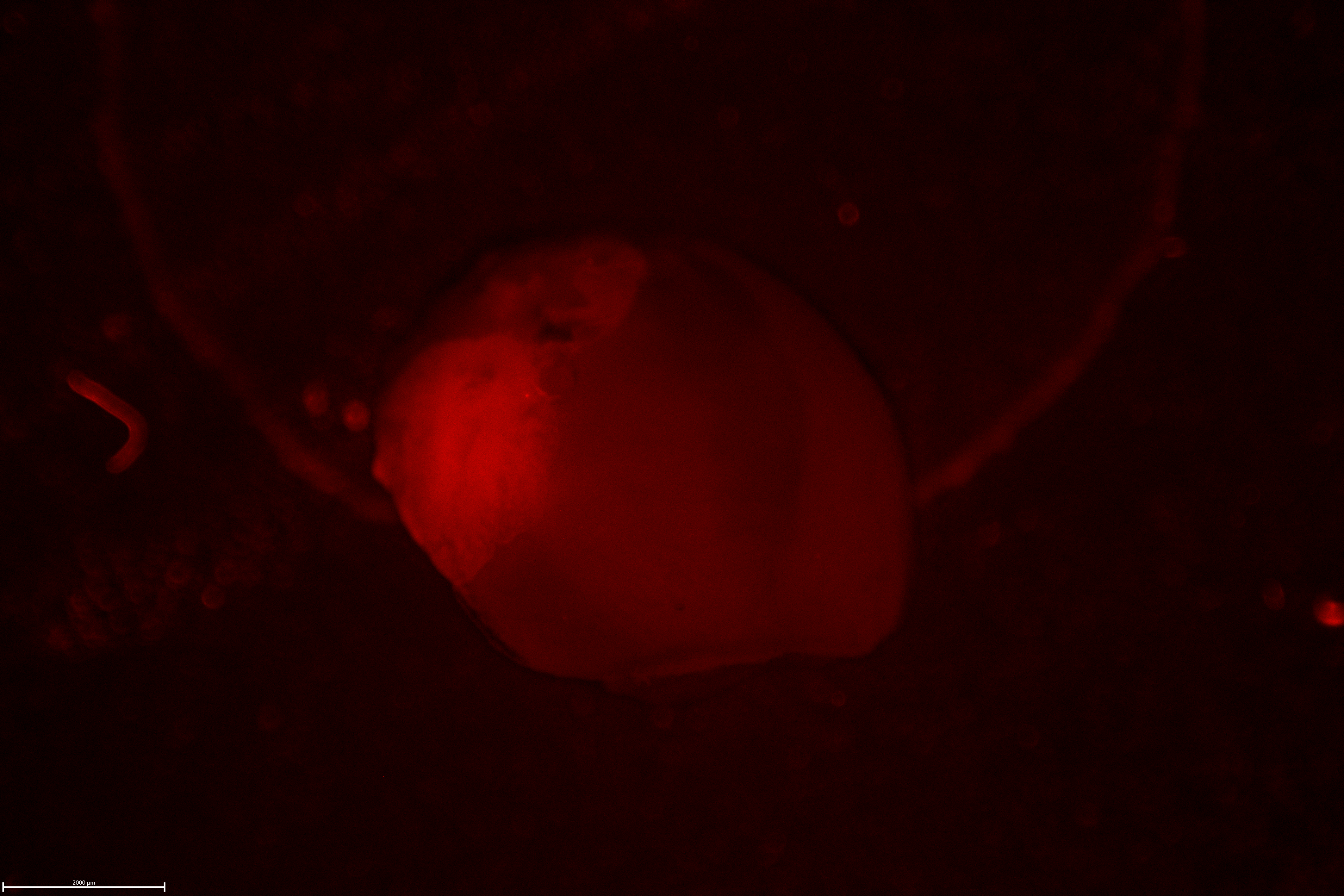

Supplement: Supplementary file 6 — Source data Fig. 6 [file 44319_2024_148_MOESM6_ESM.zip › Figure 6/Figure 6C/Heart_DicerSOM_MosIR_mCherry_mRF12.tif]

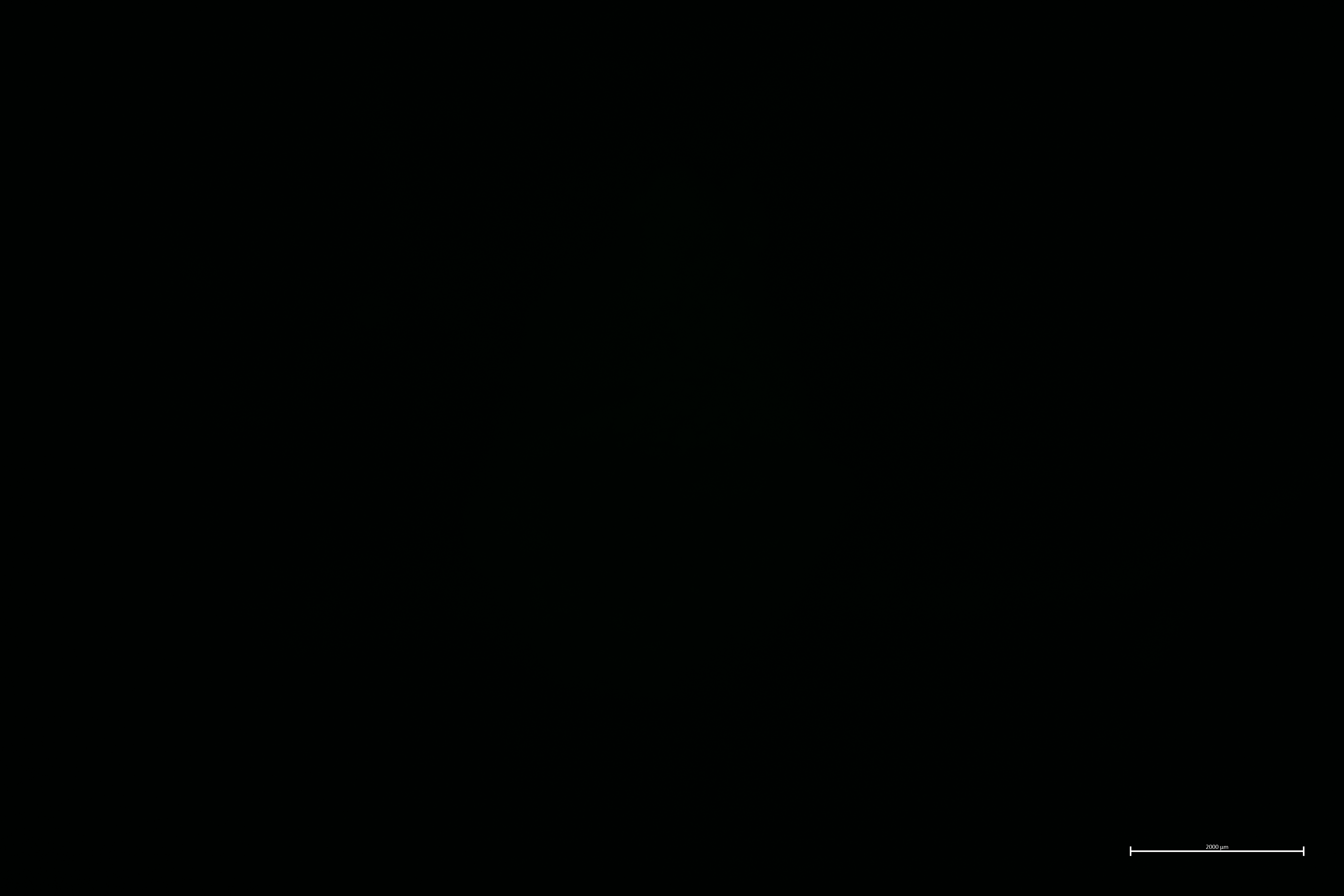

Supplement: Supplementary file 6 — Source data Fig. 6 [file 44319_2024_148_MOESM6_ESM.zip › Figure 6/Figure 6D/Skeletal_muscle_dHEL1_mCherry2_EGFP.tif]

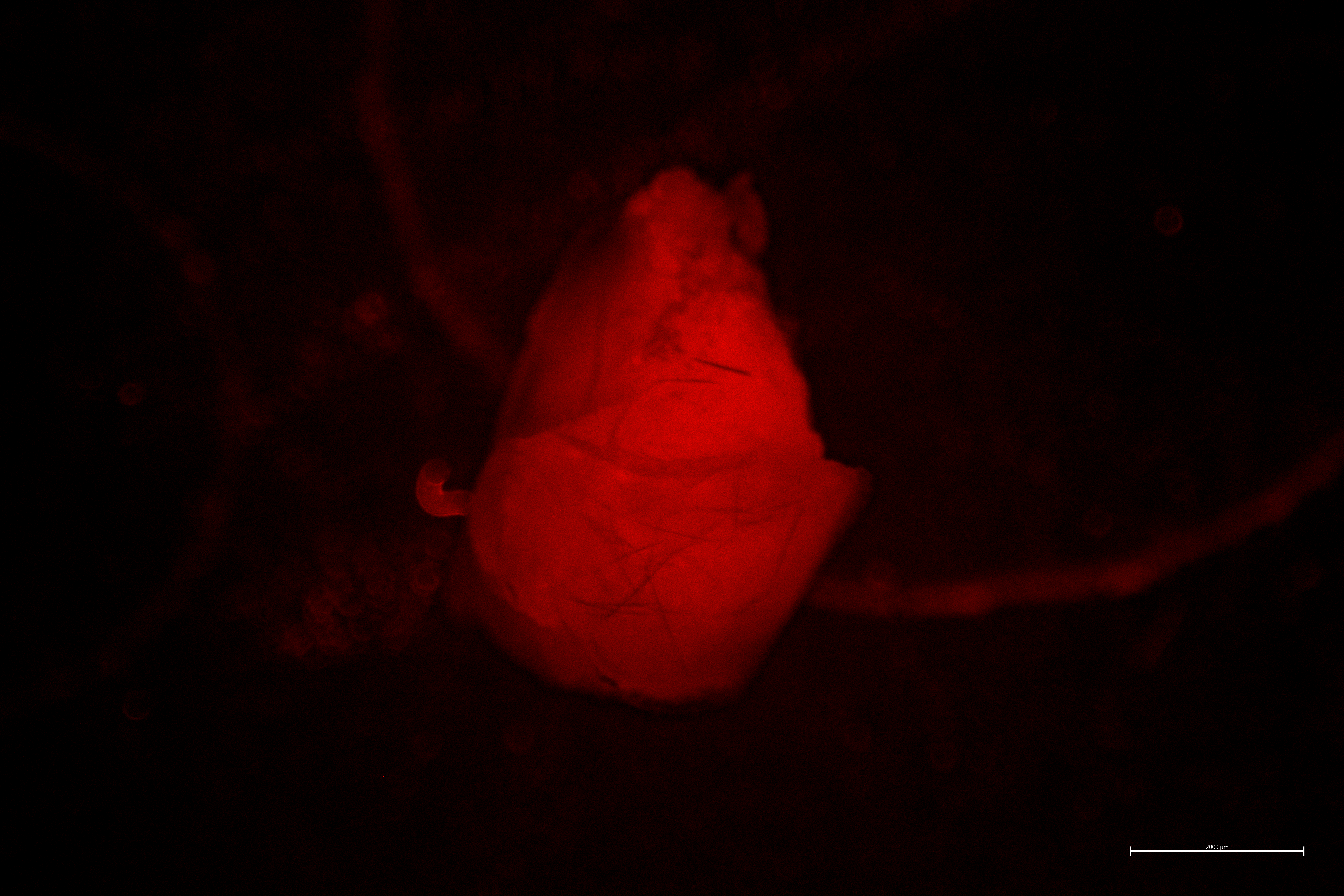

Supplement: Supplementary file 6 — Source data Fig. 6 [file 44319_2024_148_MOESM6_ESM.zip › Figure 6/Figure 6D/Skeletal_muscle_dHEL1_mCherry2_mRF12.tif]

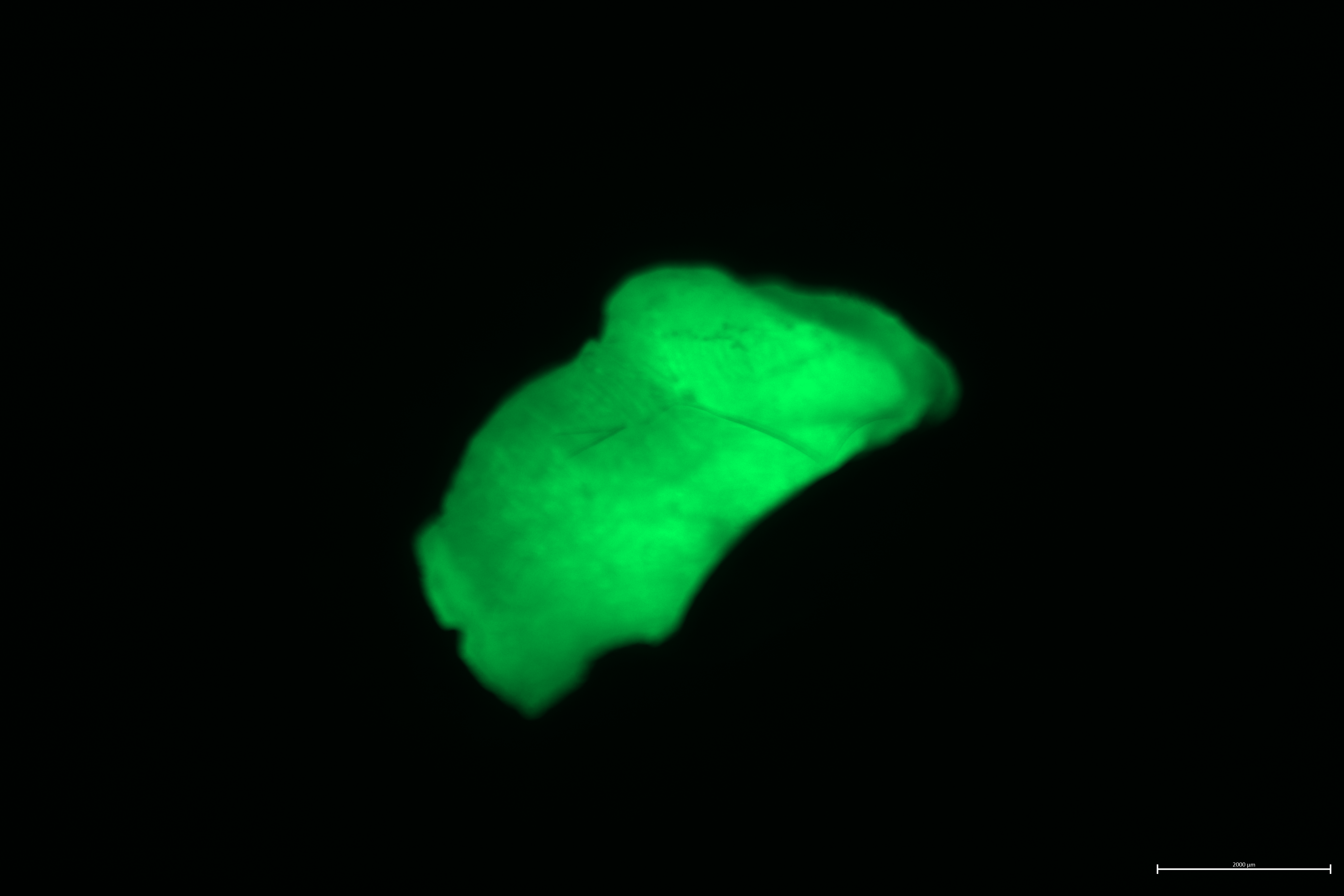

Supplement: Supplementary file 6 — Source data Fig. 6 [file 44319_2024_148_MOESM6_ESM.zip › Figure 6/Figure 6D/Skeletal_muscle_dHEL1_MosIR_mCherry2_EGFP.tif]

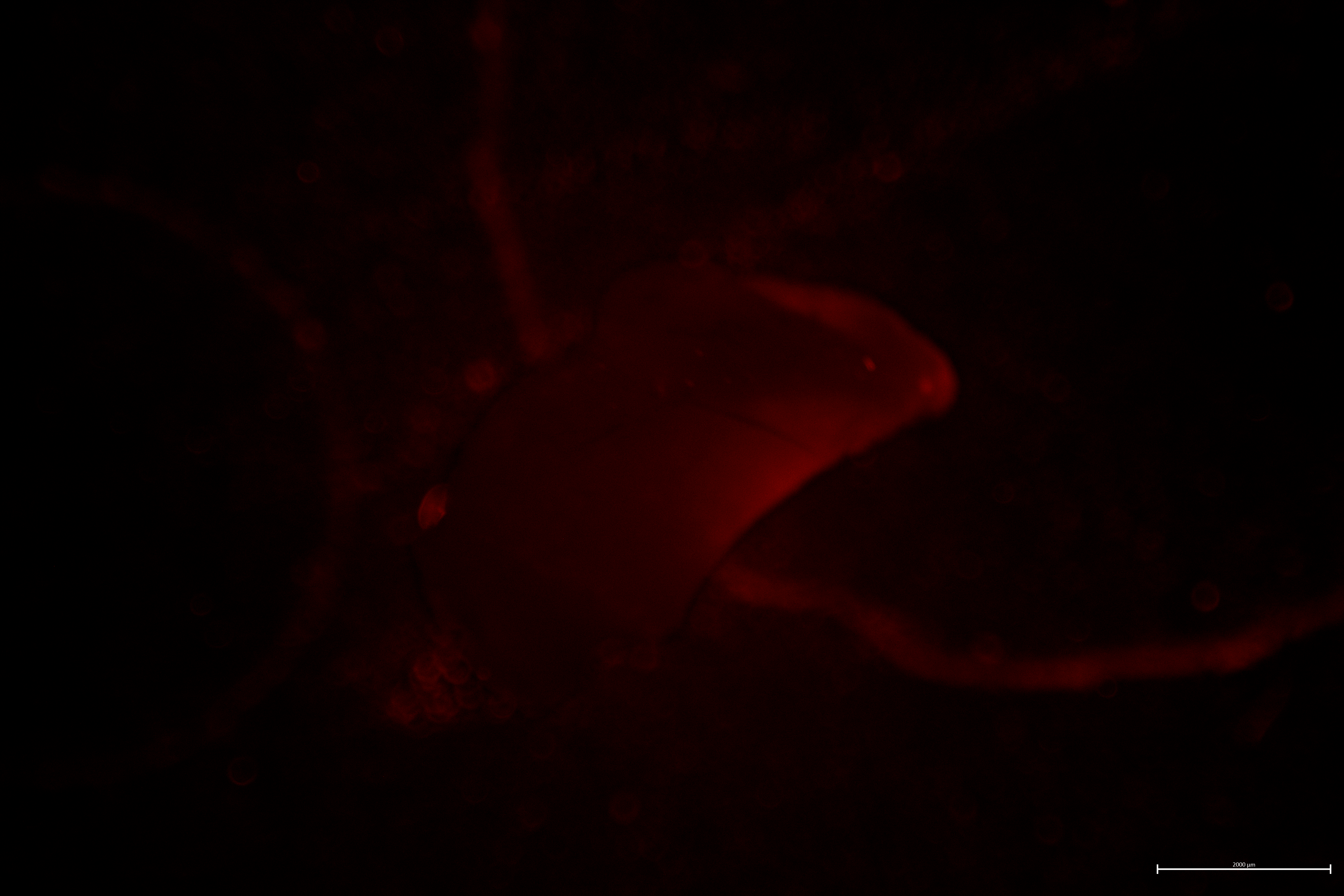

Supplement: Supplementary file 6 — Source data Fig. 6 [file 44319_2024_148_MOESM6_ESM.zip › Figure 6/Figure 6D/Skeletal_muscle_dHEL1_MosIR_mCherry2_mRF12.tif]

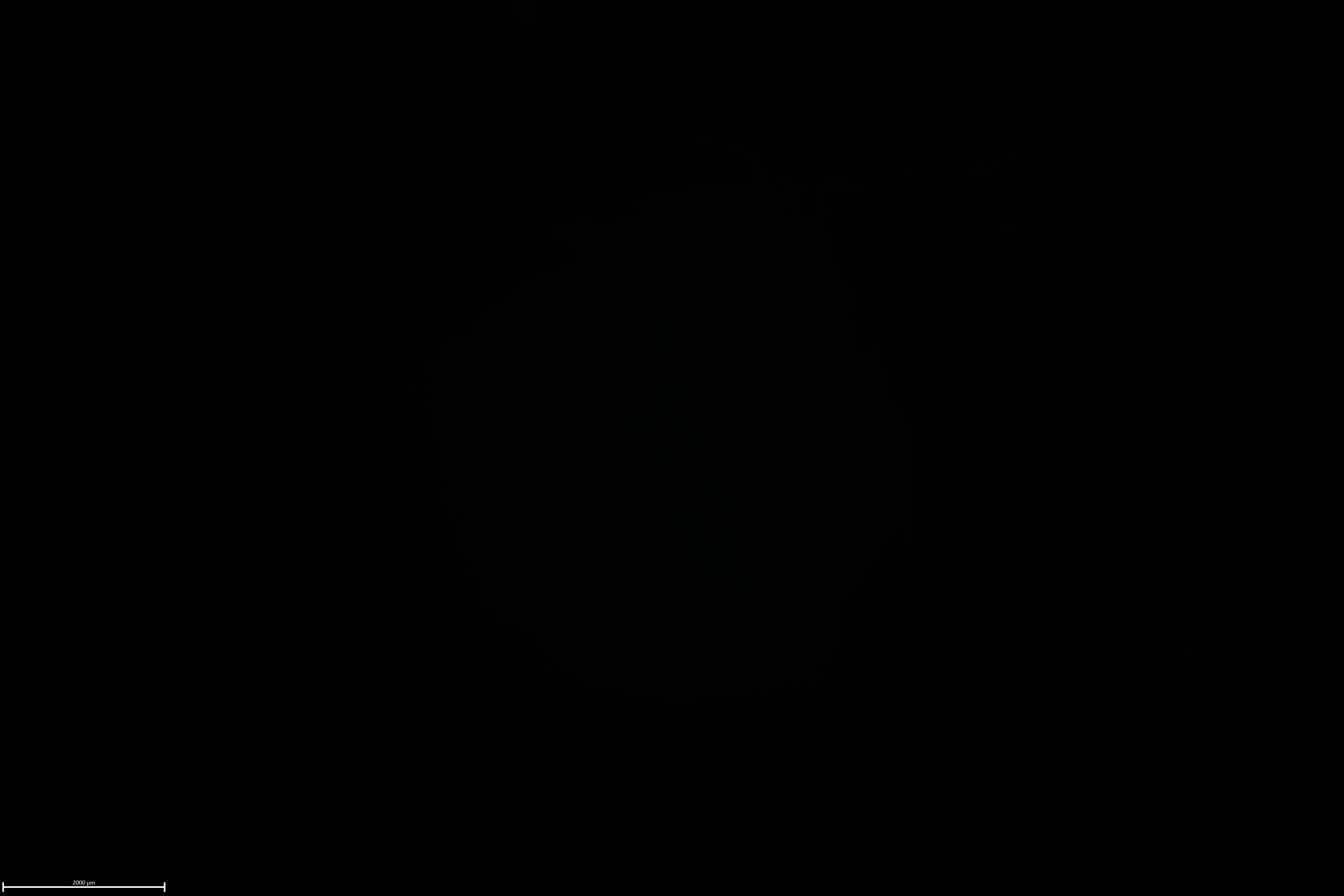

Supplement: Supplementary file 6 — Source data Fig. 6 [file 44319_2024_148_MOESM6_ESM.zip › Figure 6/Figure 6D/Skeletal_muscle_DicerSOM_mCherry_EGFP.tif]

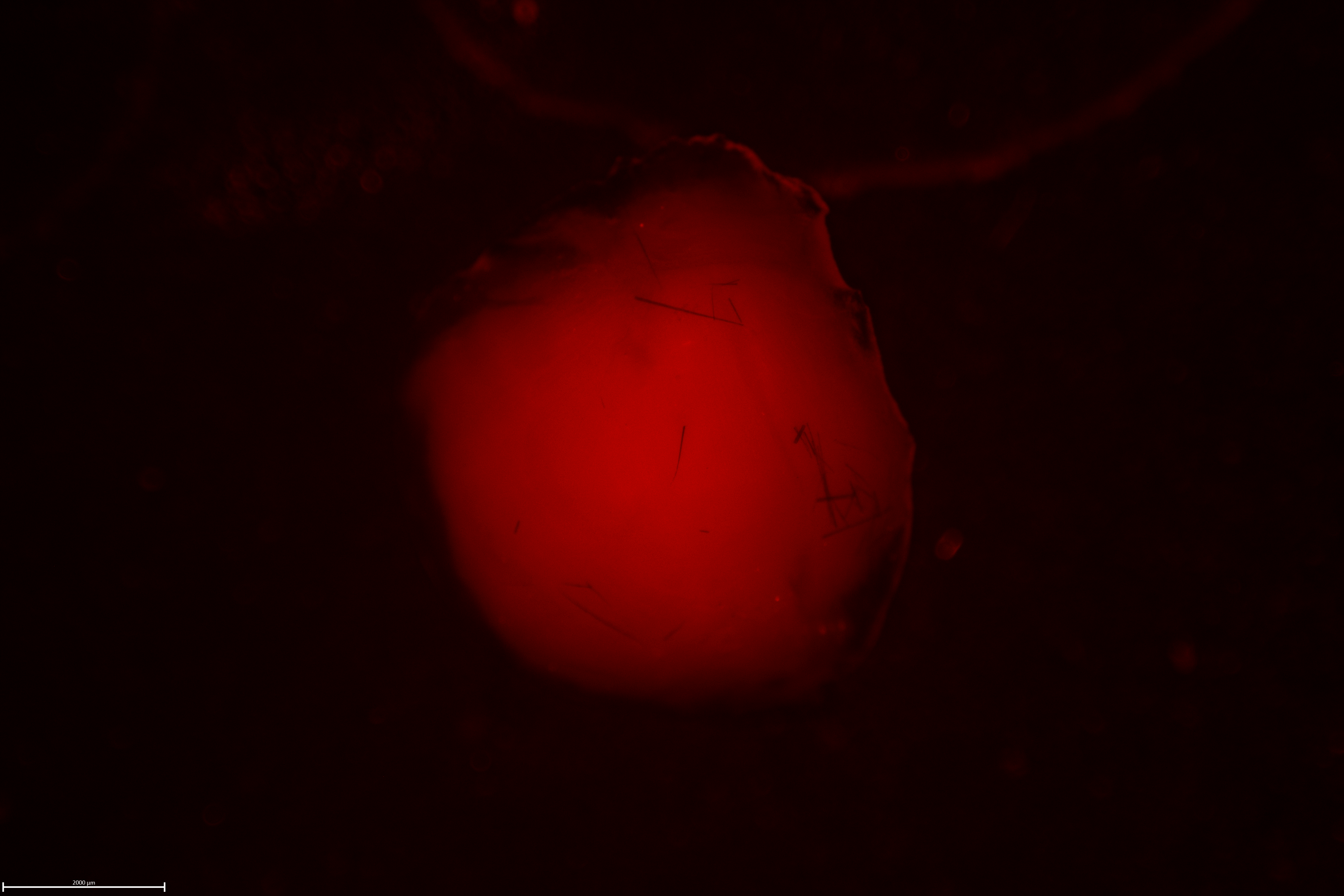

Supplement: Supplementary file 6 — Source data Fig. 6 [file 44319_2024_148_MOESM6_ESM.zip › Figure 6/Figure 6D/Skeletal_muscle_DicerSOM_mCherry_mRF12.tif]

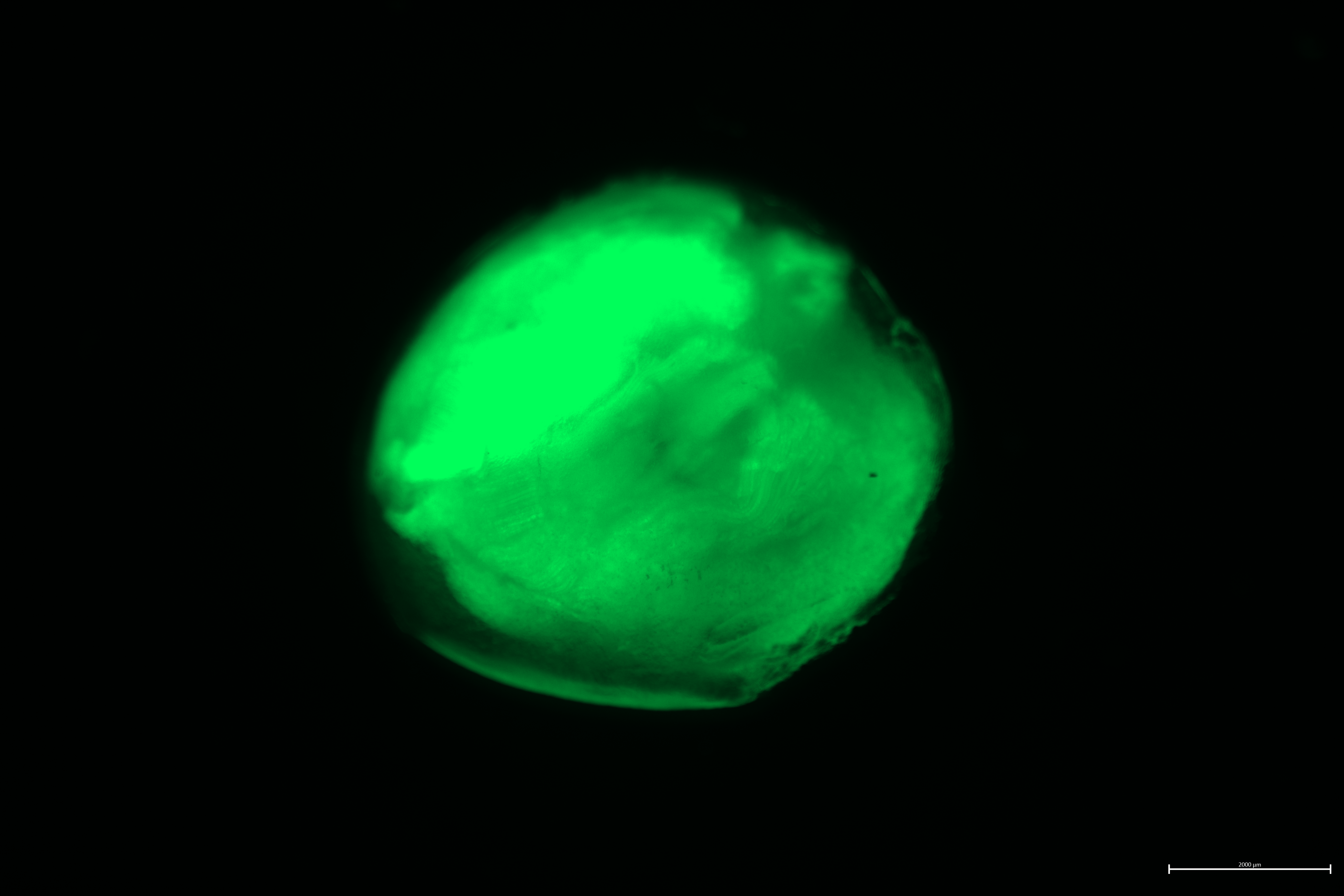

Supplement: Supplementary file 6 — Source data Fig. 6 [file 44319_2024_148_MOESM6_ESM.zip › Figure 6/Figure 6D/Skeletal_muscle_DicerSOM_MosIR_mCherry_EGFP.tif]

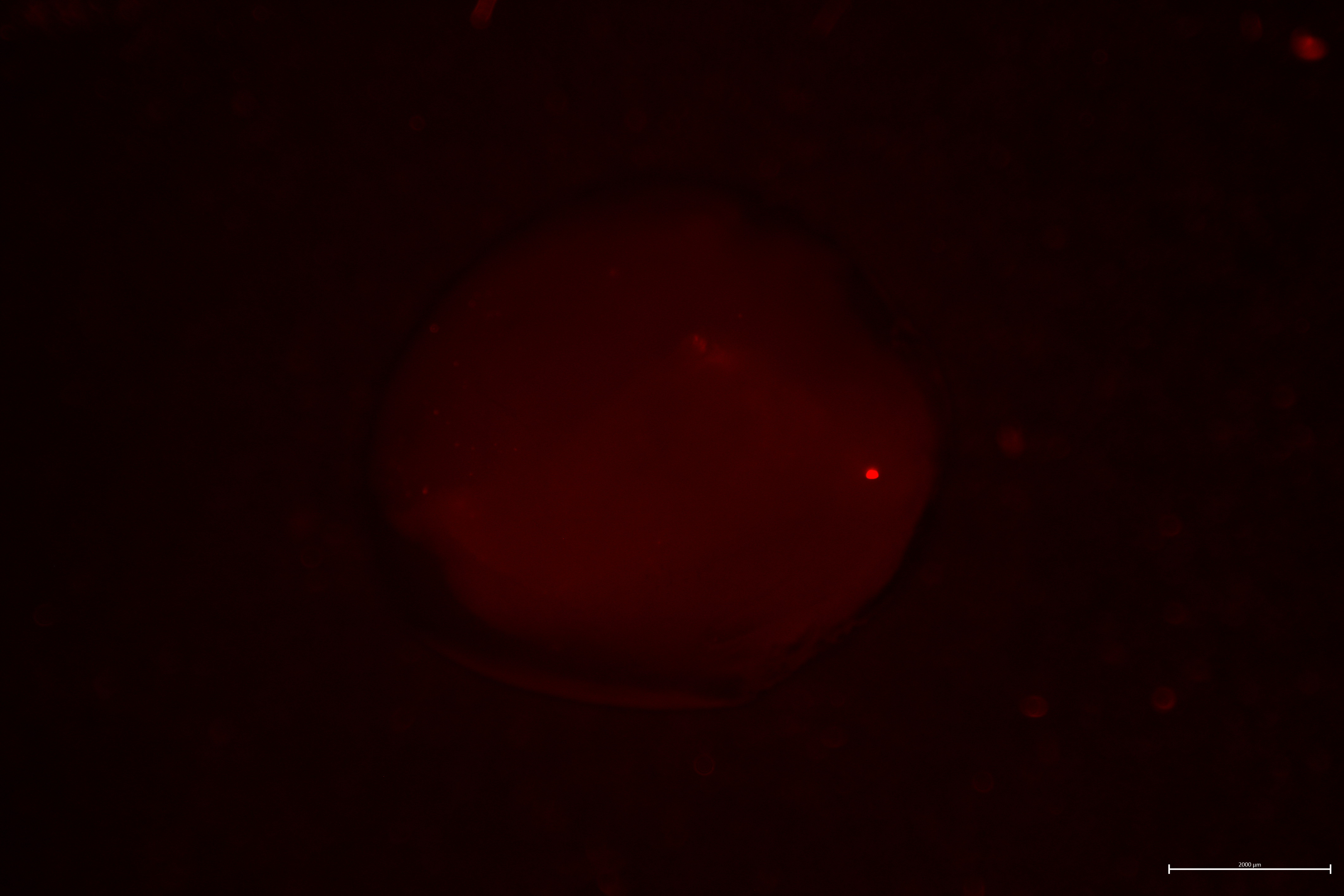

Supplement: Supplementary file 6 — Source data Fig. 6 [file 44319_2024_148_MOESM6_ESM.zip › Figure 6/Figure 6D/Skeletal_muscle_DicerSOM_MosIR_mCherry_mRF12.tif]
