# Supplementary material for: Functional canonical RNAi in mice expressing a truncated Dicer isoform and long dsRNA
Source: EMBO Rep. 2024 May 20;25(7):9. doi: 10.1038/s44319-024-00148-z (PMC11239679; doi:10.1038/s44319-024-00148-z)
Supplement: Supplementary file 7 — EV Figures Source Data [file 44319_2024_148_MOESM7_ESM.zip › Figure EV1/Source Data EV1.htm]

 

 

 

 
 
 


 \Delta Hel1 

 
 
 
 
 
 
 
 
 
 
 
 
 
 
 
 
 
 
 
 
 

 

 
 


 


 

 

 


 


 

 


 


 
 
 
 
 
 

 


 


  \(\Delta\) 
Hel1 

 


 
 
   
   
 The Czech Centre for Phenogenomics (CCP) is a large research
infrastructure dedicated to providing; first-class expertise, tools, and
services to reveal gene functions in human diseases, identifying new
therapeutic targets for treating human diseases and enhancing the
understanding of the genetic bases for human diseases. CCP was
established in 2015 as part of the wider Biotechnology and Biomedicine
Center of the Academy of Sciences and Charles University in Vestec
(BIOCEV) project and is hosted by the Institute of Molecular Genetics of
the ASCR, v. v. i. Located in the municipality of Vestec, Czech
Republic, CCP provides open access, state of the art services for both
the national and international research community. As a member of two
large consortia (IMPC and INFRAFRONTIER), CCP is committed to
discovering and ascribing biological function to each gene through the
generation of mutant mouse lines and use of the broad based primary
phenotyping pipeline which includes all the major adult organ
systems. 
  Director  : Radislav Sedlacek, Assoc. Prof., PhD 
  Phenotyping Module Head : Jan Prochazka, PhD 
 Czech Centre for Phenogenomics, BIOCEV (IMG), Prumyslova 595, 252 50
Vestec, Czech Republic 
 
  1  Primary Screening at
CCP 
 
  1.1  Staff Members 
 
 Biochemistry and Hematology 
  Head:  Roldan Medina De Guia 
  Technician(s):  Mariya Glushchenko, Eva Štefancová,
Yu-chieh Wu 
 
 
 Bioimaging and Embryology 
  Head:  Jan Prochazka 
  Specialist:  Frantisek Spoutil 
  Technician(s):  Michaela Prochazkova, Sarah Clewel,
Ivana Uramova, Tereza Michalcikova and Marie Kleisnerova 
 
 
 Cardiovascular 
  Head:  Jiri Lindovsky 
  Technician(s):  Petr Macek, Sarka Karbanova 
 
 
 Hearing 
  Head:  Jiri Lindovsky 
  Technician(s):  Miles J. Raishbrook, Jan Majernik 
 
 
 Histopathology 
  Head:  Dagmar Zudova, 
  Specialist(s):  Barbora Pavlu 
  Technician(s):  Linda Kutlíková, Christine Sophia
Carl Cañada, Aneta Cestrová,Thuy Lien Duongová, Attila Juhász 
 
 
 Immunology 
  Head:  Jana Balounova 
  Specialist(s):  Kristína Vičíková 
  Technician(s):  Kristyna Kunclova, Michaela
Simova 
 
 
 Lung function 
  Head:  
  Technician(s):  Vaclav Zatecka 
 
 
 Metabolism 
  Head:  David Pajuelo Reguera 
  Technician(s):  Rajasree Sain 
 
 
 Neurobiology and Behaviour 
  Head:  Agnieszka Kubik-Zahorodna 
  Technician(s):  Pavel Jina, Rozalie Novakova,
Katarina Kanasova, Pavlina Kucerova 
 
 
 Vision 
  Head:  Marcela Palkova 
  Specialist:  Jiri Lindovsky 
  Technician(s):  Viktoriia Symkina 
 
 
 Bioinformatics 
  Head:  Vendula Novosadova 
  Specialist:  Julia Potip, Frantisek Malinka and
Carlos Trufen 
 
 
 
  1.2  Animal Care 
 At CCP, mice are housed in GM500 cages in individually and digital
ventilated caging (IVC and DVC) systems (IVC and DVC System Green Line,
Tecniplast, Italy) on aspen bedding (Tapvei, Estonia). The IVCs operate
with positive pressure. Mice are transferred to new cages at 2 week
intervals in Laminar Flow Class II changing stations (Tecniplast,
Italy). Mice are fed with autoclaved standard rodent high energy
breeding diet (Altromin 1314 Forti) and given reverse osmosis filtered
and chlorinated water ad libitum. Light is adjusted to a 12h/12h
light/dark cycle; temperature and relative humidity are maintained at
22±1 °C and 55±5 %, respectively. In specified modules husbandry
conditions are adjusted according to the experiment requirements. All
materials entering the breeding and experimental area are decontaminated
by autoclaves or by Vaporized hydrogen peroxide (VHP). All persons
entering the facility change their clothes (autoclaved jackets, trousers
and socks) and shoes, wear caps, masks and gloves before entering the
CCP facility. Entry into the facility is by wet or air shower depending
on the barrier status of the designated room. 
 Outbred 6-week-old SPF CD 1 mice are used as sentinels and kept on a
mixture of soiled bedding from all cages of the IVC racks connected to
one IVC blower. Health monitoring is carried out by outsourced service
of the sentinel mice by certified laboratories according to FELASA
recommendations  http://www.felasa.org . 
 All animals are kept according to Directive 2010/63/EU and Czech
legislation. 
 
 
  1.3  Quality
Assurance 
 All experiments and investigations carried out at the Czech Centre
for Phenogenomics (CCP) are conducted according to standard operating
procedures (SOPs) and with approval of the IMG ethics committee. 
 All data generated is validated by CCP’s expert scientists who check
the biological and mathematical relevance of the data. 
 The constant development of staff is a vital part of CCP’s quality
assurance. To this end, CCP staff members are cross trained in multiple
techniques and regularly attend workshops, seminars and conferences to
further develop their skills and knowledge. 
 
 
  1.4  Workflow 
 The Czech Centre for Phenogenomics standard primary workflow follows
the international mouse phenotyping consortium (IMPC) pipeline. 
   
   
 
 
  1.5  Statistical
Analysis 
 All numerical univariate data are analyzed using generalised mixed
model. The fixed variables are always Genotype, Gender, in the case that
data are dependent on weight the other fixed variable is weight, for
time series data the last fixed variable is time. The random variables
are Date and Experimenter. Categorical data are analyzed by Fisher test
with grouping WildType Female, WildType Male, Knockout Female, Knockout
Male. 
   
 
 
 
  2  Standard Screening
Procedures 
 
  2.1  Viability and
Fertility 
 Purpose of these tests is to assess the fertility of homozygous
knockout mice and the postnatal viability, sub-viability, and lethality
of homozygous mice during cohort production. 
 For fertility screen homozygous mice (minimum age of 8 weeks, maximum
age of 14 weeks) are mated for 4-6 weeks. Strains that produce no
progeny or pregnant dams after 4 to 6 weeks progress to secondary
screening. 
 For viability screen mice are monitored for their genotypes after Het
X Het breeding. At least 28 pups are genotyped. Then strains which
produce no homo/hemizygotes are considered as  lethal .
Strains that produce less than normal numbers of homozygous/hemizygous
male or female pups are considered as  subviable . 
 
 
  2.2  Body Weight 
 The body weight of each mouse is to be measured weekly, from the 4th
until the 16th week. Optional additional weighs may also be
recorded. 
 
 
  2.3  Neurobiology and
Behaviour 
 Neurobehavioral tests using transgenic animal models make it possible
to understand genetic mechanisms underlying neurological and psychiatric
disorders including, but not limited to, anxiety, schizophrenia, mood
disorders, and Parkinson’s disease. To understand the mechanisms
underlying these disorders we employ a number of tests which analyse
motor abilities, cognitive functions, emotion, sensory processing as
well as neurological, gait, auditory, and vision screens of rodents. 
 All animals are adapted to the testing room for 1hr prior to testing.
Before placement of an animal in any test, the equipment is cleaned with
alcohol to remove olfactory traces. 
 
  2.3.1  Animal emotionality
and affect 
 To study animal emotionality and affect we put into service tests
like Open Field, Elevated Plus Maze, Light/Dark box, Tail Suspension
Test, and Forced Swim Test. 
  Open Field  
 The open Field test is conducted according to  IMPC
protocol  and evaluates animal motility triggered by exploratory
drive in a new environment (Welker 1959). It is also used as an initial
screen for general anxiety elicited in a well-lit, open unprotected
space (Whimbey &amp; Denenberg 1967). Detailed and fully automated
analysis of animal behaviour in open space is based on video tracking
system (Viewer, Biobserve GmbH). The software distinguishes an animal as
an object contrasting with the background (Fig.1). To obtain the
sufficient contrast of diversely pigmented animals with simultaneous
preservation of experimental conditions we are using infrared light
built into the base, on which all IR translucent mazes are placed.
Testing apparatus is uniformly illuminated with the light intensity of
200 lux in the centre of the field. Simultaneously four animals are
tested in four separated open fields for 20 min. Testing arena is
virtually divided to periphery and centre zones, where centre zone
constitutes 38% of whole arena. Presence time, distance travelled, no.
of visits, latency to enter centre zone, average speed, resting and
activity time, velocity classes, and many others indices are
automatically computed for periphery and centre zone as well as for each
5 min. interval. 
 
   Fig. 1:  Tracking the
animals in open fields
 
   
  Light-Dark Box  
 LDB is divided into two compartments; the light part and enclosed
dark one. The dark part constitute of one third of total apparatus area.
The light part is uniformly illuminated with the light intensity of 430
lux. Each animal is allowed to freely explore the EPM for 10 minutes.
Presence time, distance travelled, no. of visits, latency to enter,
average speed, resting and activity time, velocity classes, and many
others indices are automatically computed for both box’s
compartments. 
 
 
  2.3.2  Neuromotor
Abilities 
 Animal sense of balance and motor coordination can be evaluated in
Beam Walk test and Rotarod, which also allows assessing motor learning
abilities. Other neuromotor functions are assessed by grip strength
measurements and detailed automated gait analysis. Grip Strength
measures maximal muscle strength of forelimbs and hind limbs on an
automated grip strength meter. The test can indicate neuromuscular
abnormalities. Gait Analysis is based on a fully automated analysis of
video records of animal foot prints. Gait analysis provides not only
information about motor coordination but also detailed kinematic
description of animal gait. The test can be used to studding models of
Amyotrophic Lateral Sclerosis (ALS), pain/arthritis, Parkinson’s
disease, muscle injury model or spinal cord injury. 
  SHIRPA  
 The purpose of SHIRPA assessments is to examine mice for obvious
physical characteristics, behaviours, and morphological
abnormalities. 
 After one hour of acclimatisation to the experimental room conditions
the mouse is placed into the transparent jar and basic observations of
animal activity and presence are recorded. Subsequently transfer
arousal, animal appearance, basic reflexes, and gait in arena are
estimated. Above the arena the mouse is examined for trunk curl, limb
grasping, and positional passivity. At the end of the evaluation the
animal is placed into the transparent tube. The tube is turned to
position mouse upside down for observing its ability of righting. 
  Grip Strength  
 Testing is performed in the room with light intensity of 110 lux.
Forelimbs and combined forelimbs and hindlimbs grip-strengths are
determined using an automated meter (Bioseb, Vitrolles, France). To
assess neuromuscular function, the mouse is pulled over a grid; test is
recorded in three trials. To account for potential effects of body mass
all grip strength measurements are normalized to body mass. 
   
 
 
  2.3.3  Sensorimotor
Gating 
  Acoustic Startle  
 Animal sensorimotor gating is tested in Startle Reflex system, which
allows to determine also Prepulse Inhibition (PPI). The acoustic Startle
Reflex test (SR) is an automated analysis of startle reflex in response
to acoustic stimuli. The test assesses sensorimotor processing by
measuring both afferent sensory information transmission and efferent
motor response (Yeomans and Frankland, 1995). The test can also serve as
a primary screen for hearing impairment. The lack of sufficient sensory
gating mechanism is thought to lead to an overflow of the sensory
stimulation and disintegration of the cognitive functions (Grillon et
al., 1994). SR paradigm is therefore largely used to assess the effects
of putative anti-psychotics and to explore possible genetic and
neurobiological mechanisms of psychosis-related behaviour. PPI is an
attenuation of startle response magnitude by pre-exposure to
non-startling stimulus. PPI provides operational measurement of
sensorimotor gating reflecting the ability of an animal to successfully
integrate relevant and inhibit irrelevant sensory information (Norris
and Blumenthal, 1996). Impaired PPI is observed in schizophrenia as well
as in other neuropsychiatric disorders (Swerdlow and Geyer, 1998).
Testing is automated and performed in three sound proofed cabinets;
protocol execution is controlled by software (Med Associates Inc., USA).
Animals are habituated to the holder for 10 minutes, and then test is
initiated. Four different pre-pulse magnitudes are used for pre-pulse
alone and with startle stimulus. Ten repetitions of startle, pre-pulse
and combination of pre-pulse with startle stimuli are conducted in
pseudorandom order. The amplitude of animal response to all stimuli is
automatically recorded and % of startle response decrease in PPI is
computed manually. 
   
 
 
  2.3.4  Cognitive
functions 
 Cued and Contextual Conditioning, Context Discrimination, Barnes
Maze, Novel Object Recognition (NOR), and IntelliCage are tests used in
our facility to evaluate animal learning and memory 
  Cued and Contextual Conditioning  
 Cued and Contextual Conditioning are based on classical Pavlovian
fear conditioning, an associative learning test routinely used to study
biological basis of fear, learning, and memory. Contextual, but not
cued, fear conditioning is regarded as hippocampus-dependent. Although
this statement is true in most of experimental designs, animals can
compensate for hippocampal damage (Frankland et al, 1998). To overcome
this limitation, we also employed Context Discrimination test where
intact hippocampus is critical (Bidenkapp and Rudy, 2007). 
 Fear Conditioning (FC) is one of the forms of passive learning that
can be used in many strains of mice and rats, even when more pronounced
motor deficits are problematic in other learning assays. The dependent
measure used in contextual and cued FC is a freezing, defined as
“absence of movement except for respiration”, which takes place
following pairing of an unconditioned stimulus (US) with a conditioned
stimulus (CS). Aversive stimulus, foot shock, is used for US, and
neutral cue, tone and a particular context, are used for CS. The
associative learning take place in a cage enclosed within a soundproof
cabinet (Ugo Basile, Gemonio, Italy). The cage is equipped with a
stainless steel rod floor for shock delivery (Fig.2). Testing is
performed in the room with light intensity of 110 lux. The acquisition
trial starts with a 3 min of adaptation period, after which mice are
presented with two pairings of the CS (20 sec of 4 kHz pure tone at 77
dB) and the US (a 1 sec, 0.3 mA constant current to the cage floor). The
US is presented with the termination of the CS. The intra-trial interval
(ITI) is 2 min. Mice remains in the chamber for 1 min after the last
shock delivery. Animals are tested for contextual memory 24 hours after
training. They are reintroduced to the training context and behaviour is
recorded for 3 min with no CS or US presentation. Delayed cue memory is
tested three hours later. Animals are placed in a novel context, with
different cage wall pattern and a smooth floor texture. The freezing
response to the CS is monitored for 2 min. for 2 min. 
   
 
  Fig. 2:  Freezing
detection of the animal in fear conditioning box
 
 
 
 
  2.4  Metabolism 
 The metabolism unit uses a multi-technique approach to build a
complex picture of the overall energy storage and expenditure in the
whole animal model. In humans, disturbances in energy balance result in
the development of obesity or anorexia nervosa which presents as weight
gain or weight loss respectively. Cancer cachexia describes a syndrome
of progressive weight loss, anorexia, and persistent erosion of host
body cell mass in response to a malignant growth. A decline in food
intake relative to energy expenditure (which may be increased, normal,
or decreased) is the fundamental physiologic derangement leading to
cancer-associated weight loss. The primary metabolic screen uses
indirect calorimetry, intraperitoneal glucose tolerance test (IPGTT) and
body composition analysis to analyse the overall energy expenditure,
glucose metabolism and energy storage of the model. This data can be
used to guide more in depth analysis at the molecular level to further
understand the complex mechanism pathways controlling and affecting
energy homeostasis. 
   
 
  2.4.1  Indirect
Calorimetry 
 The oxygen consumption, carbon dioxide production and heat production
are measured using the PhenoMaster system from TSE systems. Animals are
individually housed in their home cages (Sealsafe Plus/Green line;
Techniplast) with food and water available ad libitum. Food and water
intake are measured for the duration of the study as well as locomotor
activity (measured by infrared beam cuts). Animals are weighed &amp;
placed in individual cages. The animals are allowed to acclimatise in
the system for 24 hrs. During this time,measurements are recorded,
however the data obtained during this time is not used in the final
analysis (Fig. 1). At the end of the experiment, animals are weighed and
returned to their original home cage. 
 
  Fig. 1:  Calorimetry
workflow
 
   
 
 
  2.4.2  Intraperitoneal
glucose tolerance test (IPGTT) 
 The glucose tolerance test measures the clearance of an
intraperitoneally injected glucose load from the body. It is used to
detect disturbances in glucose metabolism that can be linked to human
conditions such as diabetes or metabolic syndrome. 
 Animals are transferred to clean cages and fasted overnight
(16-18hrs) with water available ad libitum. Animals are then weighed and
the fasted blood glucose levels is recorded (T=0) from whole blood taken
from the tail vein using a standard glucometer. 20% glucose solution (2g
of glucose/kg body mass) in PBS is by intraperitoneal (IP) injection.
The blood glucose level is measured 15, 30, 60 and 120 mins after the
administration of glucose. After the final measurement, animals are
returned to the original home cage and food and water available ad
libitum. 
 
 
  2.4.3  Body
composition 
  In vivo  body composition analysis is an important and
effective tool both in early and late adult phenotyping in IMPC to
detect genes involved in energy balance regulation and the development
of lean or obese phenotypes when fed a chow diet. We use an equipment
based on quantitative Nuclear Magnetic Resonance (qNMR), the Minispec
LF90ii (Bruker). This equipment has been specially designed to measure
the body composition of rats and mice, using a magnetic field strength
of 0.47 Teslas. qNMR allows measuring body composition quickly,
non-invasively, and without anesthesia. Implementing qNMR significantly
reduces experimental burdens improving animal welfare. qNMR is a
high-throughput method and suitable for longitudinal studies. 
 Measurements are performed on each mouse separately. Before the
measurement, we weigh the animal’s body weight. The mice are placed in a
red plastic restrainer or holder, introduced into the magnet tunnel on a
horizontally oriented axis, and the equipment measure the body
composition in less than 4 minutes at room conditions. After the
measurement, the animals are returned to their original cages. With this
procedure, we measure lean mass, fat mass and free fluid mass in a
quick, easy and less stressful way for the animal. 
 
  Fig. 1:  Calorimetry
workflow
 
 
 
 
  2.5  Cardiovascular
function 
 Echocardiography and electrocardiography (ECG) are the most
frequently used techniques for diagnosing cardiovascular diseases. These
methods are non-invasive and the combination of both techniques allows a
first characterization of cardiovascular physiology and function of
genetically modified mice. 
   
 
  2.5.1 
Echocardiography 
 Murine echocardiography is a “gold standard” method to assess
cardiovascular structure and function (systolic and diastolic) in
anaesthetized mice that has been adopted from human system (Ram et al,
2011). In order to visualize the small mouse heart and record the rapid
heart rates, the cardiac ultrasound imaging is acquired using the Vevo
2100 Imaging System (VisualSonics, Inc.) with a 30 MHz transducer
(MS400) operating at a frequency that provides highly reliable and
reproducible image quality. Echocardiography is performed on
anaesthetised (isoflurane) mice and during imaging, the concentration of
anaesthesia is controlled to maintain a heart rate of 450-500 beats/min.
The primary echocardiographic screen includes two-dimensional (2D)
B-mode (“brightness” mode) imaging view of left ventricle (LV) along the
parasternal long axis (PLAX) and M-mode (“motion” mode) image of the
heart in short-axis view (SAX) for accurate linear measurements of left
ventricular internal dimensions (Collins et al., 2009, Moreth et al.,
2014, Stypmann et al, 2009). 
 
  Fig. 1:  Equipment of the
Cardiovascular Phenotyping Unit. Upper panel – Vevo2100 Ultrasound
Imaging System (VisualSonics, Inc.); bottom panel – ECGenie system
(Mouse Specifics, Inc.)
 
    
 A proper cardiac B-mode view allows visualization of the left atrium
and ventricle, a small portion of the right ventricular wall and the
output of aorta. The apex and the beginning of ascending aorta of the
heart lay on the same horizontal line. M-mode echocardiography is
performed at the ventricular level at the papillary muscle level and
leads to a 1D high-resolution temporal course of the diameter changes of
the LV and the wall thickness of the anterior and posterior LV wall in
systole as well in diastole. All M-mode echocardiography measurements
follow the guidelines of the American Society of Echocardiography (Sahn
et al, 1978). The SAX M-mode images are used to measure left ventricular
end-diastolic internal diameter (LVEDD), left ventricular end-systolic
internal diameter (LVESD), and diastolic and systolic posterior wall
thickness (LVPW) in three consecutive beats. Fractional shortening (FS)
is calculated as FS%=[(LVEDD-LVESD)/LVEDD]x100. Ejection fraction (EF)
is calculated as EF%=100 x ((LVvolD-LVvolS)LVvolD) with
LVvol=((7.0/(2.4+LVID) x LVID3). The corrected left ventricular mass (LV
Mass Cor) is calculated as LV Mass Cor=0.8 (1.053 x ((LVIDD + LVPWD +
IVSD)3 - LVIDD3)). The Stroke volume (SV) is the volume of blood pumped
from one ventricle of the heart with each beat. The Stroke volume of the
LV is obtained by subtracting end-systolic volume (ESV) from
end-diastolic volume (EDV). The cardiac output (CO) is the volume of
blood pumped by the heart per minute. In addition, heart rate and
respiratory rate are calculated by measuring three systolic intervals,
respectively three respiratory intervals. 
 
  Fig. 2:  Representative
B-mode echocardiographic image of an adult mouse heart, PLAX view (a)
and SAX view (b). M-mode image of the left ventricle at level of
papillary muscles (c)
 
   
 
 
  2.5.2 
Electrocardiography 
 Genetically modified mice with a cardiovascular phenotype can be
characterized by recording changes of the rhythm of the heart, these
include slowing (bradycardia) or quickening (tachycardia) of the heart
rate, heart rate variability and/or alterations in the length of the
interval of sinus waves that are reflected in electrocardiogram (ECG)
traces. ECG reflects the electrical activity of the heart and is
comprised of P, Q, R, S, and T waves. The P wave is produced by atrial
depolarization, the QRS pulses represent ventricular depolarization, and
the T wave represents ventricular repolarization (Goldbarg et al.,
1968). Many studies have been conducted to examine the patterns of
activation and repolarization in the mouse heart and their relation to
the electrical cardiac activity (for a review see Boukens et al., 2014).
In our facility, the murine ECG is recorded in conscious mice through
the animal`s paws using the ECGenie system (Mouse Specifics, Inc.). The
size and spacing of disposable footplate electrodes facilitate contact
between the electrodes and the paws to provide Einthoven lead II ECG
(Boukens et al., 2014). For each animal, heart depolarization and
repolarization intervals and amplitudes are evaluated from continuous
ECG recording after 5-min acclimation period. Only runs where at least
15 ECG beats could be included in the analysis are chosen. 
 Data are analysed using standard protocols for ECG signal analysis by
e-MouseTM (Mouse Specifics, Inc.). The software uses a peak detection
algorithm to find the peak of the R-waves and to calculate heart rate
(HR). The software plots its interpretation of P, Q, R, S, and T for
each beat so that unfiltered noise or motion artefacts are rejected.
This is followed by calculations of the mean of the ECG time intervals
for each set of waveforms. The corrected QT interval (QTc) is calculated
by dividing the QT interval by the square root of the preceding RR
interval. QT dispersion is measured as inter-lead variability of QT
intervals. The QTc dispersion was calculated as the rate corrected QT
dispersion. 
 
  Fig. 3: 
Schematic representation of the murine ECG
 
 
 
 
  2.6  Lung function 
 For an optimal gas-exchange in the lungs the air should be able to
easily flow in and out of the lungs. Key parameters for this are the
resistance the air encounters and the elasticity of the lung tissue
which allows the lungs to empty themselves efficiently during the
exhalation phase. 
 Both these parameters can be measured by the ‘Forced Oscillation’
technique in which pressure waves, called perturbations, are applied to
the lungs and the response to these pressure waves is recorded and
analyzed. To perform these perturbation mice are first anaesthetized
with ketamine and xylazine and intubated after which they are attached
to a computer controlled piston ventilator, the Flexivent FX from
Scireq. Mice are ventilated at 190 breaths per minute, which allows for
an optimal oxygenation and fast recovery of the oxygen saturation after
perturbations. 
 For the analysis of the response curves of the perturbations, a
mathematical model of the lungs is used. There are mainly 2 models and
also 2 types of perturbation. 
 In the first model, called the single compartment model, the lung is
modelled as a balloon, which confers the elasticity and represents the
lung tissue, attached to a tube, which confers the resistance to the
airflow and represents the airways. This model is simple but very robust
and the perturbation applied is relatively simple, it just consists of a
sinusoidal wave at a frequency of 150 cycles per minute. This
perturbation is called the SnapShot-150, which is abbreviated SN in
figures. From this 2 variables are calculated, the airway resistance (R)
and the elastance (E) of the lungs. Sometimes the elasticity parameter
is expressed as compliance (C), which is the reciprocal of the elastance
(C = 1/E). 
 The second model, the complex phase model, is more complex and it
models the airway resistance (R) and the resistance in the lung tissue,
called tissue damping (G), separately. This is possible due to a
difference in frequency response of these 2 structures. For the
elasticity parameters, the component of the airways is negligible and
thus only 1 elasticity parameter is calculated, the tissue elastance
(H). As the difference between airways and lung-tissue is the frequency
response of these tissues, the perturbation is a multi-frequency
perturbation. We use 2 different ones in our primary screen. The first
one takes 3 seconds, which gives a less precise estimate, but which can
be performed in mice with severely compromised lung-functions as the
period without fresh air is only 3 seconds. This manoeuver is called
Quick-prime 3, abbreviated as QP3 in figures. The second manoeuver, the
Prime-8, abbreviated P8, last as its name suggests, 8 seconds. For a
mouse breathing normally at about 180 to 200 breaths per minute this is
relatively long and could thus lead to oxygen desaturation in animals
with severely compromised lung function. In this case the animal will
start spontaneous breathing movements which will compromise the
measurement. This estimation of the lung function parameters is more
accurate as more data points are available for model fitting. 
 There is an additional manoeuver which is performed which is called
‘Deep inflation’ and is a forced deep inhalation. This serves 2
purposes, firstly, to measure the lung capacity of the animal and
secondly to open up the lungs completely so that all parts of the lung
are recruited in the consecutive manoeuvers. 
 All manoeuvers are executed in an automated script 5 times. If some
measurements are invalid during script execution, they are repeated
until 5 valid measures are obtained, with the exception of the prime-8
measurements. These measurements are not reattempted after the script if
obvious oxygen desaturation occurs, as it would be harmful for the
animal and would not lead to good quality data. 
 
 
  2.7  Bioimaging 
 The bioimaging unit is dedicated to comprehensive morphological and
functional characterization of animal models by whole-body imaging
systems in vivo and ex vivo. The primary tests cover morphological
analysis of the entire skeleton and quantitative analysis of mineral
content. Data for 3D body composition analysis is also acquired during
the same scan (Judex et al., 2010). Secondary tests and supplemental
approaches are established for more detailed and comprehensive
characterization of skeleton related phenotypes: 
 
 detail ex-vivo bone structure imaging with quantification of bone
porosity and mineral content (osteoporosis or arthritis models), or
in-vivo of selected skeleton area (e.g. femur or autopodium) 
 segmentation and volumetric analysis of individual bones – for
craniofacial phenotypes) 
 enamel and dentin mineralization imaging (Wald et al., 2017) 
 customized optical imaging including fluorescence protein detection,
fluorescence probes for inflammation, tumour development or bone
remodeling 
 skeletal preparation and alizarin/alcian staining for bone/cartilage
visualization. 
 
 Animals are anaesthetised using 20% zoletil solution. Once
anaesthetized, animals are weighed and body length is recorded with dial
caliper. Animals are placed into the animal restrainer and the scan is
initiated using the SkyScan software. 
  microCT (Skyscan 1176, Bruker)   in vivo 
scanner provides sensitive and high resolution 3D imaging based on X-ray
projections with voxel size 9 – 35μm. Our imaging set up is suitable
either for  in vivo  or  ex vivo  imaging of large
specimen up to size of rat.  In vivo  scans can provide
comprehensive 3D visualization of bones and other mineralized tissues
like teeth, but also quantification of total body fat mass and lean mass
(alternative to DEXA analysis) and their changes in time. 
  Ex vivo  scanning mode can be used for higher resolution
imaging of large body parts or use of contrast agents for imaging soft
tissues (liver, kidney, heart, neuronal tissue) or imaging fixed embryos
(E9.5 – E18.5) with use of appropriate contrast agents (Iodine, PTA).
For complete microCT analysis post-processing of projection data is
necessary, especially 3D reconstruction and further segmentation for
visualization of morphological phenotypes and quantification of selected
organs. 
 The system calibrates automatically after turning on after 3 or more
days without usage, plus flat field correction is processed whenever the
scanning parameters are changed. 
  Optical whole body imaging system (Xtreme, Bruker) 
includes highly sensitive camera for detection of emitted photons based
on enzymatic activity or fluorescence. This approach is suitable for in
vivo imaging with emphasis on noninvasive imaging setup for longitudinal
monitoring of studied process. This imaging modality is established for
inflammation, tumour progression, metastasis or cell homing experiments.
We have successfully published our imaging protocol in the study of
genetic regulation of DSS colitis (Brauer et al., 2015). 
  Software tool box:  NRecon, DataViewer, ITK-snap,
3D-slicer, CTvox, CTanalyser, CTvolume,Imaris, microCT (Skyscan 1176,
Bruker), Optical whole body imaging system (Xtreme, Bruker) 
 
 
  2.8  Auditory Brain Stem
Response (Hearing) 
 Auditory Brainstem Response (ABR) is an acoustically evoked
electrical potential recorded by needle electrodes placed subdermally on
the top of animal’s head. It represents a sum of activity of brainstem
neurons responsible for sound processing. Typical ABR evoked by a short
sound of supra-threshold intensity is a waveform composed of several
successive peaks that appear between 2 – 6 ms after the sound onset.
Their amplitudes vary in the range of hundreds of nanovolts and
correlate with the stimulus intensity. The latencies of individual ABR
peaks reflect successive activation of the auditory nerve, the cochlear
nucleus, the superior olivary complex and the inferior colliculus, which
comprise the brainstem portion of the auditory pathway (for review see
Biacabe et al., 2001; Møller, 2006). 
 The ABR recording protocol follows guidelines as described by Ingham
et al., 2011. The mouse is anaesthetized by i.p. injection of Zoletil
(mixture of tiletamine and zolazepam) and placed on a heating pad inside
an 6 m 3  anechoic soundproof chamber. Three subdermal needle
electrodes are positioned as follows: active electrode on the vertex,
reference electrode behind the left ear and ground electrode behind the
right ear, see Fig. 1. The system for sound production and biosignal
recording is based on TDT workstation RZ6 (Tucker-Davies Technologies,
USA). Acoustic stimuli are delivered by a high quality ribbon tweeter
RAAL 140-15D (RAAL Advanced Loudspeakers, Serbia) placed approximately
40 cm in front of the mouse. Each stimulus is played repeatedly and the
averaged ABR signal is recorded. Initially, the ABRs are evoked by
broadband stimuli (“clicks”, i.e. 100 microseconds lasting rectangular
pulses) followed by bursts of pure tones (5 ms long, 1 ms rise/fall
times) of 306, 2412, 1818, 12 24 and 630 kHz frequency respectively.
Stimulation always starts with the lowest sound intensity of 0 dB SPL
(sound pressure level) then the intensity is increased by 5 dB steps
until 85 dB SPL is reached. 
 
  Fig. 1:  mouse
under general anesthesia with subcutaneous electrodes for ABR recording.
Black wire - reference electrode, behind the left ear, green wire -
ground electrode behind the right ear, red wire - active electrode on
the vertex.
 
   
 The primary output of ABR analysis is detection of hearing thresholds
for the broadband stimulus (click) and for each frequency used. The
threshold is defined as the lowest intensity of sound which evoked
distinguishable response related to (time-locked to) the stimulus.
Statistical significance of the difference in thresholds between two
groups of animals is tested by two-way ANOVA with replication combined
with post-hoc tests. Other parameters such as latency, amplitude and
waveform shape may be examined in addition if it is of interest. 
 
  Fig. 2: 
Auditory Brainstem Response. The animal was stimulated by a pure tone of
24 kHz frequency, 5 ms duration and intensities ranging between 0 - 85
dB SPL, the stimulus onset is marked with a vertical arrow (Stim.). Each
curve represents an average of 600 repetitions. The lowest sound
intensity that evoked a response is referred to as the hearing threshold
for the respective frequency, marked with a horizontal arrow (Thr.).
Five typical peaks of ABR signal are labelled with Roman numerals I to
V. Note that with increasing intensity not only the peaks are higher but
they have shorter latency, too.
 
   
 
 
  2.9  Eye morphology 
 Eyes are a very important sense organ for human beings.
Unfortunately, many people suffer from serious ocular diseases like
cataracts, glaucoma or retinal disorders and their life quality is
strongly affected. Many genes are involved in a variety of ocular
disorders. As the genome of the mouse is well known, the mouse is a
suitable model for understanding of the biochemical, genetic and
physiological mechanisms of many hereditary degenerative ocular
disorders. 
 The vision screen uses non-invasive imaging devices to examine the
anterior segment (Pentacam) and retina of the eyes (OCT). The animals
are anaesthetised using 20% Zoletil and the pupils are dilated using eye
drops containing 0.5% Atropin. The mouse is placed on a platform of the
Pentacam and the centre of the pupil is orientated to the light-beam
slit of the Pentacam´s head. This device enables the measurement of many
parameters of the cornea and the lens (e.g. surface, form, opacity,
thickness and density) for each eye. All 25 scanned images from
different angles of the eye are carried out by a rotating Scheimpflug
Camera of the Pentacam they are analysed in order to assess any
abnormalities of the eye. 
 The optical coherent tomograph (OCT) scans, quantifies the reflection
of a light beam sending from the layers of the retina and composes
cross-sectional images of the retina. Each cross-section image of the
retina will be evaluated and a variety of parameters are measured
e.g. the thickness and the gross morphology of the retina, form and
position of the optical disc, the blood vessels and their pattern. 
 
  Fig. 1:  Representative
example of the retinal fundus with the optic disc and blood vessel
system (left) and a cross-sectional image of retina (right) in the wild
type mouse C57Bl/6NCrl.
 
   
 
 
  2.10  Biochemistry and
Hematology 
 Biochemical and haematology phenotyping is based on robust primary
screening developed under Eumorphia and EMPReSS, a European Mouse
Phenotyping Resource for Standardised Screens. Phenotyping investigation
includes also newest development in INFRAFRONTIER and IMPC consortia.
These tests comprise primary phenotyping pipeline and are carried out
according the standardised protocols as detailed in (IMPReSS). The
Biochemical and haematology unit currently analyses various blood
metabolites, ions, hormones, and enzymes of genetically modified mice
compared with age matched controls. Changes in these data can be linked
to metabolic and functional abnormalities of specific organs such as
liver, kidney, and gastrointestinal tract. 
  Blood Withdrawal and Storage  
 Blood samples are taken from isoflurane-anesthetized mice by
retro-bulbar sinus puncture with non-heparinized glass capillaries.
Samples are collected in two different types of coated tubes
(Lithium/Heparin (KABE cat # 078028) and EDTA (KABE cat # 078035). After
collection each sample is mixed by gentle inversion and then kept on RT
until centrifugation. Samples are centrifuged within 1 hour of
collection at 5000 X g, for 10 minutes at 8°C. Once separated from the
cells, plasma samples are refrigerated if analysis is delayed. If plasma
cannot be analyzed fresh, samples are stored at -20oC (short-term) or
-70oC (long-term) storage. Freeze/thaw cycles are avoided. 
 Plasma is analyzed on Beckman AU480 biochemical analyzer. All
calibration and quality control samples are within expiration period and
measured values fulfilled required parameters for each method used in
this report. 
  Hematology  
 Our hematology test requires 20 μl of whole blood in EDTA coated
tubes per sample. 20 μl of whole blood is diluted to final volume is 200
μl. Complete and differential blood cell counts were measured on
Mindray-BC-5000Vet 
 
 
  2.11  Immunology 
 As a integral part of the terminal screen, immunophenotyping involves
characterisation of particular immune cell populations in terms of their
cellularity and phenotype using multicolor flow cytometry. 
 The procedures are based on standard immunophenotyping protocols of
the Adult and Embryonic Phenotype Pipeline that has been agreed by the
research institutions involved  MPReSS
-International Mouse Phenotyping Resource of Standardised
Screens . 
 According to these guidelines, we use mouse spleenocytes for standard
immunophenotyping. However, we are able to analyze peripheral blood,
lymph nodes, thymi, bone marrow or peritoneal lavage samples as
well. 
   
 
  Fig. 1:  Gating strategy
to discriminate T cell populations using Panel A
 
   
 Routinelly, we utilize two panels to detect lymphoid and myeloid
cells. In addition, the spleen weight is an important indicator which is
also recorded. The procedure includes several quality control steps
(cell viability upon digestion, number of cells/sample after
acquisition). 
 For primary tests, we typically use three males and three females
knock out (KO) animals plus three male and three female wild type
controls (WT), plus optionally one or two control mice as internal
controls. Flow cytometry data are acquired using BD LSRFortessaTM SORP,
manualy analyzed in FlowJo software and statistically evaluated. 
   
 
  Fig. 2:  Gating strategy
to discriminate B cell and myeloid populations using Panel B
 
   
 
 
  2.12  Histopathology 
 The histopathology screen is dedicated for macroscopic and
microscopic analysis of pathological alterations occurring in mutant
mouse models. The unit is comparing gross morphology and microscopic
differences between wild-type and gene-engineered mouse models. A major
task is to screen for set of pathologies connected to specific genetic
status using the tools and techniques of the classical and molecular
pathology. 
 
  2.12.1  Gross Morphology
and Tissue Processing 
  Full mouse necropsy with organ isolation  
 A complete necropsy is performed to detect and record abnormal
macroscopic alterations in internal and external organs, record body,
spleen, liver, kidney (dx., sin.) and heart weights (according to IMPC
Heart Weight SOP) and tibia length. 
 Samples from IMPC ‘Tissue Collection List’ (minimal number is 2
mutant males and 2 mutant females) are fixed, trimmed, processed and
embedded in paraffin blocks. The femur is also prepared and blood smears
and bone marrow smears are also prepared. 
 Images of all abnormal findings are documented and saved in database.
All collected tissues are placed in a labelled jar containing a
sufficient volume of fixative (minimum of 10:1 fixative to tissue). All
abnormal findings are recorded according to our Centre-specific database
using the standardized IMPC Gross Pathology ontology. For long-term
storage, the fixed samples are stored in paraffin blocks. 
  Organ sampling and trimming  
 Individual organs are processed. Unless otherwise specified, organs
are processed according to the  Revised guides for organ sampling and
trimming in rats and mice , published in 2003 and 2004 by the
Registry of Industrial Toxicology Animal-data (RITA) and the North
American Control Animal Database (NACAD) groups (Exp Toxic Pathol 55:
91-106, Exp Tox Pathol 55: 413-431, and Exp Tox Pathol 55: 433-449). 
  Tissue processing and embedding  
 By using a state-of-the-art automated vacuum tissue processor (Leica
ASP6025), we process and paraffin-embed specimens with the highest
levels of reproducibility and quality. Where applicable, decalcification
to remove mineral from bone or other calcified tissues is performed
prior to processing to paraffin. Tissues are embedded in paraffin using
standard orientation according to IMPC Tissue Embedding and Block
Banking IMPC_BLK_001 SOP. 
 
 
  2.12.2  Sectioning and
Staining 
  Sectioning  
 Standard paraffin sections are cut using a Leica RM2255-FU microtome.
The standard thickness of sections is 2µm. 
  H&amp;E staining  
 The standard primary staining procedure is the hematoxylin and eosin
(H&amp;E) stain. The use of the Ventana Symphony automated staining
system ensures high throughput processing with consistent and
reproducible results. 
 
 
  2.12.3  Histopathological
examination 
 Histopathological examination is performed using the Carl Zeiss Axio
Imager.Z2 or Leica DM3000 microscope. All abnormal findings are recorded
in our Centre-specific database using the standardized IMPC
Histopatology ontology. 
     
 
 
 
 
  3  Results 
 
  3.1  Weight Curve - 4 week
to 16 week 
   
 
 
 
 STRAIN 
 GENDER 
 count 
 
 
 
 
 WT/WT 
 female 
 8 
 
 
 WT/WT 
 male 
 8 
 
 
 Δ HEL/WT 
 female 
 8 
 
 
 Δ HEL/WT 
 male 
 8 
 
 
 
   
   
 
 
 
 
 
 
 
 
 
 
  
 test 
 Difference 
 p_value 
 Significance 
 
 
 
 
 c2 
 WT/WT vs Δ HEL/WT 
 2.9151 
 0.0000 
 TRUE 
 
 
 c3 
 Female vs Male 
 -5.6632 
 0.0000 
 TRUE 
 
 
 c4 
 Female WT/WT vs Female Δ HEL/WT 
 1.8323 
 0.1636 
 FALSE 
 
 
 c5 
 Male WT/WT vs Male Δ HEL/WT 
 3.9979 
 0.0000 
 TRUE 
 
 
 c6 
 Female Δ HEL/WT vs Male Δ HEL/WT 
 -4.5804 
 0.0000 
 TRUE 
 
 
 
 
 
  3.2  Open field 
  Number of animals  
   
 
 
 
 STRAIN 
 GENDER 
 count 
 
 
 
 
 WT/WT 
 female 
 8 
 
 
 WT/WT 
 male 
 8 
 
 
 Δ HEL/WT 
 female 
 8 
 
 
 Δ HEL/WT 
 male 
 8 
 
 
 
   
 
  3.2.1  Center average
speed - total (cm/s) 
   
 
 
 
 
 
 
 
 
 
 
  
 test 
 Difference 
 p_value 
 Significance 
 
 
 
 
 c2 
 WT/WT vs Δ HEL/WT 
 0.550 
 0.9415 
 FALSE 
 
 
 c3 
 Female vs Male 
 -1.175 
 0.7910 
 FALSE 
 
 
 c4 
 Female WT/WT vs Female Δ HEL/WT 
 -0.725 
 0.9519 
 FALSE 
 
 
 c5 
 Male WT/WT vs Male Δ HEL/WT 
 1.825 
 0.5477 
 FALSE 
 
 
 c6 
 Female Δ HEL/WT vs Male Δ HEL/WT 
 0.100 
 0.9999 
 FALSE 
 
 
 
     
 
 
  3.2.2  Center distance
travelled - total (cm) 
   
 
 
 
 
 
 
 
 
 
 
  
 test 
 Difference 
 p_value 
 Significance 
 
 
 
 
 c2 
 WT/WT vs Δ HEL/WT 
 -185.0625 
 0.8086 
 FALSE 
 
 
 c3 
 Female vs Male 
 48.8875 
 0.9955 
 FALSE 
 
 
 c4 
 Female WT/WT vs Female Δ HEL/WT 
 -557.5625 
 0.2371 
 FALSE 
 
 
 c5 
 Male WT/WT vs Male Δ HEL/WT 
 187.4375 
 0.9188 
 FALSE 
 
 
 c6 
 Female Δ HEL/WT vs Male Δ HEL/WT 
 421.3875 
 0.4867 
 FALSE 
 
 
 
     
 
 
  3.2.3  Center permanence
time - total (s) 
   
 
 
 
 
 
 
 
 
 
 
  
 test 
 Difference 
 p_value 
 Significance 
 
 
 
 
 c2 
 WT/WT vs Δ HEL/WT 
 4.2012 
 0.9997 
 FALSE 
 
 
 c3 
 Female vs Male 
 35.1363 
 0.9049 
 FALSE 
 
 
 c4 
 Female WT/WT vs Female Δ HEL/WT 
 -6.0500 
 0.9997 
 FALSE 
 
 
 c5 
 Male WT/WT vs Male Δ HEL/WT 
 14.4524 
 0.9959 
 FALSE 
 
 
 c6 
 Female Δ HEL/WT vs Male Δ HEL/WT 
 45.3875 
 0.9116 
 FALSE 
 
 
 
     
 
 
  3.2.4  Center resting time
- total (s) 
   
 
 
 
 
 
 
 
 
 
 
  
 test 
 Difference 
 p_value 
 Significance 
 
 
 
 
 c2 
 WT/WT vs Δ HEL/WT 
 10.7177 
 0.9922 
 FALSE 
 
 
 c3 
 Female vs Male 
 34.0483 
 0.8754 
 FALSE 
 
 
 c4 
 Female WT/WT vs Female Δ HEL/WT 
 16.7891 
 0.9894 
 FALSE 
 
 
 c5 
 Male WT/WT vs Male Δ HEL/WT 
 4.6463 
 0.9998 
 FALSE 
 
 
 c6 
 Female Δ HEL/WT vs Male Δ HEL/WT 
 27.9769 
 0.9648 
 FALSE 
 
 
 
     
 
 
  3.2.5  Distance travelled
- total (cm) 
   
 
 
 
 
 
 
 
 
 
 
  
 test 
 Difference 
 p_value 
 Significance 
 
 
 
 
 c2 
 WT/WT vs Δ HEL/WT 
 -88.2938 
 0.9973 
 FALSE 
 
 
 c3 
 Female vs Male 
 73.5188 
 0.9985 
 FALSE 
 
 
 c4 
 Female WT/WT vs Female Δ HEL/WT 
 -981.8875 
 0.4175 
 FALSE 
 
 
 c5 
 Male WT/WT vs Male Δ HEL/WT 
 805.3000 
 0.5873 
 FALSE 
 
 
 c6 
 Female Δ HEL/WT vs Male Δ HEL/WT 
 967.1125 
 0.4336 
 FALSE 
 
 
 
     
 
 
  3.2.6  Number of center
entries - total 
   
 
 
 
 
 
 
 
 
 
 
  
 test 
 Difference 
 p_value 
 Significance 
 
 
 
 
 c2 
 WT/WT vs Δ HEL/WT 
 -13.5625 
 0.2289 
 FALSE 
 
 
 c3 
 Female vs Male 
 -0.1875 
 1.0000 
 FALSE 
 
 
 c4 
 Female WT/WT vs Female Δ HEL/WT 
 -28.5000 
 0.0274 
 TRUE 
 
 
 c5 
 Male WT/WT vs Male Δ HEL/WT 
 1.3750 
 0.9991 
 FALSE 
 
 
 c6 
 Female Δ HEL/WT vs Male Δ HEL/WT 
 14.7500 
 0.4569 
 FALSE 
 
 
 
     
 
 
  3.2.7  Periphery average
speed - total (cm/s) 
   
 
 
 
 
 
 
 
 
 
 
  
 test 
 Difference 
 p_value 
 Significance 
 
 
 
 
 c2 
 WT/WT vs Δ HEL/WT 
 0.1938 
 0.9064 
 FALSE 
 
 
 c3 
 Female vs Male 
 0.0812 
 0.9924 
 FALSE 
 
 
 c4 
 Female WT/WT vs Female Δ HEL/WT 
 -0.2875 
 0.8937 
 FALSE 
 
 
 c5 
 Male WT/WT vs Male Δ HEL/WT 
 0.6750 
 0.3527 
 FALSE 
 
 
 c6 
 Female Δ HEL/WT vs Male Δ HEL/WT 
 0.5625 
 0.5219 
 FALSE 
 
 
 
     
 
 
  3.2.8  Periphery distance
travelled - total (cm) 
   
 
 
 
 
 
 
 
 
 
 
  
 test 
 Difference 
 p_value 
 Significance 
 
 
 
 
 c2 
 WT/WT vs Δ HEL/WT 
 159.2750 
 0.9553 
 FALSE 
 
 
 c3 
 Female vs Male 
 -37.8875 
 0.9993 
 FALSE 
 
 
 c4 
 Female WT/WT vs Female Δ HEL/WT 
 -299.3250 
 0.9029 
 FALSE 
 
 
 c5 
 Male WT/WT vs Male Δ HEL/WT 
 617.8750 
 0.4972 
 FALSE 
 
 
 c6 
 Female Δ HEL/WT vs Male Δ HEL/WT 
 420.7125 
 0.7729 
 FALSE 
 
 
 
     
 
 
  3.2.9  Periphery
permanence time - total (s) 
   
 
 
 
 
 
 
 
 
 
 
  
 test 
 Difference 
 p_value 
 Significance 
 
 
 
 
 c2 
 WT/WT vs Δ HEL/WT 
 -4.1687 
 0.9997 
 FALSE 
 
 
 c3 
 Female vs Male 
 -35.2062 
 0.9045 
 FALSE 
 
 
 c4 
 Female WT/WT vs Female Δ HEL/WT 
 6.0375 
 0.9997 
 FALSE 
 
 
 c5 
 Male WT/WT vs Male Δ HEL/WT 
 -14.3750 
 0.9960 
 FALSE 
 
 
 c6 
 Female Δ HEL/WT vs Male Δ HEL/WT 
 -45.4125 
 0.9116 
 FALSE 
 
 
 
     
 
 
  3.2.10  Periphery resting
time - total (s) 
   
 
 
 
 
 
 
 
 
 
 
  
 test 
 Difference 
 p_value 
 Significance 
 
 
 
 
 c2 
 WT/WT vs Δ HEL/WT 
 -5.6434 
 0.9991 
 FALSE 
 
 
 c3 
 Female vs Male 
 -31.1632 
 0.9145 
 FALSE 
 
 
 c4 
 Female WT/WT vs Female Δ HEL/WT 
 22.5107 
 0.9817 
 FALSE 
 
 
 c5 
 Male WT/WT vs Male Δ HEL/WT 
 -33.7975 
 0.9421 
 FALSE 
 
 
 c6 
 Female Δ HEL/WT vs Male Δ HEL/WT 
 -59.3173 
 0.7895 
 FALSE 
 
 
 
     
 
 
  3.2.11  Whole arena
average speed - total (cm/s) 
   
 
 
 
 
 
 
 
 
 
 
  
 test 
 Difference 
 p_value 
 Significance 
 
 
 
 
 c2 
 WT/WT vs Δ HEL/WT 
 -0.0938 
 0.9945 
 FALSE 
 
 
 c3 
 Female vs Male 
 0.0562 
 0.9989 
 FALSE 
 
 
 c4 
 Female WT/WT vs Female Δ HEL/WT 
 -0.8250 
 0.4159 
 FALSE 
 
 
 c5 
 Male WT/WT vs Male Δ HEL/WT 
 0.6375 
 0.6319 
 FALSE 
 
 
 c6 
 Female Δ HEL/WT vs Male Δ HEL/WT 
 0.7875 
 0.4643 
 FALSE 
 
 
 
     
 
 
  3.2.12  Whole arena
resting time - total (s) 
   
 
 
 
 
 
 
 
 
 
 
  
 test 
 Difference 
 p_value 
 Significance 
 
 
 
 
 c2 
 WT/WT vs Δ HEL/WT 
 4.50 
 0.9937 
 FALSE 
 
 
 c3 
 Female vs Male 
 3.15 
 0.9979 
 FALSE 
 
 
 c4 
 Female WT/WT vs Female Δ HEL/WT 
 37.95 
 0.4112 
 FALSE 
 
 
 c5 
 Male WT/WT vs Male Δ HEL/WT 
 -28.95 
 0.6374 
 FALSE 
 
 
 c6 
 Female Δ HEL/WT vs Male Δ HEL/WT 
 -30.30 
 0.6059 
 FALSE 
 
 
 
     
 
 
  3.2.13  Center average
speed 
   
 
 
 
 
 
 
 
 
 
 
  
 test 
 Difference 
 p_value 
 Significance 
 
 
 
 
 c2 
 WT/WT vs Δ HEL/WT 
 1.1740 
 0.5632 
 FALSE 
 
 
 c3 
 Female vs Male 
 -0.8568 
 0.9938 
 FALSE 
 
 
 c4 
 Female WT/WT vs Female Δ HEL/WT 
 -0.1420 
 0.9995 
 FALSE 
 
 
 c5 
 Male WT/WT vs Male Δ HEL/WT 
 2.4900 
 0.2204 
 FALSE 
 
 
 c6 
 Female Δ HEL/WT vs Male Δ HEL/WT 
 0.4591 
 0.9991 
 FALSE 
 
 
 
    
 
 
  3.2.14  Center distance
travelled 
   
 
 
 
 
 
 
 
 
 
 
  
 test 
 Difference 
 p_value 
 Significance 
 
 
 
 
 c2 
 WT/WT vs Δ HEL/WT 
 -29.8241 
 0.9348 
 FALSE 
 
 
 c3 
 Female vs Male 
 23.3947 
 0.9982 
 FALSE 
 
 
 c4 
 Female WT/WT vs Female Δ HEL/WT 
 -128.7124 
 0.2832 
 FALSE 
 
 
 c5 
 Male WT/WT vs Male Δ HEL/WT 
 69.0643 
 0.7694 
 FALSE 
 
 
 c6 
 Female Δ HEL/WT vs Male Δ HEL/WT 
 122.2830 
 0.8353 
 FALSE 
 
 
 
    
 
 
  3.2.15  Center permanence
time 
   
 
 
 
 
 
 
 
 
 
 
  
 test 
 Difference 
 p_value 
 Significance 
 
 
 
 
 c2 
 WT/WT vs Δ HEL/WT 
 0.8989 
 0.9997 
 FALSE 
 
 
 c3 
 Female vs Male 
 12.6685 
 0.9830 
 FALSE 
 
 
 c4 
 Female WT/WT vs Female Δ HEL/WT 
 -8.5481 
 0.9188 
 FALSE 
 
 
 c5 
 Male WT/WT vs Male Δ HEL/WT 
 10.3459 
 0.8660 
 FALSE 
 
 
 c6 
 Female Δ HEL/WT vs Male Δ HEL/WT 
 22.1155 
 0.9262 
 FALSE 
 
 
 
    
 
 
  3.2.16  Center resting
time 
   
 
 
 
 
 
 
 
 
 
 
  
 test 
 Difference 
 p_value 
 Significance 
 
 
 
 
 c2 
 WT/WT vs Δ HEL/WT 
 -1.1290 
 0.9985 
 FALSE 
 
 
 c3 
 Female vs Male 
 11.0548 
 0.9983 
 FALSE 
 
 
 c4 
 Female WT/WT vs Female Δ HEL/WT 
 -7.2540 
 0.8842 
 FALSE 
 
 
 c5 
 Male WT/WT vs Male Δ HEL/WT 
 4.9959 
 0.9579 
 FALSE 
 
 
 c6 
 Female Δ HEL/WT vs Male Δ HEL/WT 
 17.1798 
 0.9938 
 FALSE 
 
 
 
    
 
 
  3.2.17  Distance
travelled 
   
 
 
 
 
 
 
 
 
 
 
  
 test 
 Difference 
 p_value 
 Significance 
 
 
 
 
 c2 
 WT/WT vs Δ HEL/WT 
 10.8654 
 0.9996 
 FALSE 
 
 
 c3 
 Female vs Male 
 9.6069 
 1.0000 
 FALSE 
 
 
 c4 
 Female WT/WT vs Female Δ HEL/WT 
 -203.7555 
 0.5502 
 FALSE 
 
 
 c5 
 Male WT/WT vs Male Δ HEL/WT 
 225.4862 
 0.4631 
 FALSE 
 
 
 c6 
 Female Δ HEL/WT vs Male Δ HEL/WT 
 224.2278 
 0.9339 
 FALSE 
 
 
 
    
 
 
  3.2.18  Number of center
entries 
   
 
 
 
 
 
 
 
 
 
 
  
 test 
 Difference 
 p_value 
 Significance 
 
 
 
 
 c2 
 WT/WT vs Δ HEL/WT 
 -2.6436 
 0.4218 
 FALSE 
 
 
 c3 
 Female vs Male 
 0.3762 
 0.9999 
 FALSE 
 
 
 c4 
 Female WT/WT vs Female Δ HEL/WT 
 -6.3519 
 0.0525 
 FALSE 
 
 
 c5 
 Male WT/WT vs Male Δ HEL/WT 
 1.0646 
 0.9717 
 FALSE 
 
 
 c6 
 Female Δ HEL/WT vs Male Δ HEL/WT 
 4.0845 
 0.9064 
 FALSE 
 
 
 
    
 
 
  3.2.19  Periphery average
speed 
   
 
 
 
 
 
 
 
 
 
 
  
 test 
 Difference 
 p_value 
 Significance 
 
 
 
 
 c2 
 WT/WT vs Δ HEL/WT 
 0.2183 
 0.8657 
 FALSE 
 
 
 c3 
 Female vs Male 
 0.0799 
 0.9999 
 FALSE 
 
 
 c4 
 Female WT/WT vs Female Δ HEL/WT 
 -0.2622 
 0.9125 
 FALSE 
 
 
 c5 
 Male WT/WT vs Male Δ HEL/WT 
 0.6988 
 0.3049 
 FALSE 
 
 
 c6 
 Female Δ HEL/WT vs Male Δ HEL/WT 
 0.5604 
 0.9597 
 FALSE 
 
 
 
    
 
 
  3.2.20  Periphery distance
travelled 
   
 
 
 
 
 
 
 
 
 
 
  
 test 
 Difference 
 p_value 
 Significance 
 
 
 
 
 c2 
 WT/WT vs Δ HEL/WT 
 44.2225 
 0.9327 
 FALSE 
 
 
 c3 
 Female vs Male 
 -16.6322 
 0.9999 
 FALSE 
 
 
 c4 
 Female WT/WT vs Female Δ HEL/WT 
 -73.5711 
 0.8959 
 FALSE 
 
 
 c5 
 Male WT/WT vs Male Δ HEL/WT 
 162.0160 
 0.4172 
 FALSE 
 
 
 c6 
 Female Δ HEL/WT vs Male Δ HEL/WT 
 101.1614 
 0.9773 
 FALSE 
 
 
 
    
 
 
  3.2.21  Periphery
permanence time 
   
 
 
 
 
 
 
 
 
 
 
  
 test 
 Difference 
 p_value 
 Significance 
 
 
 
 
 c2 
 WT/WT vs Δ HEL/WT 
 -0.9067 
 0.9997 
 FALSE 
 
 
 c3 
 Female vs Male 
 -12.6778 
 0.7858 
 FALSE 
 
 
 c4 
 Female WT/WT vs Female Δ HEL/WT 
 8.5407 
 0.9216 
 FALSE 
 
 
 c5 
 Male WT/WT vs Male Δ HEL/WT 
 -10.3541 
 0.8697 
 FALSE 
 
 
 c6 
 Female Δ HEL/WT vs Male Δ HEL/WT 
 -22.1253 
 0.5451 
 FALSE 
 
 
 
    
 
 
  3.2.22  Periphery resting
time 
   
 
 
 
 
 
 
 
 
 
 
  
 test 
 Difference 
 p_value 
 Significance 
 
 
 
 
 c2 
 WT/WT vs Δ HEL/WT 
 -2.1205 
 0.9965 
 FALSE 
 
 
 c3 
 Female vs Male 
 -9.6907 
 0.9004 
 FALSE 
 
 
 c4 
 Female WT/WT vs Female Δ HEL/WT 
 9.5286 
 0.9053 
 FALSE 
 
 
 c5 
 Male WT/WT vs Male Δ HEL/WT 
 -13.7696 
 0.7620 
 FALSE 
 
 
 c6 
 Female Δ HEL/WT vs Male Δ HEL/WT 
 -21.3397 
 0.6040 
 FALSE 
 
 
 
    
 
 
  3.2.23  Whole arena
average speed 
   
 
 
 
 
 
 
 
 
 
 
  
 test 
 Difference 
 p_value 
 Significance 
 
 
 
 
 c2 
 WT/WT vs Δ HEL/WT 
 0.0275 
 0.9998 
 FALSE 
 
 
 c3 
 Female vs Male 
 0.0210 
 1.0000 
 FALSE 
 
 
 c4 
 Female WT/WT vs Female Δ HEL/WT 
 -0.6865 
 0.5463 
 FALSE 
 
 
 c5 
 Male WT/WT vs Male Δ HEL/WT 
 0.7416 
 0.4803 
 FALSE 
 
 
 c6 
 Female Δ HEL/WT vs Male Δ HEL/WT 
 0.7351 
 0.7923 
 FALSE 
 
 
 
    
 
 
  3.2.24  Whole arena
resting time 
   
 
 
 
 
 
 
 
 
 
 
  
 test 
 Difference 
 p_value 
 Significance 
 
 
 
 
 c2 
 WT/WT vs Δ HEL/WT 
 1.6251 
 0.9814 
 FALSE 
 
 
 c3 
 Female vs Male 
 0.8029 
 0.9999 
 FALSE 
 
 
 c4 
 Female WT/WT vs Female Δ HEL/WT 
 9.9993 
 0.3619 
 FALSE 
 
 
 c5 
 Male WT/WT vs Male Δ HEL/WT 
 -6.7491 
 0.6849 
 FALSE 
 
 
 c6 
 Female Δ HEL/WT vs Male Δ HEL/WT 
 -7.5713 
 0.9499 
 FALSE 
 
 
 
    
 
 
  3.2.25  Latency to center
entry (s) 
   
 
 
 
 
 
 
 
 
 
 
  
 test 
 Difference 
 p_value 
 Significance 
 
 
 
 
 c2 
 WT/WT vs Δ HEL/WT 
 32.4563 
 0.1763 
 FALSE 
 
 
 c3 
 Female vs Male 
 17.7312 
 0.8025 
 FALSE 
 
 
 c4 
 Female WT/WT vs Female Δ HEL/WT 
 52.5375 
 0.0944 
 FALSE 
 
 
 c5 
 Male WT/WT vs Male Δ HEL/WT 
 12.3750 
 0.9446 
 FALSE 
 
 
 c6 
 Female Δ HEL/WT vs Male Δ HEL/WT 
 -2.3500 
 0.9997 
 FALSE 
 
 
 
      
 
 
  3.2.26  Percentage center
time - total (%) 
   
 
 
 
 
 
 
 
 
 
 
  
 test 
 Difference 
 p_value 
 Significance 
 
 
 
 
 c2 
 WT/WT vs Δ HEL/WT 
 0.3458 
 0.9997 
 FALSE 
 
 
 c3 
 Female vs Male 
 2.9333 
 0.8676 
 FALSE 
 
 
 c4 
 Female WT/WT vs Female Δ HEL/WT 
 -0.5031 
 0.9997 
 FALSE 
 
 
 c5 
 Male WT/WT vs Male Δ HEL/WT 
 1.1948 
 0.9960 
 FALSE 
 
 
 c6 
 Female Δ HEL/WT vs Male Δ HEL/WT 
 3.7823 
 0.8945 
 FALSE 
 
 
 
      
 
 
 
  3.3  Shirpa and
Dismorphology 
  Number of animals  
   
 
 
 
 STRAIN 
 GENDER 
 count 
 
 
 
 
 WT/WT 
 female 
 8 
 
 
 WT/WT 
 male 
 8 
 
 
 Δ HEL/WT 
 female 
 8 
 
 
 Δ HEL/WT 
 male 
 8 
 
 
 
   
 
  3.3.1  Activity (body
position) 
    
 
 
  3.3.2  Aggression 
    
 
 
  3.3.3  Coat - color -
abdomen 
    
 
 
  3.3.4  Coat - color -
back 
    
 
 
  3.3.5  Coat - color -
head 
    
 
 
  3.3.6  Coat - color
pattern - abdomen 
    
 
 
  3.3.7  Coat - color
pattern - back 
    
 
 
  3.3.8  Coat - color
pattern - head 
    
 
 
  3.3.9  Coat - hair
distribution - abdomen 
    
 
 
  3.3.10  Coat - hair
distribution - back 
    
 
 
  3.3.11  Coat - hair
distribution - head 
    
 
 
  3.3.12  Coat - hair
texture_appearance - abdomen 
    
 
 
  3.3.13  Coat - hair
texture_appearance - back 
    
 
 
  3.3.14  Coat - hair
texture_appearance - head 
    
 
 
  3.3.15  Contact
righting 
    
 
 
  3.3.16  Ears 
    
 
 
  3.3.17  Forelimb digit -
number 
    
 
 
  3.3.18  Forelimb digit -
shape 
    
 
 
  3.3.19  Forelimb digit -
size 
    
 
 
  3.3.20  Forelimb nail -
length 
    
 
 
  3.3.21  Forelimb nail -
number 
    
 
 
  3.3.22  Forelimb nail -
shape 
    
 
 
  3.3.23  Forelimbs -
position 
    
 
 
  3.3.24  Forelimbs -
shape 
    
 
 
  3.3.25  Forelimbs -
size 
    
 
 
  3.3.26  Forepaw -
shape 
    
 
 
  3.3.27  Forepaw -
size 
    
 
 
  3.3.28  Gait 
    
 
 
  3.3.29  Genitalia -
morphology 
    
 
 
  3.3.30  Genitalia -
presence 
    
 
 
  3.3.31  Genitalia -
size 
    
 
 
  3.3.32  Head bobbing 
    
 
 
  3.3.33  Head
morphology 
    
 
 
  3.3.34  Head size 
    
 
 
  3.3.35  Hindlimb digit -
number 
    
 
 
  3.3.36  Hindlimb digit -
shape 
    
 
 
  3.3.37  Hindlimb digit -
size 
    
 
 
  3.3.38  Hindlimb nail -
length 
    
 
 
  3.3.39  Hindlimb nail -
number 
    
 
 
  3.3.40  Hindlimb nail -
shape 
    
 
 
  3.3.41  Hindlimbs -
position 
    
 
 
  3.3.42  Hindlimbs -
shape 
    
 
 
  3.3.43  Hindlimbs -
size 
    
 
 
  3.3.44  Hindpaw -
shape 
    
 
 
  3.3.45  Hindpaw -
size 
    
 
 
  3.3.46  Limb grasp 
    
 
 
  3.3.47  Lower lip
morphology 
    
 
 
  3.3.48  Lower teeth
appearance 
    
 
 
  3.3.49  Mouth
morphology 
    
 
 
  3.3.50  Skin color - back
paws 
    
 
 
  3.3.51  Skin color -
ear 
    
 
 
  3.3.52  Skin color - front
paws 
    
 
 
  3.3.53  Skin color -
snout 
    
 
 
  3.3.54  Skin color -
tail 
    
 
 
  3.3.55  Skin color - whole
body 
    
 
 
  3.3.56  Skin texture -
back paws 
    
 
 
  3.3.57  Skin texture -
ear 
    
 
 
  3.3.58  Skin texture -
front paws 
    
 
 
  3.3.59  Skin texture -
snout 
    
 
 
  3.3.60  Skin texture -
tail 
    
 
 
  3.3.61  Skin texture -
whole body 
    
 
 
  3.3.62  Snout size 
    
 
 
  3.3.63  Startle
response 
    
 
 
  3.3.64  Tail - length 
    
 
 
  3.3.65  Tail -
morphology 
    
 
 
  3.3.66  Tail -
presence 
    
 
 
  3.3.67  Tail -
thickness 
    
 
 
  3.3.68  Tail
elevation 
    
 
 
  3.3.69  Teeth
presence 
    
 
 
  3.3.70  Transfer
arousal 
 
 
 
  
 female WT/WT 
 female Δ HEL/WT 
 male WT/WT 
 
 
 
 
 female Δ HEL/WT 
 1 
 NA 
 NA 
 
 
 male WT/WT 
 1 
 1 
 NA 
 
 
 male Δ HEL/WT 
 1 
 1 
 1 
 
 
 
   
    
 
 
  3.3.71  Tremor 
    
 
 
  3.3.72  Trunk curl 
    
 
 
  3.3.73  Unexpected
behaviors 
    
 
 
  3.3.74  Upper lip
morphology 
    
 
 
  3.3.75  Upper teeth
appearance 
    
 
 
  3.3.76  Vibrissae -
appearance 
    
 
 
  3.3.77  Vibrissae -
presence 
    
 
 
  3.3.78  Vocalization 
    
 
 
 
  3.4  Grip Strength 
  Number of animals  
   
 
 
 
 STRAIN 
 GENDER 
 count 
 
 
 
 
 WT/WT 
 female 
 8 
 
 
 WT/WT 
 male 
 8 
 
 
 Δ HEL/WT 
 female 
 8 
 
 
 Δ HEL/WT 
 male 
 8 
 
 
 
   
 
 
 
 
 
 
 
 
 
 
  
 test 
 Difference 
 p_value 
 Significance 
 
 
 
 
 c2 
 WT/WT vs Δ HEL/WT 
 -1.8908 
 0.9975 
 FALSE 
 
 
 c3 
 Female vs Male 
 -26.1839 
 0.0466 
 TRUE 
 
 
 c4 
 Female WT/WT vs Female Δ HEL/WT 
 7.1084 
 0.9565 
 FALSE 
 
 
 c5 
 Male WT/WT vs Male Δ HEL/WT 
 -10.8900 
 0.8629 
 FALSE 
 
 
 c6 
 Female Δ HEL/WT vs Male Δ HEL/WT 
 -35.1831 
 0.0645 
 FALSE 
 
 
 
      
 
 
 
 
 
 
 
 
 
 
  
 test 
 Difference 
 p_value 
 Significance 
 
 
 
 
 c2 
 WT/WT vs Δ HEL/WT 
 24.9873 
 0.1319 
 FALSE 
 
 
 c3 
 Female vs Male 
 -35.8054 
 0.0115 
 TRUE 
 
 
 c4 
 Female WT/WT vs Female Δ HEL/WT 
 7.4437 
 0.9665 
 FALSE 
 
 
 c5 
 Male WT/WT vs Male Δ HEL/WT 
 42.5309 
 0.0467 
 TRUE 
 
 
 c6 
 Female Δ HEL/WT vs Male Δ HEL/WT 
 -18.2618 
 0.6667 
 FALSE 
 
 
 
     
 
 
  3.5  Light Dark Box 
  Number of animals  
   
 
 
 
 STRAIN 
 GENDER 
 count 
 
 
 
 
 WT/WT 
 female 
 8 
 
 
 WT/WT 
 male 
 8 
 
 
 Δ HEL/WT 
 female 
 8 
 
 
 Δ HEL/WT 
 male 
 8 
 
 
 
   
 
  3.5.1  Dark side time
spent 
   
 
 
 
 
 
 
 
 
 
 
  
 test 
 Difference 
 p_value 
 Significance 
 
 
 
 
 c2 
 WT/WT vs Δ HEL/WT 
 19.4188 
 0.3704 
 FALSE 
 
 
 c3 
 Female vs Male 
 13.9813 
 0.6373 
 FALSE 
 
 
 c4 
 Female WT/WT vs Female Δ HEL/WT 
 49.5500 
 0.0320 
 TRUE 
 
 
 c5 
 Male WT/WT vs Male Δ HEL/WT 
 -10.7125 
 0.9151 
 FALSE 
 
 
 c6 
 Female Δ HEL/WT vs Male Δ HEL/WT 
 -16.1500 
 0.7645 
 FALSE 
 
 
 
   
    
 
 
  3.5.2  Light side time
spent 
   
 
 
 
 
 
 
 
 
 
 
  
 test 
 Difference 
 p_value 
 Significance 
 
 
 
 
 c2 
 WT/WT vs Δ HEL/WT 
 -19.8937 
 0.3647 
 FALSE 
 
 
 c3 
 Female vs Male 
 -17.3438 
 0.4812 
 FALSE 
 
 
 c4 
 Female WT/WT vs Female Δ HEL/WT 
 -50.0000 
 0.0340 
 TRUE 
 
 
 c5 
 Male WT/WT vs Male Δ HEL/WT 
 10.2125 
 0.9287 
 FALSE 
 
 
 c6 
 Female Δ HEL/WT vs Male Δ HEL/WT 
 12.7625 
 0.8720 
 FALSE 
 
 
 
   
    
 
 
  3.5.3  Latency to first
transition 
   
 
 
 
 
 
 
 
 
 
 
  
 test 
 Difference 
 p_value 
 Significance 
 
 
 
 
 c2 
 WT/WT vs Δ HEL/WT 
 3.2437 
 0.8456 
 FALSE 
 
 
 c3 
 Female vs Male 
 1.7688 
 0.9697 
 FALSE 
 
 
 c4 
 Female WT/WT vs Female Δ HEL/WT 
 11.6500 
 0.1952 
 FALSE 
 
 
 c5 
 Male WT/WT vs Male Δ HEL/WT 
 -5.1625 
 0.7947 
 FALSE 
 
 
 c6 
 Female Δ HEL/WT vs Male Δ HEL/WT 
 -6.6375 
 0.6449 
 FALSE 
 
 
 
   
    
 
 
  3.5.4  Side changes 
   
 
 
 
 
 
 
 
 
 
 
  
 test 
 Difference 
 p_value 
 Significance 
 
 
 
 
 c2 
 WT/WT vs Δ HEL/WT 
 -1.4375 
 0.9746 
 FALSE 
 
 
 c3 
 Female vs Male 
 -3.1875 
 0.7901 
 FALSE 
 
 
 c4 
 Female WT/WT vs Female Δ HEL/WT 
 -13.7500 
 0.0454 
 TRUE 
 
 
 c5 
 Male WT/WT vs Male Δ HEL/WT 
 10.8750 
 0.1464 
 FALSE 
 
 
 c6 
 Female Δ HEL/WT vs Male Δ HEL/WT 
 9.1250 
 0.2684 
 FALSE 
 
 
 
   
    
 
 
  3.5.5  Time mobile active
dark side 
   
 
 
 
 
 
 
 
 
 
 
  
 test 
 Difference 
 p_value 
 Significance 
 
 
 
 
 c2 
 WT/WT vs Δ HEL/WT 
 7.6271 
 0.2871 
 FALSE 
 
 
 c3 
 Female vs Male 
 3.1730 
 0.8696 
 FALSE 
 
 
 c4 
 Female WT/WT vs Female Δ HEL/WT 
 6.9588 
 0.6437 
 FALSE 
 
 
 c5 
 Male WT/WT vs Male Δ HEL/WT 
 8.2953 
 0.5074 
 FALSE 
 
 
 c6 
 Female Δ HEL/WT vs Male Δ HEL/WT 
 3.8413 
 0.9129 
 FALSE 
 
 
 
   
    
 
 
  3.5.6  Time mobile active
light side 
   
 
 
 
 
 
 
 
 
 
 
  
 test 
 Difference 
 p_value 
 Significance 
 
 
 
 
 c2 
 WT/WT vs Δ HEL/WT 
 -0.7371 
 0.9988 
 FALSE 
 
 
 c3 
 Female vs Male 
 -35.7688 
 0.0000 
 TRUE 
 
 
 c4 
 Female WT/WT vs Female Δ HEL/WT 
 -4.1914 
 0.9336 
 FALSE 
 
 
 c5 
 Male WT/WT vs Male Δ HEL/WT 
 2.7172 
 0.9803 
 FALSE 
 
 
 c6 
 Female Δ HEL/WT vs Male Δ HEL/WT 
 -32.3145 
 0.0006 
 TRUE 
 
 
 
   
    
 
 
 
  3.6  Acustic
startle/PPI 
  Number of animals  
   
 
 
 
 STRAIN 
 GENDER 
 count 
 
 
 
 
 WT/WT 
 female 
 8 
 
 
 WT/WT 
 male 
 8 
 
 
 Δ HEL/WT 
 female 
 8 
 
 
 Δ HEL/WT 
 male 
 8 
 
 
 
   
 
  3.6.1  % Pre-pulse
inhibition - Global 
   
 
 
 
 
 
 
 
 
 
 
  
 test 
 Difference 
 p_value 
 Significance 
 
 
 
 
 c2 
 WT/WT vs Δ HEL/WT 
 11.1966 
 0.6283 
 FALSE 
 
 
 c3 
 Female vs Male 
 -13.2912 
 0.4881 
 FALSE 
 
 
 c4 
 Female WT/WT vs Female Δ HEL/WT 
 20.6555 
 0.4047 
 FALSE 
 
 
 c5 
 Male WT/WT vs Male Δ HEL/WT 
 1.7377 
 0.9992 
 FALSE 
 
 
 c6 
 Female Δ HEL/WT vs Male Δ HEL/WT 
 -22.7501 
 0.3191 
 FALSE 
 
 
 
      
 
 
  3.6.2  % Pre-pulse
inhibition - PPI1 
   
 
 
 
 
 
 
 
 
 
 
  
 test 
 Difference 
 p_value 
 Significance 
 
 
 
 
 c2 
 WT/WT vs Δ HEL/WT 
 9.4707 
 0.8267 
 FALSE 
 
 
 c3 
 Female vs Male 
 -7.7241 
 0.8970 
 FALSE 
 
 
 c4 
 Female WT/WT vs Female Δ HEL/WT 
 24.9917 
 0.3922 
 FALSE 
 
 
 c5 
 Male WT/WT vs Male Δ HEL/WT 
 -6.0503 
 0.9806 
 FALSE 
 
 
 c6 
 Female Δ HEL/WT vs Male Δ HEL/WT 
 -23.2451 
 0.4595 
 FALSE 
 
 
 
      
 
 
  3.6.3  % Pre-pulse
inhibition - PPI2 
   
 
 
 
 
 
 
 
 
 
 
  
 test 
 Difference 
 p_value 
 Significance 
 
 
 
 
 c2 
 WT/WT vs Δ HEL/WT 
 11.7113 
 0.4912 
 FALSE 
 
 
 c3 
 Female vs Male 
 -13.2830 
 0.3795 
 FALSE 
 
 
 c4 
 Female WT/WT vs Female Δ HEL/WT 
 20.8892 
 0.2863 
 FALSE 
 
 
 c5 
 Male WT/WT vs Male Δ HEL/WT 
 2.5335 
 0.9963 
 FALSE 
 
 
 c6 
 Female Δ HEL/WT vs Male Δ HEL/WT 
 -22.4608 
 0.2268 
 FALSE 
 
 
 
      
 
 
  3.6.4  % Pre-pulse
inhibition - PPI3 
   
 
 
 
 
 
 
 
 
 
 
  
 test 
 Difference 
 p_value 
 Significance 
 
 
 
 
 c2 
 WT/WT vs Δ HEL/WT 
 16.3316 
 0.5345 
 FALSE 
 
 
 c3 
 Female vs Male 
 -18.1929 
 0.4408 
 FALSE 
 
 
 c4 
 Female WT/WT vs Female Δ HEL/WT 
 24.1389 
 0.4969 
 FALSE 
 
 
 c5 
 Male WT/WT vs Male Δ HEL/WT 
 8.5242 
 0.9591 
 FALSE 
 
 
 c6 
 Female Δ HEL/WT vs Male Δ HEL/WT 
 -26.0002 
 0.4314 
 FALSE 
 
 
 
      
 
 
  3.6.5  % Pre-pulse
inhibition - PPI4 
   
 
 
 
 
 
 
 
 
 
 
  
 test 
 Difference 
 p_value 
 Significance 
 
 
 
 
 c2 
 WT/WT vs Δ HEL/WT 
 7.7521 
 0.7965 
 FALSE 
 
 
 c3 
 Female vs Male 
 -13.4853 
 0.3899 
 FALSE 
 
 
 c4 
 Female WT/WT vs Female Δ HEL/WT 
 14.3288 
 0.6311 
 FALSE 
 
 
 c5 
 Male WT/WT vs Male Δ HEL/WT 
 1.1755 
 0.9997 
 FALSE 
 
 
 c6 
 Female Δ HEL/WT vs Male Δ HEL/WT 
 -20.0620 
 0.3446 
 FALSE 
 
 
 
      
 
 
 
  3.7  Fear
Conditioning 
  Number of animals  
   
 
 
 
 STRAIN 
 GENDER 
 count 
 
 
 
 
 WT/WT 
 female 
 8 
 
 
 WT/WT 
 male 
 8 
 
 
 Δ HEL/WT 
 female 
 8 
 
 
 Δ HEL/WT 
 male 
 8 
 
 
 
   
     
 
 
  3.8  Indirect
Calorimetry 
  Number of animals  
   
 
 
 
 STRAIN 
 GENDER 
 count 
 
 
 
 
 WT/WT 
 male 
 7 
 
 
 Δ HEL/WT 
 male 
 8 
 
 
 
 
  3.8.1  Oxygen
consumption 
 
 
 
 test 
 Difference 
 p_value 
 Significance 
 
 
 
 
 CWT/WT vs Δ HEL/WT 
 126.2974 
 0.3333 
 FALSE 
 
 
 
   
   
   
   
 
 
  3.8.2  Respiratory
Exchange Ratio 
 
 
 
 test 
 Difference 
 p_value 
 Significance 
 
 
 
 
 CWT/WT vs Δ HEL/WT 
 0.2025 
 0.2867 
 FALSE 
 
 
 
   
   
   
   
 
 
  3.8.3  Heat production
(metabolic rate) 
 
 
 
 test 
 Difference 
 p_value 
 Significance 
 
 
 
 
 CWT/WT vs Δ HEL/WT 
 1.4017 
 0.4576 
 FALSE 
 
 
 
   
   
   
   
 
 
  3.8.4  Ambulatory activity
(no. of beam cuts) 
 
 
 
 test 
 Difference 
 p_value 
 Significance 
 
 
 
 
 CWT/WT vs Δ HEL/WT 
 26.5653 
 0.0394 
 TRUE 
 
 
 
   
   
   
 
 
  3.8.5  Total movement 
 
 
 
 test 
 Difference 
 p_value 
 Significance 
 
 
 
 
 CWT/WT vs Δ HEL/WT 
 307.636 
 0.0091 
 TRUE 
 
 
 
   
   
   
 
 
  3.8.6  Cumulative food
intake 
 
 
 
 test 
 Difference 
 p_value 
 Significance 
 
 
 
 
 CWT/WT vs Δ HEL/WT 
 -0.807 
 0.8863 
 FALSE 
 
 
 
   
   
   
   
 
 
  3.8.7  Cumulative water
intake 
 
 
 
 test 
 Difference 
 p_value 
 Significance 
 
 
 
 
 CWT/WT vs Δ HEL/WT 
 -5.9333 
 0.4876 
 FALSE 
 
 
 
   
   
   
   
 
 
  3.8.8  Carbon dioxide
production per kg 
 
 
 
 test 
 Difference 
 p_value 
 Significance 
 
 
 
 
 CWT/WT vs Δ HEL/WT 
 33.0785 
 0.868 
 FALSE 
 
 
 
   
   
   
 
 
  3.8.9  Oxygen consumption
per kg 
 
 
 
 test 
 Difference 
 p_value 
 Significance 
 
 
 
 
 CWT/WT vs Δ HEL/WT 
 53.074 
 0.8166 
 FALSE 
 
 
 
   
   
   
 
 
  3.8.10  DistD 
 
 
 
 test 
 Difference 
 p_value 
 Significance 
 
 
 
 
 CWT/WT vs Δ HEL/WT 
 848.8899 
 0.0678 
 FALSE 
 
 
 
   
   
   
 
 
  3.8.11  DistK 
 
 
 
 test 
 Difference 
 p_value 
 Significance 
 
 
 
 
 CWT/WT vs Δ HEL/WT 
 69588.7 
 0.0042 
 TRUE 
 
 
 
   
   
   
 
 
  3.8.12  Z 
 
 
 
 test 
 Difference 
 p_value 
 Significance 
 
 
 
 
 CWT/WT vs Δ HEL/WT 
 62.8633 
 0.0309 
 TRUE 
 
 
 
   
   
   
   
 
 
 
  3.9  Echocardiography 
  Number of animals  
   
 
 
 
 STRAIN 
 GENDER 
 count 
 
 
 
 
 WT/WT 
 female 
 8 
 
 
 WT/WT 
 male 
 8 
 
 
 Δ HEL/WT 
 female 
 7 
 
 
 Δ HEL/WT 
 male 
 8 
 
 
 
   
 
  3.9.1  Aortic diameter
(Dao) 
 
 
 
 
 
 
 
 
 
 
  
 test 
 Difference 
 p_value 
 Significance 
 
 
 
 
 c2 
 WT/WT vs Δ HEL/WT 
 0.0413 
 0.5249 
 FALSE 
 
 
 c3 
 Female vs Male 
 0.0295 
 0.8560 
 FALSE 
 
 
 c4 
 Female WT/WT vs Female Δ HEL/WT 
 0.0398 
 0.7954 
 FALSE 
 
 
 c5 
 Male WT/WT vs Male Δ HEL/WT 
 0.0427 
 0.7400 
 FALSE 
 
 
 c6 
 Female Δ HEL/WT vs Male Δ HEL/WT 
 0.0309 
 0.9193 
 FALSE 
 
 
 
     
 
 
  3.9.2  Cardiac Output 
 
 
 
 
 
 
 
 
 
 
  
 test 
 Difference 
 p_value 
 Significance 
 
 
 
 
 c2 
 WT/WT vs Δ HEL/WT 
 1.9593 
 0.5580 
 FALSE 
 
 
 c3 
 Female vs Male 
 -5.0347 
 0.5328 
 FALSE 
 
 
 c4 
 Female WT/WT vs Female Δ HEL/WT 
 2.9704 
 0.5138 
 FALSE 
 
 
 c5 
 Male WT/WT vs Male Δ HEL/WT 
 0.9482 
 0.9679 
 FALSE 
 
 
 c6 
 Female Δ HEL/WT vs Male Δ HEL/WT 
 -6.0458 
 0.4427 
 FALSE 
 
 
 
     
 
 
  3.9.3  Ejection
Fraction 
 
 
 
 
 
 
 
 
 
 
  
 test 
 Difference 
 p_value 
 Significance 
 
 
 
 
 c2 
 WT/WT vs Δ HEL/WT 
 -1.2780 
 0.9543 
 FALSE 
 
 
 c3 
 Female vs Male 
 2.2724 
 0.8647 
 FALSE 
 
 
 c4 
 Female WT/WT vs Female Δ HEL/WT 
 5.2137 
 0.4616 
 FALSE 
 
 
 c5 
 Male WT/WT vs Male Δ HEL/WT 
 -7.7696 
 0.1160 
 FALSE 
 
 
 c6 
 Female Δ HEL/WT vs Male Δ HEL/WT 
 -4.2192 
 0.6986 
 FALSE 
 
 
 
     
 
 
  3.9.4  End-Diastolic
Diameter 
 
 
 
 
 
 
 
 
 
 
  
 test 
 Difference 
 p_value 
 Significance 
 
 
 
 
 c2 
 WT/WT vs Δ HEL/WT 
 0.2093 
 0.1172 
 FALSE 
 
 
 c3 
 Female vs Male 
 -0.3118 
 0.5636 
 FALSE 
 
 
 c4 
 Female WT/WT vs Female Δ HEL/WT 
 0.0494 
 0.9824 
 FALSE 
 
 
 c5 
 Male WT/WT vs Male Δ HEL/WT 
 0.3693 
 0.0263 
 TRUE 
 
 
 c6 
 Female Δ HEL/WT vs Male Δ HEL/WT 
 -0.1518 
 0.9348 
 FALSE 
 
 
 
     
 
 
  3.9.5  End-Systolic
Diameter 
 
 
 
 
 
 
 
 
 
 
  
 test 
 Difference 
 p_value 
 Significance 
 
 
 
 
 c2 
 WT/WT vs Δ HEL/WT 
 0.1850 
 0.3036 
 FALSE 
 
 
 c3 
 Female vs Male 
 -0.2809 
 0.7866 
 FALSE 
 
 
 c4 
 Female WT/WT vs Female Δ HEL/WT 
 -0.1088 
 0.8892 
 FALSE 
 
 
 c5 
 Male WT/WT vs Male Δ HEL/WT 
 0.4789 
 0.0084 
 TRUE 
 
 
 c6 
 Female Δ HEL/WT vs Male Δ HEL/WT 
 0.0129 
 1.0000 
 FALSE 
 
 
 
     
 
 
  3.9.6  Fractional
Shortening 
 
 
 
 
 
 
 
 
 
 
  
 test 
 Difference 
 p_value 
 Significance 
 
 
 
 
 c2 
 WT/WT vs Δ HEL/WT 
 -0.7206 
 0.9684 
 FALSE 
 
 
 c3 
 Female vs Male 
 1.3830 
 0.8782 
 FALSE 
 
 
 c4 
 Female WT/WT vs Female Δ HEL/WT 
 3.4105 
 0.4457 
 FALSE 
 
 
 c5 
 Male WT/WT vs Male Δ HEL/WT 
 -4.8517 
 0.1326 
 FALSE 
 
 
 c6 
 Female Δ HEL/WT vs Male Δ HEL/WT 
 -2.7481 
 0.6846 
 FALSE 
 
 
 
     
 
 
  3.9.7  Heart rate 
 
 
 
 
 
 
 
 
 
 
  
 test 
 Difference 
 p_value 
 Significance 
 
 
 
 
 c2 
 WT/WT vs Δ HEL/WT 
 0.9054 
 1.0000 
 FALSE 
 
 
 c3 
 Female vs Male 
 -52.1438 
 0.5883 
 FALSE 
 
 
 c4 
 Female WT/WT vs Female Δ HEL/WT 
 6.4704 
 0.9945 
 FALSE 
 
 
 c5 
 Male WT/WT vs Male Δ HEL/WT 
 -4.6595 
 0.9977 
 FALSE 
 
 
 c6 
 Female Δ HEL/WT vs Male Δ HEL/WT 
 -57.7088 
 0.5818 
 FALSE 
 
 
 
     
 
 
  3.9.8  Left ventricle
anterior wall (diastole) 
 
 
 
 
 
 
 
 
 
 
  
 test 
 Difference 
 p_value 
 Significance 
 
 
 
 
 c2 
 WT/WT vs Δ HEL/WT 
 -0.0297 
 0.8977 
 FALSE 
 
 
 c3 
 Female vs Male 
 0.0287 
 0.9283 
 FALSE 
 
 
 c4 
 Female WT/WT vs Female Δ HEL/WT 
 -0.0187 
 0.9901 
 FALSE 
 
 
 c5 
 Male WT/WT vs Male Δ HEL/WT 
 -0.0407 
 0.9012 
 FALSE 
 
 
 c6 
 Female Δ HEL/WT vs Male Δ HEL/WT 
 0.0177 
 0.9927 
 FALSE 
 
 
 
     
 
 
  3.9.9  Left ventricle
anterior wall (systole) 
 
 
 
 
 
 
 
 
 
 
  
 test 
 Difference 
 p_value 
 Significance 
 
 
 
 
 c2 
 WT/WT vs Δ HEL/WT 
 -0.0328 
 0.9196 
 FALSE 
 
 
 c3 
 Female vs Male 
 0.0432 
 0.8882 
 FALSE 
 
 
 c4 
 Female WT/WT vs Female Δ HEL/WT 
 0.0659 
 0.8106 
 FALSE 
 
 
 c5 
 Male WT/WT vs Male Δ HEL/WT 
 -0.1315 
 0.2662 
 FALSE 
 
 
 c6 
 Female Δ HEL/WT vs Male Δ HEL/WT 
 -0.0555 
 0.8997 
 FALSE 
 
 
 
     
 
 
  3.9.10  Left ventricle
interior diameter (diastole) 
 
 
 
 
 
 
 
 
 
 
  
 test 
 Difference 
 p_value 
 Significance 
 
 
 
 
 c2 
 WT/WT vs Δ HEL/WT 
 0.2352 
 0.0971 
 FALSE 
 
 
 c3 
 Female vs Male 
 -0.2409 
 0.5384 
 FALSE 
 
 
 c4 
 Female WT/WT vs Female Δ HEL/WT 
 0.0248 
 0.9982 
 FALSE 
 
 
 c5 
 Male WT/WT vs Male Δ HEL/WT 
 0.4457 
 0.0102 
 TRUE 
 
 
 c6 
 Female Δ HEL/WT vs Male Δ HEL/WT 
 -0.0305 
 0.9988 
 FALSE 
 
 
 
     
 
 
  3.9.11  Left ventricle
interior diameter (systole) 
 
 
 
 
 
 
 
 
 
 
  
 test 
 Difference 
 p_value 
 Significance 
 
 
 
 
 c2 
 WT/WT vs Δ HEL/WT 
 0.2234 
 0.3106 
 FALSE 
 
 
 c3 
 Female vs Male 
 -0.1744 
 0.7280 
 FALSE 
 
 
 c4 
 Female WT/WT vs Female Δ HEL/WT 
 -0.1575 
 0.8278 
 FALSE 
 
 
 c5 
 Male WT/WT vs Male Δ HEL/WT 
 0.6043 
 0.0054 
 TRUE 
 
 
 c6 
 Female Δ HEL/WT vs Male Δ HEL/WT 
 0.2065 
 0.7697 
 FALSE 
 
 
 
     
 
 
  3.9.12  Left ventricle
posterior wall (diastole) 
 
 
 
 
 
 
 
 
 
 
  
 test 
 Difference 
 p_value 
 Significance 
 
 
 
 
 c2 
 WT/WT vs Δ HEL/WT 
 0.0234 
 0.9727 
 FALSE 
 
 
 c3 
 Female vs Male 
 -0.0977 
 0.6470 
 FALSE 
 
 
 c4 
 Female WT/WT vs Female Δ HEL/WT 
 0.1245 
 0.3876 
 FALSE 
 
 
 c5 
 Male WT/WT vs Male Δ HEL/WT 
 -0.0777 
 0.7313 
 FALSE 
 
 
 c6 
 Female Δ HEL/WT vs Male Δ HEL/WT 
 -0.1988 
 0.2066 
 FALSE 
 
 
 
     
 
 
  3.9.13  Left ventricle
posterior wall (systole) 
 
 
 
 
 
 
 
 
 
 
  
 test 
 Difference 
 p_value 
 Significance 
 
 
 
 
 c2 
 WT/WT vs Δ HEL/WT 
 -0.0409 
 0.9520 
 FALSE 
 
 
 c3 
 Female vs Male 
 -0.1064 
 0.8706 
 FALSE 
 
 
 c4 
 Female WT/WT vs Female Δ HEL/WT 
 0.1464 
 0.5579 
 FALSE 
 
 
 c5 
 Male WT/WT vs Male Δ HEL/WT 
 -0.2281 
 0.1578 
 FALSE 
 
 
 c6 
 Female Δ HEL/WT vs Male Δ HEL/WT 
 -0.2936 
 0.2708 
 FALSE 
 
 
 
     
 
 
  3.9.14  Stroke Volume 
 
 
 
 
 
 
 
 
 
 
  
 test 
 Difference 
 p_value 
 Significance 
 
 
 
 
 c2 
 WT/WT vs Δ HEL/WT 
 4.0597 
 0.3638 
 FALSE 
 
 
 c3 
 Female vs Male 
 -5.8412 
 0.5835 
 FALSE 
 
 
 c4 
 Female WT/WT vs Female Δ HEL/WT 
 5.5459 
 0.4099 
 FALSE 
 
 
 c5 
 Male WT/WT vs Male Δ HEL/WT 
 2.5734 
 0.8769 
 FALSE 
 
 
 c6 
 Female Δ HEL/WT vs Male Δ HEL/WT 
 -7.3275 
 0.5075 
 FALSE 
 
 
 
     
 
 
 
  3.10  ECG 
  Number of animals  
   
 
 
 
 STRAIN 
 GENDER 
 count 
 
 
 
 
 WT/WT 
 female 
 8 
 
 
 WT/WT 
 male 
 8 
 
 
 Δ HEL/WT 
 female 
 7 
 
 
 Δ HEL/WT 
 male 
 7 
 
 
 
   
 
  3.10.1  Heart rate 
 
 
 
 
 
 
 
 
 
 
  
 test 
 Difference 
 p_value 
 Significance 
 
 
 
 
 c2 
 WT/WT vs Δ HEL/WT 
 14.2857 
 0.8952 
 FALSE 
 
 
 c3 
 Female vs Male 
 -50.1964 
 0.0993 
 FALSE 
 
 
 c4 
 Female WT/WT vs Female Δ HEL/WT 
 14.2321 
 0.9594 
 FALSE 
 
 
 c5 
 Male WT/WT vs Male Δ HEL/WT 
 14.3393 
 0.9585 
 FALSE 
 
 
 c6 
 Female Δ HEL/WT vs Male Δ HEL/WT 
 -50.1429 
 0.3605 
 FALSE 
 
 
 
    
 
 
  3.10.2  HRV 
 
 
 
 
 
 
 
 
 
 
  
 test 
 Difference 
 p_value 
 Significance 
 
 
 
 
 c2 
 WT/WT vs Δ HEL/WT 
 -3.8089 
 0.7640 
 FALSE 
 
 
 c3 
 Female vs Male 
 8.8911 
 0.1387 
 FALSE 
 
 
 c4 
 Female WT/WT vs Female Δ HEL/WT 
 -1.0750 
 0.9973 
 FALSE 
 
 
 c5 
 Male WT/WT vs Male Δ HEL/WT 
 -6.5429 
 0.6428 
 FALSE 
 
 
 c6 
 Female Δ HEL/WT vs Male Δ HEL/WT 
 6.1571 
 0.7050 
 FALSE 
 
 
 
    
 
 
  3.10.3  Mean R
amplitude 
 
 
 
 
 
 
 
 
 
 
  
 test 
 Difference 
 p_value 
 Significance 
 
 
 
 
 c2 
 WT/WT vs Δ HEL/WT 
 -0.0258 
 0.9793 
 FALSE 
 
 
 c3 
 Female vs Male 
 -0.0119 
 0.9979 
 FALSE 
 
 
 c4 
 Female WT/WT vs Female Δ HEL/WT 
 -0.0577 
 0.9257 
 FALSE 
 
 
 c5 
 Male WT/WT vs Male Δ HEL/WT 
 0.0061 
 0.9999 
 FALSE 
 
 
 c6 
 Female Δ HEL/WT vs Male Δ HEL/WT 
 0.0200 
 0.9968 
 FALSE 
 
 
 
    
 
 
  3.10.4  Mean SR
amplitude 
 
 
 
 
 
 
 
 
 
 
  
 test 
 Difference 
 p_value 
 Significance 
 
 
 
 
 c2 
 WT/WT vs Δ HEL/WT 
 -0.0391 
 0.9886 
 FALSE 
 
 
 c3 
 Female vs Male 
 0.0654 
 0.9507 
 FALSE 
 
 
 c4 
 Female WT/WT vs Female Δ HEL/WT 
 -0.0395 
 0.9958 
 FALSE 
 
 
 c5 
 Male WT/WT vs Male Δ HEL/WT 
 -0.0388 
 0.9960 
 FALSE 
 
 
 c6 
 Female Δ HEL/WT vs Male Δ HEL/WT 
 0.0657 
 0.9829 
 FALSE 
 
 
 
    
 
 
  3.10.5  PQ 
 
 
 
 
 
 
 
 
 
 
  
 test 
 Difference 
 p_value 
 Significance 
 
 
 
 
 c2 
 WT/WT vs Δ HEL/WT 
 -0.1054 
 0.9994 
 FALSE 
 
 
 c3 
 Female vs Male 
 1.8018 
 0.2346 
 FALSE 
 
 
 c4 
 Female WT/WT vs Female Δ HEL/WT 
 0.1679 
 0.9992 
 FALSE 
 
 
 c5 
 Male WT/WT vs Male Δ HEL/WT 
 -0.3786 
 0.9909 
 FALSE 
 
 
 c6 
 Female Δ HEL/WT vs Male Δ HEL/WT 
 1.5286 
 0.6677 
 FALSE 
 
 
 
    
 
 
  3.10.6  PR 
 
 
 
 
 
 
 
 
 
 
  
 test 
 Difference 
 p_value 
 Significance 
 
 
 
 
 c2 
 WT/WT vs Δ HEL/WT 
 -0.1295 
 0.9995 
 FALSE 
 
 
 c3 
 Female vs Male 
 1.8170 
 0.4183 
 FALSE 
 
 
 c4 
 Female WT/WT vs Female Δ HEL/WT 
 0.0161 
 1.0000 
 FALSE 
 
 
 c5 
 Male WT/WT vs Male Δ HEL/WT 
 -0.2750 
 0.9982 
 FALSE 
 
 
 c6 
 Female Δ HEL/WT vs Male Δ HEL/WT 
 1.6714 
 0.7565 
 FALSE 
 
 
 
    
 
 
  3.10.7  QRS 
 
 
 
 
 
 
 
 
 
 
  
 test 
 Difference 
 p_value 
 Significance 
 
 
 
 
 c2 
 WT/WT vs Δ HEL/WT 
 -0.1134 
 0.9783 
 FALSE 
 
 
 c3 
 Female vs Male 
 -0.0812 
 0.9917 
 FALSE 
 
 
 c4 
 Female WT/WT vs Female Δ HEL/WT 
 0.1054 
 0.9936 
 FALSE 
 
 
 c5 
 Male WT/WT vs Male Δ HEL/WT 
 -0.3321 
 0.8444 
 FALSE 
 
 
 c6 
 Female Δ HEL/WT vs Male Δ HEL/WT 
 -0.3000 
 0.8891 
 FALSE 
 
 
 
    
 
 
  3.10.8  QT 
 
 
 
 
 
 
 
 
 
 
  
 test 
 Difference 
 p_value 
 Significance 
 
 
 
 
 c2 
 WT/WT vs Δ HEL/WT 
 -0.6661 
 0.9536 
 FALSE 
 
 
 c3 
 Female vs Male 
 2.8036 
 0.1661 
 FALSE 
 
 
 c4 
 Female WT/WT vs Female Δ HEL/WT 
 -0.1196 
 0.9999 
 FALSE 
 
 
 c5 
 Male WT/WT vs Male Δ HEL/WT 
 -1.2125 
 0.9082 
 FALSE 
 
 
 c6 
 Female Δ HEL/WT vs Male Δ HEL/WT 
 2.2571 
 0.6343 
 FALSE 
 
 
 
    
 
 
  3.10.9  QT_dispersion 
 
 
 
 
 
 
 
 
 
 
  
 test 
 Difference 
 p_value 
 Significance 
 
 
 
 
 c2 
 WT/WT vs Δ HEL/WT 
 -0.0964 
 0.9999 
 FALSE 
 
 
 c3 
 Female vs Male 
 5.7964 
 0.0090 
 TRUE 
 
 
 c4 
 Female WT/WT vs Female Δ HEL/WT 
 0.8571 
 0.9812 
 FALSE 
 
 
 c5 
 Male WT/WT vs Male Δ HEL/WT 
 -1.0500 
 0.9665 
 FALSE 
 
 
 c6 
 Female Δ HEL/WT vs Male Δ HEL/WT 
 4.8429 
 0.2043 
 FALSE 
 
 
 
    
 
 
  3.10.10  QTc 
 
 
 
 
 
 
 
 
 
 
  
 test 
 Difference 
 p_value 
 Significance 
 
 
 
 
 c2 
 WT/WT vs Δ HEL/WT 
 -0.2902 
 0.9875 
 FALSE 
 
 
 c3 
 Female vs Male 
 1.4330 
 0.3972 
 FALSE 
 
 
 c4 
 Female WT/WT vs Female Δ HEL/WT 
 0.3143 
 0.9943 
 FALSE 
 
 
 c5 
 Male WT/WT vs Male Δ HEL/WT 
 -0.8946 
 0.8909 
 FALSE 
 
 
 c6 
 Female Δ HEL/WT vs Male Δ HEL/WT 
 0.8286 
 0.9180 
 FALSE 
 
 
 
    
 
 
  3.10.11  QTc
Dispersion 
 
 
 
 
 
 
 
 
 
 
  
 test 
 Difference 
 p_value 
 Significance 
 
 
 
 
 c2 
 WT/WT vs Δ HEL/WT 
 0.1982 
 0.9991 
 FALSE 
 
 
 c3 
 Female vs Male 
 5.1554 
 0.0106 
 TRUE 
 
 
 c4 
 Female WT/WT vs Female Δ HEL/WT 
 0.9679 
 0.9655 
 FALSE 
 
 
 c5 
 Male WT/WT vs Male Δ HEL/WT 
 -0.5714 
 0.9924 
 FALSE 
 
 
 c6 
 Female Δ HEL/WT vs Male Δ HEL/WT 
 4.3857 
 0.2087 
 FALSE 
 
 
 
    
 
 
  3.10.12  RR 
 
 
 
 
 
 
 
 
 
 
  
 test 
 Difference 
 p_value 
 Significance 
 
 
 
 
 c2 
 WT/WT vs Δ HEL/WT 
 -1.5911 
 0.9164 
 FALSE 
 
 
 c3 
 Female vs Male 
 5.9786 
 0.1083 
 FALSE 
 
 
 c4 
 Female WT/WT vs Female Δ HEL/WT 
 -1.6696 
 0.9631 
 FALSE 
 
 
 c5 
 Male WT/WT vs Male Δ HEL/WT 
 -1.5125 
 0.9721 
 FALSE 
 
 
 c6 
 Female Δ HEL/WT vs Male Δ HEL/WT 
 6.0571 
 0.3648 
 FALSE 
 
 
 
    
 
 
 
  3.11  Lung screen 
  Number of animals  
   
 
 
 
 STRAIN 
 GENDER 
 count 
 
 
 
 
 WT/WT 
 female 
 8 
 
 
 WT/WT 
 male 
 8 
 
 
 Δ HEL/WT 
 female 
 8 
 
 
 Δ HEL/WT 
 male 
 8 
 
 
 
   
 
  3.11.1  Compliance 
 
 
 
 
 
 
 
 
 
 
  
 test 
 Difference 
 p_value 
 Significance 
 
 
 
 
 c2 
 WT/WT vs Δ HEL/WT 
 0.0043 
 0.3763 
 FALSE 
 
 
 c3 
 Female vs Male 
 0.0011 
 0.9859 
 FALSE 
 
 
 c4 
 Female WT/WT vs Female Δ HEL/WT 
 0.0060 
 0.2549 
 FALSE 
 
 
 c5 
 Male WT/WT vs Male Δ HEL/WT 
 0.0026 
 0.8633 
 FALSE 
 
 
 c6 
 Female Δ HEL/WT vs Male Δ HEL/WT 
 -0.0005 
 0.9988 
 FALSE 
 
 
 
    
 
 
  3.11.2  Elastance 
 
 
 
 
 
 
 
 
 
 
  
 test 
 Difference 
 p_value 
 Significance 
 
 
 
 
 c2 
 WT/WT vs Δ HEL/WT 
 -1.0075 
 0.4927 
 FALSE 
 
 
 c3 
 Female vs Male 
 -0.1942 
 0.9962 
 FALSE 
 
 
 c4 
 Female WT/WT vs Female Δ HEL/WT 
 -1.4336 
 0.3471 
 FALSE 
 
 
 c5 
 Male WT/WT vs Male Δ HEL/WT 
 -0.5814 
 0.9182 
 FALSE 
 
 
 c6 
 Female Δ HEL/WT vs Male Δ HEL/WT 
 0.2319 
 0.9954 
 FALSE 
 
 
 
    
 
 
  3.11.3  Inspiratory
Capacity 
 
 
 
 
 
 
 
 
 
 
  
 test 
 Difference 
 p_value 
 Significance 
 
 
 
 
 c2 
 WT/WT vs Δ HEL/WT 
 0.0366 
 0.7723 
 FALSE 
 
 
 c3 
 Female vs Male 
 0.0257 
 0.9529 
 FALSE 
 
 
 c4 
 Female WT/WT vs Female Δ HEL/WT 
 0.0365 
 0.8493 
 FALSE 
 
 
 c5 
 Male WT/WT vs Male Δ HEL/WT 
 0.0367 
 0.8832 
 FALSE 
 
 
 c6 
 Female Δ HEL/WT vs Male Δ HEL/WT 
 0.0258 
 0.9648 
 FALSE 
 
 
 
    
 
 
  3.11.4  Resistance 
 
 
 
 
 
 
 
 
 
 
  
 test 
 Difference 
 p_value 
 Significance 
 
 
 
 
 c2 
 WT/WT vs Δ HEL/WT 
 0.0191 
 0.8756 
 FALSE 
 
 
 c3 
 Female vs Male 
 -0.0265 
 0.8560 
 FALSE 
 
 
 c4 
 Female WT/WT vs Female Δ HEL/WT 
 0.0403 
 0.5675 
 FALSE 
 
 
 c5 
 Male WT/WT vs Male Δ HEL/WT 
 -0.0021 
 0.9999 
 FALSE 
 
 
 c6 
 Female Δ HEL/WT vs Male Δ HEL/WT 
 -0.0477 
 0.5927 
 FALSE 
 
 
 
    
 
 
  3.11.5  Resistance of the
airways (Prime8) 
 
 
 
 
 
 
 
 
 
 
  
 test 
 Difference 
 p_value 
 Significance 
 
 
 
 
 c2 
 WT/WT vs Δ HEL/WT 
 0.0303 
 0.3004 
 FALSE 
 
 
 c3 
 Female vs Male 
 -0.0238 
 0.6902 
 FALSE 
 
 
 c4 
 Female WT/WT vs Female Δ HEL/WT 
 0.0388 
 0.2441 
 FALSE 
 
 
 c5 
 Male WT/WT vs Male Δ HEL/WT 
 0.0218 
 0.7508 
 FALSE 
 
 
 c6 
 Female Δ HEL/WT vs Male Δ HEL/WT 
 -0.0323 
 0.5499 
 FALSE 
 
 
 
    
 
 
  3.11.6  Resistance of the
airways (QuickPrime3) 
 
 
 
 
 
 
 
 
 
 
  
 test 
 Difference 
 p_value 
 Significance 
 
 
 
 
 c2 
 WT/WT vs Δ HEL/WT 
 0.0359 
 0.2681 
 FALSE 
 
 
 c3 
 Female vs Male 
 0.0008 
 1.0000 
 FALSE 
 
 
 c4 
 Female WT/WT vs Female Δ HEL/WT 
 0.0425 
 0.2710 
 FALSE 
 
 
 c5 
 Male WT/WT vs Male Δ HEL/WT 
 0.0292 
 0.6477 
 FALSE 
 
 
 c6 
 Female Δ HEL/WT vs Male Δ HEL/WT 
 -0.0059 
 0.9962 
 FALSE 
 
 
 
    
 
 
  3.11.7  Tissue damping
(Prime8) 
 
 
 
 
 
 
 
 
 
 
  
 test 
 Difference 
 p_value 
 Significance 
 
 
 
 
 c2 
 WT/WT vs Δ HEL/WT 
 -0.1933 
 0.2515 
 FALSE 
 
 
 c3 
 Female vs Male 
 -0.1052 
 0.8424 
 FALSE 
 
 
 c4 
 Female WT/WT vs Female Δ HEL/WT 
 -0.2624 
 0.1630 
 FALSE 
 
 
 c5 
 Male WT/WT vs Male Δ HEL/WT 
 -0.1241 
 0.7757 
 FALSE 
 
 
 c6 
 Female Δ HEL/WT vs Male Δ HEL/WT 
 -0.0361 
 0.9939 
 FALSE 
 
 
 
    
 
 
  3.11.8  Tissue damping
(QuickPrime3) 
 
 
 
 
 
 
 
 
 
 
  
 test 
 Difference 
 p_value 
 Significance 
 
 
 
 
 c2 
 WT/WT vs Δ HEL/WT 
 -0.1222 
 0.4226 
 FALSE 
 
 
 c3 
 Female vs Male 
 -0.1640 
 0.4005 
 FALSE 
 
 
 c4 
 Female WT/WT vs Female Δ HEL/WT 
 -0.1577 
 0.3562 
 FALSE 
 
 
 c5 
 Male WT/WT vs Male Δ HEL/WT 
 -0.0867 
 0.8289 
 FALSE 
 
 
 c6 
 Female Δ HEL/WT vs Male Δ HEL/WT 
 -0.1285 
 0.6696 
 FALSE 
 
 
 
    
 
 
  3.11.9  Tissue elastance
(Prime8) 
 
 
 
 
 
 
 
 
 
 
  
 test 
 Difference 
 p_value 
 Significance 
 
 
 
 
 c2 
 WT/WT vs Δ HEL/WT 
 -0.8563 
 0.7318 
 FALSE 
 
 
 c3 
 Female vs Male 
 -0.4213 
 0.9785 
 FALSE 
 
 
 c4 
 Female WT/WT vs Female Δ HEL/WT 
 -1.3815 
 0.5207 
 FALSE 
 
 
 c5 
 Male WT/WT vs Male Δ HEL/WT 
 -0.3311 
 0.9897 
 FALSE 
 
 
 c6 
 Female Δ HEL/WT vs Male Δ HEL/WT 
 0.1039 
 0.9998 
 FALSE 
 
 
 
    
 
 
  3.11.10  Tissue elastance
(QuickPrime3) 
 
 
 
 
 
 
 
 
 
 
  
 test 
 Difference 
 p_value 
 Significance 
 
 
 
 
 c2 
 WT/WT vs Δ HEL/WT 
 -1.1877 
 0.4685 
 FALSE 
 
 
 c3 
 Female vs Male 
 0.3121 
 0.9898 
 FALSE 
 
 
 c4 
 Female WT/WT vs Female Δ HEL/WT 
 -1.6666 
 0.3344 
 FALSE 
 
 
 c5 
 Male WT/WT vs Male Δ HEL/WT 
 -0.7088 
 0.9034 
 FALSE 
 
 
 c6 
 Female Δ HEL/WT vs Male Δ HEL/WT 
 0.7909 
 0.8995 
 FALSE 
 
 
 
    
   
 
 
 
  3.12  Intraperitoneal
Glucose Tolerance Test 
  Number of animals  
   
 
 
 
 STRAIN 
 GENDER 
 count 
 
 
 
 
 WT/WT 
 female 
 7 
 
 
 WT/WT 
 male 
 7 
 
 
 Δ HEL/WT 
 female 
 7 
 
 
 Δ HEL/WT 
 male 
 6 
 
 
 
   
   
   
   
 
 
 
 
 
 
 
 
 
 
  
 test 
 Difference 
 p_value 
 Significance 
 
 
 
 
 c2 
 WT/WT vs Δ HEL/WT 
 3.3349 
 0.9763 
 FALSE 
 
 
 c3 
 Female vs Male 
 3.5408 
 0.9871 
 FALSE 
 
 
 c4 
 Female WT/WT vs Female Δ HEL/WT 
 13.9165 
 0.4830 
 FALSE 
 
 
 c5 
 Male WT/WT vs Male Δ HEL/WT 
 -7.2468 
 0.9094 
 FALSE 
 
 
 c6 
 Female Δ HEL/WT vs Male Δ HEL/WT 
 -7.0409 
 0.9313 
 FALSE 
 
 
 
 [1] 0 
 
  3.12.1  AUC 
 
 
 
 
 
 
 
 
 
 
  
 test 
 Difference 
 p_value 
 Significance 
 
 
 
 
 c2 
 WT/WT vs Δ HEL/WT 
 948.3586 
 0.3963 
 FALSE 
 
 
 c3 
 Female vs Male 
 -607.6252 
 0.8253 
 FALSE 
 
 
 c4 
 Female WT/WT vs Female Δ HEL/WT 
 2322.3928 
 0.0325 
 TRUE 
 
 
 c5 
 Male WT/WT vs Male Δ HEL/WT 
 -425.6755 
 0.9604 
 FALSE 
 
 
 c6 
 Female Δ HEL/WT vs Male Δ HEL/WT 
 -1981.6594 
 0.1629 
 FALSE 
 
 
 
   
    
 
 
 
  3.13  Auditory Brain Stem
Response 
  Number of animals  
   
 
 
 
 STRAIN 
 GENDER 
 count 
 
 
 
 
 WT/WT 
 female 
 8 
 
 
 WT/WT 
 male 
 8 
 
 
 Δ HEL/WT 
 female 
 7 
 
 
 Δ HEL/WT 
 male 
 7 
 
 
 
   
 
  3.13.1  Click-evoked ABR
threshold 
 
 
 
 
 
 
 
 
 
 
  
 test 
 Difference 
 p_value 
 Significance 
 
 
 
 
 c2 
 WT/WT vs Δ HEL/WT 
 0.7397 
 0.9941 
 FALSE 
 
 
 c3 
 Female vs Male 
 2.4166 
 0.8310 
 FALSE 
 
 
 c4 
 Female WT/WT vs Female Δ HEL/WT 
 2.0730 
 0.9570 
 FALSE 
 
 
 c5 
 Male WT/WT vs Male Δ HEL/WT 
 -0.5937 
 0.9989 
 FALSE 
 
 
 c6 
 Female Δ HEL/WT vs Male Δ HEL/WT 
 1.0833 
 0.9938 
 FALSE 
 
 
 
      
 
 
  3.13.2  6kHz-evoked ABR
Threshold 
 
 
 
 
 
 
 
 
 
 
  
 test 
 Difference 
 p_value 
 Significance 
 
 
 
 
 c2 
 WT/WT vs Δ HEL/WT 
 -5.1786 
 0.1808 
 FALSE 
 
 
 c3 
 Female vs Male 
 -6.5179 
 0.0565 
 FALSE 
 
 
 c4 
 Female WT/WT vs Female Δ HEL/WT 
 -2.4107 
 0.9058 
 FALSE 
 
 
 c5 
 Male WT/WT vs Male Δ HEL/WT 
 -7.9464 
 0.1273 
 FALSE 
 
 
 c6 
 Female Δ HEL/WT vs Male Δ HEL/WT 
 -9.2857 
 0.0662 
 FALSE 
 
 
 
      
 
 
  3.13.3  12kHz-evoked ABR
Threshold 
 
 
 
 
 
 
 
 
 
 
  
 test 
 Difference 
 p_value 
 Significance 
 
 
 
 
 c2 
 WT/WT vs Δ HEL/WT 
 7.4754 
 0.6537 
 FALSE 
 
 
 c3 
 Female vs Male 
 -6.2340 
 0.7587 
 FALSE 
 
 
 c4 
 Female WT/WT vs Female Δ HEL/WT 
 10.3399 
 0.6775 
 FALSE 
 
 
 c5 
 Male WT/WT vs Male Δ HEL/WT 
 4.6109 
 0.9534 
 FALSE 
 
 
 c6 
 Female Δ HEL/WT vs Male Δ HEL/WT 
 -9.0985 
 0.7507 
 FALSE 
 
 
 
      
 
 
  3.13.4  18kHz-evoked ABR
Threshold 
 
 
 
 
 
 
 
 
 
 
  
 test 
 Difference 
 p_value 
 Significance 
 
 
 
 
 c2 
 WT/WT vs Δ HEL/WT 
 14.2806 
 0.5406 
 FALSE 
 
 
 c3 
 Female vs Male 
 -11.6980 
 0.6836 
 FALSE 
 
 
 c4 
 Female WT/WT vs Female Δ HEL/WT 
 19.3486 
 0.3441 
 FALSE 
 
 
 c5 
 Male WT/WT vs Male Δ HEL/WT 
 9.2125 
 0.9535 
 FALSE 
 
 
 c6 
 Female Δ HEL/WT vs Male Δ HEL/WT 
 -16.7661 
 0.7024 
 FALSE 
 
 
 
      
 
 
 
  3.14  Body
Composition 
  Number of animals  
   
 
 
 
 STRAIN 
 GENDER 
 count 
 
 
 
 
 WT/WT 
 female 
 8 
 
 
 WT/WT 
 male 
 8 
 
 
 Δ HEL/WT 
 female 
 8 
 
 
 Δ HEL/WT 
 male 
 6 
 
 
 
   
 
  3.14.1  BMC/Body weight
CCP 
 
 
 
 
 
 
 
 
 
 
  
 test 
 Difference 
 p_value 
 Significance 
 
 
 
 
 c2 
 WT/WT vs Δ HEL/WT 
 -2e-04 
 0.0036 
 TRUE 
 
 
 c3 
 Female vs Male 
 4e-04 
 0.0000 
 TRUE 
 
 
 c4 
 Female WT/WT vs Female Δ HEL/WT 
 -2e-04 
 0.0149 
 TRUE 
 
 
 c5 
 Male WT/WT vs Male Δ HEL/WT 
 -2e-04 
 0.1305 
 FALSE 
 
 
 c6 
 Female Δ HEL/WT vs Male Δ HEL/WT 
 5e-04 
 0.0000 
 TRUE 
 
 
 
    
 
 
  3.14.2  Bone Area CCP 
 
 
 
 
 
 
 
 
 
 
  
 test 
 Difference 
 p_value 
 Significance 
 
 
 
 
 c2 
 WT/WT vs Δ HEL/WT 
 13.8142 
 0.9163 
 FALSE 
 
 
 c3 
 Female vs Male 
 -62.8955 
 0.0388 
 TRUE 
 
 
 c4 
 Female WT/WT vs Female Δ HEL/WT 
 5.6458 
 0.9973 
 FALSE 
 
 
 c5 
 Male WT/WT vs Male Δ HEL/WT 
 21.9826 
 0.8965 
 FALSE 
 
 
 c6 
 Female Δ HEL/WT vs Male Δ HEL/WT 
 -54.7271 
 0.3355 
 FALSE 
 
 
 
    
 
 
  3.14.3  Bone Mineral
Content (excluding skull) CCP 
 
 
 
 
 
 
 
 
 
 
  
 test 
 Difference 
 p_value 
 Significance 
 
 
 
 
 c2 
 WT/WT vs Δ HEL/WT 
 -0.0011 
 0.9319 
 FALSE 
 
 
 c3 
 Female vs Male 
 0.0017 
 0.8029 
 FALSE 
 
 
 c4 
 Female WT/WT vs Female Δ HEL/WT 
 -0.0016 
 0.9237 
 FALSE 
 
 
 c5 
 Male WT/WT vs Male Δ HEL/WT 
 -0.0006 
 0.9952 
 FALSE 
 
 
 c6 
 Female Δ HEL/WT vs Male Δ HEL/WT 
 0.0021 
 0.8599 
 FALSE 
 
 
 
    
 
 
  3.14.4  Bone Mineral
Density CCP 
 
 
 
 
 
 
 
 
 
 
  
 test 
 Difference 
 p_value 
 Significance 
 
 
 
 
 c2 
 WT/WT vs Δ HEL/WT 
 -0.0020 
 0.0228 
 TRUE 
 
 
 c3 
 Female vs Male 
 0.0057 
 0.0000 
 TRUE 
 
 
 c4 
 Female WT/WT vs Female Δ HEL/WT 
 -0.0020 
 0.1225 
 FALSE 
 
 
 c5 
 Male WT/WT vs Male Δ HEL/WT 
 -0.0020 
 0.1742 
 FALSE 
 
 
 c6 
 Female Δ HEL/WT vs Male Δ HEL/WT 
 0.0057 
 0.0000 
 TRUE 
 
 
 
    
 
 
  3.14.5  Fat mass CCP 
 
 
 
 
 
 
 
 
 
 
  
 test 
 Difference 
 p_value 
 Significance 
 
 
 
 
 c2 
 WT/WT vs Δ HEL/WT 
 0.2226 
 0.7457 
 FALSE 
 
 
 c3 
 Female vs Male 
 -0.3445 
 0.4236 
 FALSE 
 
 
 c4 
 Female WT/WT vs Female Δ HEL/WT 
 0.3228 
 0.7069 
 FALSE 
 
 
 c5 
 Male WT/WT vs Male Δ HEL/WT 
 0.1224 
 0.9809 
 FALSE 
 
 
 c6 
 Female Δ HEL/WT vs Male Δ HEL/WT 
 -0.4447 
 0.5304 
 FALSE 
 
 
 
    
 
 
  3.14.6  Fat/Body
weight 
 
 
 
 
 
 
 
 
 
 
  
 test 
 Difference 
 p_value 
 Significance 
 
 
 
 
 c2 
 WT/WT vs Δ HEL/WT 
 0.0009 
 0.9989 
 FALSE 
 
 
 c3 
 Female vs Male 
 0.0024 
 0.9779 
 FALSE 
 
 
 c4 
 Female WT/WT vs Female Δ HEL/WT 
 0.0044 
 0.9453 
 FALSE 
 
 
 c5 
 Male WT/WT vs Male Δ HEL/WT 
 -0.0027 
 0.9892 
 FALSE 
 
 
 c6 
 Female Δ HEL/WT vs Male Δ HEL/WT 
 -0.0012 
 0.9990 
 FALSE 
 
 
 
    
 
 
  3.14.7  Lean mass CCP 
 
 
 
 
 
 
 
 
 
 
  
 test 
 Difference 
 p_value 
 Significance 
 
 
 
 
 c2 
 WT/WT vs Δ HEL/WT 
 2.3427 
 0.0067 
 TRUE 
 
 
 c3 
 Female vs Male 
 -5.9169 
 0.0000 
 TRUE 
 
 
 c4 
 Female WT/WT vs Female Δ HEL/WT 
 2.1566 
 0.0900 
 FALSE 
 
 
 c5 
 Male WT/WT vs Male Δ HEL/WT 
 2.5288 
 0.0589 
 FALSE 
 
 
 c6 
 Female Δ HEL/WT vs Male Δ HEL/WT 
 -5.7308 
 0.0000 
 TRUE 
 
 
 
    
 
 
  3.14.8  Lean/Body weight
CCP 
 
 
 
 
 
 
 
 
 
 
  
 test 
 Difference 
 p_value 
 Significance 
 
 
 
 
 c2 
 WT/WT vs Δ HEL/WT 
 0.0090 
 0.6934 
 FALSE 
 
 
 c3 
 Female vs Male 
 -0.0349 
 0.0016 
 TRUE 
 
 
 c4 
 Female WT/WT vs Female Δ HEL/WT 
 0.0120 
 0.7034 
 FALSE 
 
 
 c5 
 Male WT/WT vs Male Δ HEL/WT 
 0.0059 
 0.9585 
 FALSE 
 
 
 c6 
 Female Δ HEL/WT vs Male Δ HEL/WT 
 -0.0379 
 0.0228 
 TRUE 
 
 
 
    
 
 
  3.14.9  relative V
bone 
 
 
 
 
 
 
 
 
 
 
  
 test 
 Difference 
 p_value 
 Significance 
 
 
 
 
 c2 
 WT/WT vs Δ HEL/WT 
 -0.3108 
 0.0037 
 TRUE 
 
 
 c3 
 Female vs Male 
 0.6713 
 0.0000 
 TRUE 
 
 
 c4 
 Female WT/WT vs Female Δ HEL/WT 
 -0.3980 
 0.0059 
 TRUE 
 
 
 c5 
 Male WT/WT vs Male Δ HEL/WT 
 -0.2236 
 0.2411 
 FALSE 
 
 
 c6 
 Female Δ HEL/WT vs Male Δ HEL/WT 
 0.7586 
 0.0000 
 TRUE 
 
 
 
    
 
 
  3.14.10  relative V
fat 
 
 
 
 
 
 
 
 
 
 
  
 test 
 Difference 
 p_value 
 Significance 
 
 
 
 
 c2 
 WT/WT vs Δ HEL/WT 
 0.0141 
 1.0000 
 FALSE 
 
 
 c3 
 Female vs Male 
 0.6374 
 0.8666 
 FALSE 
 
 
 c4 
 Female WT/WT vs Female Δ HEL/WT 
 0.3969 
 0.9844 
 FALSE 
 
 
 c5 
 Male WT/WT vs Male Δ HEL/WT 
 -0.3686 
 0.9899 
 FALSE 
 
 
 c6 
 Female Δ HEL/WT vs Male Δ HEL/WT 
 0.2547 
 0.9966 
 FALSE 
 
 
 
    
 
 
  3.14.11  relative V
lean 
 
 
 
 
 
 
 
 
 
 
  
 test 
 Difference 
 p_value 
 Significance 
 
 
 
 
 c2 
 WT/WT vs Δ HEL/WT 
 0.4627 
 0.9370 
 FALSE 
 
 
 c3 
 Female vs Male 
 -1.4402 
 0.3055 
 FALSE 
 
 
 c4 
 Female WT/WT vs Female Δ HEL/WT 
 0.0167 
 1.0000 
 FALSE 
 
 
 c5 
 Male WT/WT vs Male Δ HEL/WT 
 0.9087 
 0.8648 
 FALSE 
 
 
 c6 
 Female Δ HEL/WT vs Male Δ HEL/WT 
 -0.9941 
 0.8312 
 FALSE 
 
 
 
    
 
 
  3.14.12  Tissue Mineral
Density 
 
 
 
 
 
 
 
 
 
 
  
 test 
 Difference 
 p_value 
 Significance 
 
 
 
 
 c2 
 WT/WT vs Δ HEL/WT 
 -0.0029 
 0.8450 
 FALSE 
 
 
 c3 
 Female vs Male 
 0.0240 
 0.0000 
 TRUE 
 
 
 c4 
 Female WT/WT vs Female Δ HEL/WT 
 0.0004 
 0.9998 
 FALSE 
 
 
 c5 
 Male WT/WT vs Male Δ HEL/WT 
 -0.0063 
 0.6385 
 FALSE 
 
 
 c6 
 Female Δ HEL/WT vs Male Δ HEL/WT 
 0.0206 
 0.0040 
 TRUE 
 
 
 
    
 
 
 
  3.15  Skeleton
morfology 
  Number of animals  
   
 
 
 
 STRAIN 
 GENDER 
 count 
 
 
 
 
 WT/WT 
 female 
 8 
 
 
 WT/WT 
 male 
 8 
 
 
 Δ HEL/WT 
 female 
 8 
 
 
 Δ HEL/WT 
 male 
 6 
 
 
 
   
 
  3.15.1  Tibia length 
 
 
 
 
 
 
 
 
 
 
  
 test 
 Difference 
 p_value 
 Significance 
 
 
 
 
 c2 
 WT/WT vs Δ HEL/WT 
 0.0660 
 0.9509 
 FALSE 
 
 
 c3 
 Female vs Male 
 0.0517 
 0.9745 
 FALSE 
 
 
 c4 
 Female WT/WT vs Female Δ HEL/WT 
 0.0912 
 0.9512 
 FALSE 
 
 
 c5 
 Male WT/WT vs Male Δ HEL/WT 
 0.0408 
 0.9959 
 FALSE 
 
 
 c6 
 Female Δ HEL/WT vs Male Δ HEL/WT 
 0.0265 
 0.9989 
 FALSE 
 
 
 
    
 
 
  3.15.2  Body length 
 
 
 
 
 
 
 
 
 
 
  
 test 
 Difference 
 p_value 
 Significance 
 
 
 
 
 c2 
 WT/WT vs Δ HEL/WT 
 1.2192 
 0.3420 
 FALSE 
 
 
 c3 
 Female vs Male 
 -2.5346 
 0.0044 
 TRUE 
 
 
 c4 
 Female WT/WT vs Female Δ HEL/WT 
 0.8225 
 0.8369 
 FALSE 
 
 
 c5 
 Male WT/WT vs Male Δ HEL/WT 
 1.6158 
 0.4327 
 FALSE 
 
 
 c6 
 Female Δ HEL/WT vs Male Δ HEL/WT 
 -2.1379 
 0.1958 
 FALSE 
 
 
 
    
 
 
  3.15.3  Length of
hindpaw 
 
 
 
 
 
 
 
 
 
 
  
 test 
 Difference 
 p_value 
 Significance 
 
 
 
 
 c2 
 WT/WT vs Δ HEL/WT 
 0.4210 
 0.0679 
 FALSE 
 
 
 c3 
 Female vs Male 
 -0.3287 
 0.1871 
 FALSE 
 
 
 c4 
 Female WT/WT vs Female Δ HEL/WT 
 0.2606 
 0.7023 
 FALSE 
 
 
 c5 
 Male WT/WT vs Male Δ HEL/WT 
 0.5814 
 0.0792 
 FALSE 
 
 
 c6 
 Female Δ HEL/WT vs Male Δ HEL/WT 
 -0.1683 
 0.8957 
 FALSE 
 
 
 
    
 
 
  3.15.4  Length of
cranium 
 
 
 
 
 
 
 
 
 
 
  
 test 
 Difference 
 p_value 
 Significance 
 
 
 
 
 c2 
 WT/WT vs Δ HEL/WT 
 0.6776 
 0.0083 
 TRUE 
 
 
 c3 
 Female vs Male 
 -0.2109 
 0.6962 
 FALSE 
 
 
 c4 
 Female WT/WT vs Female Δ HEL/WT 
 1.0806 
 0.0035 
 TRUE 
 
 
 c5 
 Male WT/WT vs Male Δ HEL/WT 
 0.2746 
 0.7716 
 FALSE 
 
 
 c6 
 Female Δ HEL/WT vs Male Δ HEL/WT 
 -0.6139 
 0.1569 
 FALSE 
 
 
 
     
 
 
  3.15.5  Number of caudal
vertebrae 
   
 
 
 
 
 
 
 
 
 
 
 
 
  
 26:27 
 26:28 
 26:29 
 27:28 
 27:29 
 28:29 
 
 
 
 
 female WT/WT:female Δ HEL/WT 
 1 
 0.9 
 1 
 0.9 
 1 
 0.9 
 
 
 female WT/WT:male WT/WT 
 1 
 0.9 
 1 
 0.9 
 1 
 0.9 
 
 
 female WT/WT:male Δ HEL/WT 
 1 
 1.0 
 1 
 1.0 
 1 
 1.0 
 
 
 female Δ HEL/WT:male WT/WT 
 1 
 1.0 
 1 
 1.0 
 1 
 1.0 
 
 
 female Δ HEL/WT:male Δ HEL/WT 
 1 
 0.9 
 1 
 0.9 
 1 
 1.0 
 
 
 male WT/WT:male Δ HEL/WT 
 1 
 0.9 
 1 
 0.9 
 1 
 1.0 
 
 
 
   
    
 
 
  3.15.6  Number of cervical
vertebrae 
    
    
 
 
  3.15.7  Number of lumbar
vertebrae 
   
 
 
 
  
 female WT/WT 
 female Δ HEL/WT 
 male WT/WT 
 
 
 
 
 female Δ HEL/WT 
 1 
 NA 
 NA 
 
 
 male WT/WT 
 1 
 1 
 NA 
 
 
 male Δ HEL/WT 
 1 
 1 
 1 
 
 
 
   
    
 
 
  3.15.8  Number of pelvic
vertebrae 
   
 
 
 
  
 3:4 
 3:5 
 4:5 
 
 
 
 
 female WT/WT:female Δ HEL/WT 
 1 
 1 
 1 
 
 
 female WT/WT:male WT/WT 
 1 
 1 
 1 
 
 
 female WT/WT:male Δ HEL/WT 
 1 
 1 
 1 
 
 
 female Δ HEL/WT:male WT/WT 
 1 
 1 
 1 
 
 
 female Δ HEL/WT:male Δ HEL/WT 
 1 
 1 
 1 
 
 
 male WT/WT:male Δ HEL/WT 
 1 
 1 
 1 
 
 
 
   
    
 
 
  3.15.9  Number of thoracic
vertebrae 
   
 
 
 
  
 female WT/WT 
 female Δ HEL/WT 
 male WT/WT 
 
 
 
 
 female Δ HEL/WT 
 0.6000 
 NA 
 NA 
 
 
 male WT/WT 
 1.0000 
 0.6000 
 NA 
 
 
 male Δ HEL/WT 
 0.6429 
 0.6965 
 0.6429 
 
 
 
   
    
 
 
  3.15.10  Number of
digits 
    
    
 
 
  3.15.11  Number of digits
in forepaws 
    
    
 
 
  3.15.12  Number of digits
lo hindpaws 
    
    
 
 
  3.15.13  Number of ribs
left 
   
 
 
 
  
 female WT/WT 
 female Δ HEL/WT 
 male WT/WT 
 
 
 
 
 female Δ HEL/WT 
 0.2308 
 NA 
 NA 
 
 
 male WT/WT 
 1.0000 
 0.2308 
 NA 
 
 
 male Δ HEL/WT 
 0.2473 
 0.7524 
 0.2473 
 
 
 
   
    
 
 
  3.15.14  Number of ribs
right 
   
 
 
 
  
 female WT/WT 
 female Δ HEL/WT 
 male WT/WT 
 
 
 
 
 female Δ HEL/WT 
 0.6000 
 NA 
 NA 
 
 
 male WT/WT 
 1.0000 
 0.6000 
 NA 
 
 
 male Δ HEL/WT 
 0.6429 
 0.6965 
 0.6429 
 
 
 
   
     
 
 
  3.15.15 
Brachydactyly 
    
    
 
 
  3.15.16  Caudal
processes 
    
    
 
 
  3.15.17  Cervical
processes 
    
    
 
 
  3.15.18  Clavicle 
    
    
 
 
  3.15.19  Digit
integrity 
    
    
 
 
  3.15.20  Femur 
    
    
 
 
  3.15.21  Fibula 
    
    
 
 
  3.15.22  Fusion of
processes 
    
    
 
 
  3.15.23  Fusion of
ribs 
    
    
 
 
  3.15.24  Fusion of
vertebrae 
    
    
 
 
  3.15.25  Humerus 
    
    
 
 
  3.15.26  Joints 
    
    
 
 
  3.15.27  Kyphosis 
    
    
 
 
  3.15.28  Lordosis 
    
    
 
 
  3.15.29  Lumbar
processes 
   
 
 
 
  
 female WT/WT 
 female Δ HEL/WT 
 male WT/WT 
 
 
 
 
 female Δ HEL/WT 
 0.2378 
 NA 
 NA 
 
 
 male WT/WT 
 1.0000 
 0.1538 
 NA 
 
 
 male Δ HEL/WT 
 0.3671 
 1.0000 
 0.1648 
 
 
 
   
    
 
 
  3.15.30  Mandibles 
    
    
 
 
  3.15.31 
Maxilla/Pre-maxilla 
    
    
 
 
  3.15.32  Missing cranial
rib 
    
    
 
 
  3.15.33  Pelvis 
    
    
 
 
  3.15.34 
Polysyndactylism 
    
    
 
 
  3.15.35  Presence of
baculum bone 
   
 
 
 
  
 female WT/WT 
 female Δ HEL/WT 
 male WT/WT 
 
 
 
 
 female Δ HEL/WT 
 1e+00 
 NA 
 NA 
 
 
 male WT/WT 
 9e-04 
 0.0028 
 NA 
 
 
 male Δ HEL/WT 
 1e-03 
 0.0070 
 1 
 
 
 
   
    
 
 
  3.15.36  Processes on
vertebrae 
   
 
 
 
  
 female WT/WT 
 female Δ HEL/WT 
 male WT/WT 
 
 
 
 
 female Δ HEL/WT 
 0.0811 
 NA 
 NA 
 
 
 male WT/WT 
 1.0000 
 0.042 
 NA 
 
 
 male Δ HEL/WT 
 0.1364 
 1.000 
 0.045 
 
 
 
   
    
 
 
  3.15.37  Quality of enamel
on incisors 
    
    
 
 
  3.15.38  Quality of enamel
on molars 
    
    
 
 
  3.15.39  Radius 
    
    
 
 
  3.15.40  Sacral
processes 
   
 
 
 
  
 female WT/WT 
 female Δ HEL/WT 
 male WT/WT 
 
 
 
 
 female Δ HEL/WT 
 1 
 NA 
 NA 
 
 
 male WT/WT 
 1 
 1 
 NA 
 
 
 male Δ HEL/WT 
 1 
 1 
 1 
 
 
 
   
    
 
 
  3.15.41  Scapulae 
    
    
 
 
  3.15.42  Scoliosis 
   
 
 
 
  
 female WT/WT 
 female Δ HEL/WT 
 male WT/WT 
 
 
 
 
 female Δ HEL/WT 
 1 
 NA 
 NA 
 
 
 male WT/WT 
 1 
 1 
 NA 
 
 
 male Δ HEL/WT 
 1 
 1 
 1 
 
 
 
   
    
 
 
  3.15.43  Shape of
ribcage 
   
 
 
 
  
 female WT/WT 
 female Δ HEL/WT 
 male WT/WT 
 
 
 
 
 female Δ HEL/WT 
 1 
 NA 
 NA 
 
 
 male WT/WT 
 1 
 1 
 NA 
 
 
 male Δ HEL/WT 
 1 
 1 
 1 
 
 
 
   
    
 
 
  3.15.44  Shape of
ribs 
   
 
 
 
  
 female WT/WT 
 female Δ HEL/WT 
 male WT/WT 
 
 
 
 
 female Δ HEL/WT 
 1 
 NA 
 NA 
 
 
 male WT/WT 
 1 
 1 
 NA 
 
 
 male Δ HEL/WT 
 1 
 1 
 1 
 
 
 
   
    
 
 
  3.15.45  Shape of
spine 
   
 
 
 
  
 female WT/WT 
 female Δ HEL/WT 
 male WT/WT 
 
 
 
 
 female Δ HEL/WT 
 1 
 NA 
 NA 
 
 
 male WT/WT 
 1 
 1 
 NA 
 
 
 male Δ HEL/WT 
 1 
 1 
 1 
 
 
 
   
    
 
 
  3.15.46  Shape of
vertebrae 
   
 
 
 
  
 female WT/WT 
 female Δ HEL/WT 
 male WT/WT 
 
 
 
 
 female Δ HEL/WT 
 1 
 NA 
 NA 
 
 
 male WT/WT 
 1 
 1 
 NA 
 
 
 male Δ HEL/WT 
 1 
 1 
 1 
 
 
 
   
    
 
 
  3.15.47  Skull shape 
    
    
 
 
  3.15.48  Syndactylism 
    
    
 
 
  3.15.49  Teeth 
   
 
 
 
  
 female WT/WT 
 female Δ HEL/WT 
 male WT/WT 
 
 
 
 
 female Δ HEL/WT 
 1 
 NA 
 NA 
 
 
 male WT/WT 
 1 
 1 
 NA 
 
 
 male Δ HEL/WT 
 1 
 1 
 1 
 
 
 
   
    
 
 
  3.15.50  Thoracic
processes 
   
 
 
 
  
 female WT/WT 
 female Δ HEL/WT 
 male WT/WT 
 
 
 
 
 female Δ HEL/WT 
 0.7 
 NA 
 NA 
 
 
 male WT/WT 
 1.0 
 0.7 
 NA 
 
 
 male Δ HEL/WT 
 0.7 
 1.0 
 0.7 
 
 
 
   
    
 
 
  3.15.51  Tibia 
    
    
 
 
  3.15.52  Transitional
vertebrae 
   
 
 
 
  
 female WT/WT 
 female Δ HEL/WT 
 male WT/WT 
 
 
 
 
 female Δ HEL/WT 
 0.8706 
 NA 
 NA 
 
 
 male WT/WT 
 1.0000 
 0.8706 
 NA 
 
 
 male Δ HEL/WT 
 1.0000 
 0.8706 
 0.8706 
 
 
 
   
    
 
 
  3.15.53  Ulna 
    
    
 
 
28Y-25534
 
 
 
 
 
Dorso Ventral
 
 
 
 
 
 
 
Dorso Ventral
 
 
 
 
 
 
 
Lateral Orientation
 
 
 
 
 
 
 
Forepaw
 
 
 
 
 
 
 
Skull Lateral Orientation
 
 
 
 
 
 
 
Skull Dorso Ventral Orientation
 
 
 
 
 
 
 
Hind Leg and Hip
 
 
 
 
 
 
 
Hind Leg and Hip
 
 
 
 
 
 
 
uCT image: detail 1
 
 
 
 
28Y-25536
 
 
 
 
 
Dorso Ventral
 
 
 
 
 
 
 
Dorso Ventral
 
 
 
 
 
 
 
Lateral Orientation
 
 
 
 
 
 
 
Forepaw
 
 
 
 
 
 
 
Skull Lateral Orientation
 
 
 
 
 
 
 
Skull Dorso Ventral Orientation
 
 
 
 
 
 
 
Hind Leg and Hip
 
 
 
 
 
 
 
Hind Leg and Hip
 
 
 
 
 
 
 
uCT image: detail 2
 
 
 
 
 
 
 
uCT image: detail 1
 
 
 
 
28Y-25537
 
 
 
 
 
Dorso Ventral
 
 
 
 
 
 
 
Dorso Ventral
 
 
 
 
 
 
 
Lateral Orientation
 
 
 
 
 
 
 
Forepaw
 
 
 
 
 
 
 
Skull Lateral Orientation
 
 
 
 
 
 
 
Skull Dorso Ventral Orientation
 
 
 
 
 
 
 
Hind Leg and Hip
 
 
 
 
 
 
 
Hind Leg and Hip
 
 
 
 
 
 
 
uCT image: detail 1
 
 
 
 
28Y-25576
 
 
 
 
 
Dorso Ventral
 
 
 
 
 
 
 
Dorso Ventral
 
 
 
 
 
 
 
Lateral Orientation
 
 
 
 
 
 
 
Forepaw
 
 
 
 
 
 
 
Skull Lateral Orientation
 
 
 
 
 
 
 
Skull Dorso Ventral Orientation
 
 
 
 
 
 
 
Hind Leg and Hip
 
 
 
 
 
 
 
Hind Leg and Hip
 
 
 
 
 
 
 
uCT image: detail 1
 
 
 
 
28Y-25577
 
 
 
 
 
Dorso Ventral
 
 
 
 
 
 
 
Dorso Ventral
 
 
 
 
 
 
 
Lateral Orientation
 
 
 
 
 
 
 
Forepaw
 
 
 
 
 
 
 
Skull Lateral Orientation
 
 
 
 
 
 
 
Skull Dorso Ventral Orientation
 
 
 
 
 
 
 
Hind Leg and Hip
 
 
 
 
 
 
 
Hind Leg and Hip
 
 
 
 
 
 
 
uCT image: detail 1
 
 
 
 
28Y-25579
 
 
 
 
 
Dorso Ventral
 
 
 
 
 
 
 
Dorso Ventral
 
 
 
 
 
 
 
Lateral Orientation
 
 
 
 
 
 
 
Forepaw
 
 
 
 
 
 
 
Skull Lateral Orientation
 
 
 
 
 
 
 
Skull Dorso Ventral Orientation
 
 
 
 
 
 
 
Hind Leg and Hip
 
 
 
 
 
 
 
Hind Leg and Hip
 
 
 
 
28Y-25580
 
 
 
 
 
Dorso Ventral
 
 
 
 
 
 
 
Dorso Ventral
 
 
 
 
 
 
 
Lateral Orientation
 
 
 
 
 
 
 
Forepaw
 
 
 
 
 
 
 
Skull Lateral Orientation
 
 
 
 
 
 
 
Skull Dorso Ventral Orientation
 
 
 
 
 
 
 
Hind Leg and Hip
 
 
 
 
 
 
 
Hind Leg and Hip
 
 
 
 
 
 
 
uCT image: detail 2
 
 
 
 
 
 
 
uCT image: detail 1
 
 
 
 
28Y-25588
 
 
 
 
 
Dorso Ventral
 
 
 
 
 
 
 
Dorso Ventral
 
 
 
 
 
 
 
Lateral Orientation
 
 
 
 
 
 
 
Forepaw
 
 
 
 
 
 
 
Skull Lateral Orientation
 
 
 
 
 
 
 
Skull Dorso Ventral Orientation
 
 
 
 
 
 
 
Hind Leg and Hip
 
 
 
 
 
 
 
Hind Leg and Hip
 
 
 
 
 
 
 
uCT image: detail 2
 
 
 
 
 
 
 
uCT image: detail 1
 
 
 
 
28Y-25589
 
 
 
 
 
Dorso Ventral
 
 
 
 
 
 
 
Dorso Ventral
 
 
 
 
 
 
 
Lateral Orientation
 
 
 
 
 
 
 
Forepaw
 
 
 
 
 
 
 
Skull Lateral Orientation
 
 
 
 
 
 
 
Skull Dorso Ventral Orientation
 
 
 
 
 
 
 
Hind Leg and Hip
 
 
 
 
 
 
 
Hind Leg and Hip
 
 
 
 
 
 
 
uCT image: detail 1
 
 
 
 
28Y-25590
 
 
 
 
 
Dorso Ventral
 
 
 
 
 
 
 
Dorso Ventral
 
 
 
 
 
 
 
Lateral Orientation
 
 
 
 
 
 
 
Forepaw
 
 
 
 
 
 
 
Skull Lateral Orientation
 
 
 
 
 
 
 
Skull Dorso Ventral Orientation
 
 
 
 
 
 
 
Hind Leg and Hip
 
 
 
 
 
 
 
Hind Leg and Hip
 
 
 
 
 
 
 
uCT image: detail 1
 
 
 
 
28Y-25592
 
 
 
 
 
Dorso Ventral
 
 
 
 
 
 
 
Dorso Ventral
 
 
 
 
 
 
 
Lateral Orientation
 
 
 
 
 
 
 
Forepaw
 
 
 
 
 
 
 
Skull Lateral Orientation
 
 
 
 
 
 
 
Skull Dorso Ventral Orientation
 
 
 
 
 
 
 
Hind Leg and Hip
 
 
 
 
 
 
 
Hind Leg and Hip
 
 
 
 
 
 
 
uCT image: detail 1
 
 
 
 
 
 
  3.16  Vision screen 
  Number of animals  
   
 
 
 
 STRAIN 
 GENDER 
 count 
 
 
 
 
 WT/WT 
 female 
 8 
 
 
 WT/WT 
 male 
 8 
 
 
 Δ HEL/WT 
 female 
 7 
 
 
 Δ HEL/WT 
 male 
 6 
 
 
 
   
 
  3.16.1  Corneal
opacity 
 
 
 
  
 female WT/WT 
 female Δ HEL/WT 
 male WT/WT 
 
 
 
 
 female Δ HEL/WT 
 0.1217 
 NA 
 NA 
 
 
 male WT/WT 
 1.0000 
 0.2378 
 NA 
 
 
 male Δ HEL/WT 
 0.5670 
 0.0280 
 0.3132 
 
 
 
   
   
     
 
 
  3.16.2  Fusion between
cornea and lens 
   
     
 
 
  3.16.3  Lens Opacity 
 
 
 
  
 female WT/WT 
 female Δ HEL/WT 
 male WT/WT 
 
 
 
 
 female Δ HEL/WT 
 1 
 NA 
 NA 
 
 
 male WT/WT 
 1 
 1 
 NA 
 
 
 male Δ HEL/WT 
 1 
 1 
 1 
 
 
 
   
   
     
 
 
  3.16.4  Fusion between
cornea and lens 
    
    
 
 
  3.16.5  Lens 
   
 
 
 
  
 female WT/WT 
 female Δ HEL/WT 
 male WT/WT 
 
 
 
 
 female Δ HEL/WT 
 0.5600 
 NA 
 NA 
 
 
 male WT/WT 
 0.2308 
 0.4231 
 NA 
 
 
 male Δ HEL/WT 
 0.2308 
 0.4231 
 1 
 
 
 
   
    
 
 
  3.16.6  Optic Disc 
    
    
 
 
  3.16.7  Retina
Complete 
    
    
 
 
  3.16.8  Retinal Blood
Vessels 
    
    
 
 
  3.16.9  Retinal Blood
Vessels Pattern 
    
    
 
 
  3.16.10  Retinal Blood
Vessels Structure 
    
    
 
 
 
  3.17  Hematology 
  Number of animals  
   
 
 
 
 STRAIN 
 GENDER 
 count 
 
 
 
 
 WT/WT 
 female 
 8 
 
 
 WT/WT 
 male 
 8 
 
 
 Δ HEL/WT 
 female 
 7 
 
 
 Δ HEL/WT 
 male 
 6 
 
 
 
   
 
  3.17.1  Eosinophil cell
count 
 
 
 
 
 
 
 
 
 
 
  
 test 
 Difference 
 p_value 
 Significance 
 
 
 
 
 c2 
 WT/WT vs Δ HEL/WT 
 0.0074 
 0.9462 
 FALSE 
 
 
 c3 
 Female vs Male 
 -0.0110 
 0.9442 
 FALSE 
 
 
 c4 
 Female WT/WT vs Female Δ HEL/WT 
 -0.0034 
 0.9979 
 FALSE 
 
 
 c5 
 Male WT/WT vs Male Δ HEL/WT 
 0.0182 
 0.7755 
 FALSE 
 
 
 c6 
 Female Δ HEL/WT vs Male Δ HEL/WT 
 -0.0002 
 1.0000 
 FALSE 
 
 
 
      
 
 
  3.17.2  Eosinophil
differential count 
 
 
 
 
 
 
 
 
 
 
  
 test 
 Difference 
 p_value 
 Significance 
 
 
 
 
 c2 
 WT/WT vs Δ HEL/WT 
 -0.0585 
 0.9983 
 FALSE 
 
 
 c3 
 Female vs Male 
 -0.1592 
 0.9899 
 FALSE 
 
 
 c4 
 Female WT/WT vs Female Δ HEL/WT 
 -0.4482 
 0.7978 
 FALSE 
 
 
 c5 
 Male WT/WT vs Male Δ HEL/WT 
 0.3312 
 0.9075 
 FALSE 
 
 
 c6 
 Female Δ HEL/WT vs Male Δ HEL/WT 
 0.2306 
 0.9870 
 FALSE 
 
 
 
      
 
 
  3.17.3  Hematocrit 
 
 
 
 
 
 
 
 
 
 
  
 test 
 Difference 
 p_value 
 Significance 
 
 
 
 
 c2 
 WT/WT vs Δ HEL/WT 
 0.3077 
 0.9788 
 FALSE 
 
 
 c3 
 Female vs Male 
 1.5256 
 0.2171 
 FALSE 
 
 
 c4 
 Female WT/WT vs Female Δ HEL/WT 
 0.3071 
 0.9918 
 FALSE 
 
 
 c5 
 Male WT/WT vs Male Δ HEL/WT 
 0.3083 
 0.9927 
 FALSE 
 
 
 c6 
 Female Δ HEL/WT vs Male Δ HEL/WT 
 1.5262 
 0.5572 
 FALSE 
 
 
 
      
 
 
  3.17.4  Hemoglobin 
 
 
 
 
 
 
 
 
 
 
  
 test 
 Difference 
 p_value 
 Significance 
 
 
 
 
 c2 
 WT/WT vs Δ HEL/WT 
 0.1355 
 0.9378 
 FALSE 
 
 
 c3 
 Female vs Male 
 0.5689 
 0.0971 
 FALSE 
 
 
 c4 
 Female WT/WT vs Female Δ HEL/WT 
 0.1036 
 0.9885 
 FALSE 
 
 
 c5 
 Male WT/WT vs Male Δ HEL/WT 
 0.1674 
 0.9594 
 FALSE 
 
 
 c6 
 Female Δ HEL/WT vs Male Δ HEL/WT 
 0.6008 
 0.3377 
 FALSE 
 
 
 
      
 
 
  3.17.5  Lymphocyte cell
count 
 
 
 
 
 
 
 
 
 
 
  
 test 
 Difference 
 p_value 
 Significance 
 
 
 
 
 c2 
 WT/WT vs Δ HEL/WT 
 0.5021 
 0.6928 
 FALSE 
 
 
 c3 
 Female vs Male 
 -0.4250 
 0.7895 
 FALSE 
 
 
 c4 
 Female WT/WT vs Female Δ HEL/WT 
 1.0409 
 0.3640 
 FALSE 
 
 
 c5 
 Male WT/WT vs Male Δ HEL/WT 
 -0.0367 
 0.9999 
 FALSE 
 
 
 c6 
 Female Δ HEL/WT vs Male Δ HEL/WT 
 -0.9638 
 0.4957 
 FALSE 
 
 
 
      
 
 
  3.17.6  Lymphocyte
differential count 
 
 
 
 
 
 
 
 
 
 
  
 test 
 Difference 
 p_value 
 Significance 
 
 
 
 
 c2 
 WT/WT vs Δ HEL/WT 
 -4.1440 
 0.0736 
 FALSE 
 
 
 c3 
 Female vs Male 
 1.8528 
 0.7211 
 FALSE 
 
 
 c4 
 Female WT/WT vs Female Δ HEL/WT 
 1.4054 
 0.9291 
 FALSE 
 
 
 c5 
 Male WT/WT vs Male Δ HEL/WT 
 -9.6934 
 0.0005 
 TRUE 
 
 
 c6 
 Female Δ HEL/WT vs Male Δ HEL/WT 
 -3.6966 
 0.4826 
 FALSE 
 
 
 
      
 
 
  3.17.7  Mean cell
hemoglobin concentration 
 
 
 
 
 
 
 
 
 
 
  
 test 
 Difference 
 p_value 
 Significance 
 
 
 
 
 c2 
 WT/WT vs Δ HEL/WT 
 0.1003 
 0.9266 
 FALSE 
 
 
 c3 
 Female vs Male 
 0.2164 
 0.5502 
 FALSE 
 
 
 c4 
 Female WT/WT vs Female Δ HEL/WT 
 0.0214 
 0.9997 
 FALSE 
 
 
 c5 
 Male WT/WT vs Male Δ HEL/WT 
 0.1792 
 0.8712 
 FALSE 
 
 
 c6 
 Female Δ HEL/WT vs Male Δ HEL/WT 
 0.2952 
 0.6186 
 FALSE 
 
 
 
      
 
 
  3.17.8  Mean cell
volume 
 
 
 
 
 
 
 
 
 
 
  
 test 
 Difference 
 p_value 
 Significance 
 
 
 
 
 c2 
 WT/WT vs Δ HEL/WT 
 0.4420 
 0.5811 
 FALSE 
 
 
 c3 
 Female vs Male 
 0.7205 
 0.1715 
 FALSE 
 
 
 c4 
 Female WT/WT vs Female Δ HEL/WT 
 0.3839 
 0.8528 
 FALSE 
 
 
 c5 
 Male WT/WT vs Male Δ HEL/WT 
 0.5000 
 0.7492 
 FALSE 
 
 
 c6 
 Female Δ HEL/WT vs Male Δ HEL/WT 
 0.7786 
 0.4371 
 FALSE 
 
 
 
      
 
 
  3.17.9  Mean corpuscular
hemoglobin 
 
 
 
 
 
 
 
 
 
 
  
 test 
 Difference 
 p_value 
 Significance 
 
 
 
 
 c2 
 WT/WT vs Δ HEL/WT 
 0.1970 
 0.3485 
 FALSE 
 
 
 c3 
 Female vs Male 
 0.3113 
 0.0497 
 TRUE 
 
 
 c4 
 Female WT/WT vs Female Δ HEL/WT 
 0.1607 
 0.7578 
 FALSE 
 
 
 c5 
 Male WT/WT vs Male Δ HEL/WT 
 0.2333 
 0.5227 
 FALSE 
 
 
 c6 
 Female Δ HEL/WT vs Male Δ HEL/WT 
 0.3476 
 0.2067 
 FALSE 
 
 
 
      
 
 
  3.17.10  Mean platelet
volume 
 
 
 
 
 
 
 
 
 
 
  
 test 
 Difference 
 p_value 
 Significance 
 
 
 
 
 c2 
 WT/WT vs Δ HEL/WT 
 -0.0581 
 0.7772 
 FALSE 
 
 
 c3 
 Female vs Male 
 0.1293 
 0.2916 
 FALSE 
 
 
 c4 
 Female WT/WT vs Female Δ HEL/WT 
 -0.0807 
 0.7768 
 FALSE 
 
 
 c5 
 Male WT/WT vs Male Δ HEL/WT 
 -0.0354 
 0.9768 
 FALSE 
 
 
 c6 
 Female Δ HEL/WT vs Male Δ HEL/WT 
 0.1520 
 0.4470 
 FALSE 
 
 
 
      
 
 
  3.17.11  Monocyte cell
count 
 
 
 
 
 
 
 
 
 
 
  
 test 
 Difference 
 p_value 
 Significance 
 
 
 
 
 c2 
 WT/WT vs Δ HEL/WT 
 0.0617 
 0.2470 
 FALSE 
 
 
 c3 
 Female vs Male 
 -0.0692 
 0.1614 
 FALSE 
 
 
 c4 
 Female WT/WT vs Female Δ HEL/WT 
 0.0296 
 0.9134 
 FALSE 
 
 
 c5 
 Male WT/WT vs Male Δ HEL/WT 
 0.0938 
 0.2070 
 FALSE 
 
 
 c6 
 Female Δ HEL/WT vs Male Δ HEL/WT 
 -0.0371 
 0.8705 
 FALSE 
 
 
 
      
 
 
  3.17.12  Monocyte
differential count 
 
 
 
 
 
 
 
 
 
 
  
 test 
 Difference 
 p_value 
 Significance 
 
 
 
 
 c2 
 WT/WT vs Δ HEL/WT 
 0.2521 
 0.9387 
 FALSE 
 
 
 c3 
 Female vs Male 
 -0.5979 
 0.5262 
 FALSE 
 
 
 c4 
 Female WT/WT vs Female Δ HEL/WT 
 -0.4125 
 0.9036 
 FALSE 
 
 
 c5 
 Male WT/WT vs Male Δ HEL/WT 
 0.9167 
 0.4747 
 FALSE 
 
 
 c6 
 Female Δ HEL/WT vs Male Δ HEL/WT 
 0.0667 
 0.9996 
 FALSE 
 
 
 
      
 
 
  3.17.13  Neutrophil cell
count 
 
 
 
 
 
 
 
 
 
 
  
 test 
 Difference 
 p_value 
 Significance 
 
 
 
 
 c2 
 WT/WT vs Δ HEL/WT 
 0.3410 
 0.0009 
 TRUE 
 
 
 c3 
 Female vs Male 
 -0.1514 
 0.3303 
 FALSE 
 
 
 c4 
 Female WT/WT vs Female Δ HEL/WT 
 0.1398 
 0.6669 
 FALSE 
 
 
 c5 
 Male WT/WT vs Male Δ HEL/WT 
 0.5421 
 0.0002 
 TRUE 
 
 
 c6 
 Female Δ HEL/WT vs Male Δ HEL/WT 
 0.0498 
 0.9814 
 FALSE 
 
 
 
      
 
 
  3.17.14  Neutrophil
differential count 
 
 
 
 
 
 
 
 
 
 
  
 test 
 Difference 
 p_value 
 Significance 
 
 
 
 
 c2 
 WT/WT vs Δ HEL/WT 
 3.7905 
 0.0640 
 FALSE 
 
 
 c3 
 Female vs Male 
 -1.0488 
 0.8954 
 FALSE 
 
 
 c4 
 Female WT/WT vs Female Δ HEL/WT 
 -0.7607 
 0.9827 
 FALSE 
 
 
 c5 
 Male WT/WT vs Male Δ HEL/WT 
 8.3417 
 0.0008 
 TRUE 
 
 
 c6 
 Female Δ HEL/WT vs Male Δ HEL/WT 
 3.5024 
 0.4005 
 FALSE 
 
 
 
      
 
 
  3.17.15  Platelet
count 
 
 
 
 
 
 
 
 
 
 
  
 test 
 Difference 
 p_value 
 Significance 
 
 
 
 
 c2 
 WT/WT vs Δ HEL/WT 
 -2.1310 
 0.9998 
 FALSE 
 
 
 c3 
 Female vs Male 
 -78.0774 
 0.0338 
 TRUE 
 
 
 c4 
 Female WT/WT vs Female Δ HEL/WT 
 -3.3036 
 0.9998 
 FALSE 
 
 
 c5 
 Male WT/WT vs Male Δ HEL/WT 
 -0.9583 
 1.0000 
 FALSE 
 
 
 c6 
 Female Δ HEL/WT vs Male Δ HEL/WT 
 -76.9048 
 0.2617 
 FALSE 
 
 
 
      
 
 
  3.17.16  Platelet
crit 
 
 
 
 
 
 
 
 
 
 
  
 test 
 Difference 
 p_value 
 Significance 
 
 
 
 
 c2 
 WT/WT vs Δ HEL/WT 
 -0.0455 
 0.9858 
 FALSE 
 
 
 c3 
 Female vs Male 
 -0.2653 
 0.1993 
 FALSE 
 
 
 c4 
 Female WT/WT vs Female Δ HEL/WT 
 -0.0539 
 0.9910 
 FALSE 
 
 
 c5 
 Male WT/WT vs Male Δ HEL/WT 
 -0.0371 
 0.9974 
 FALSE 
 
 
 c6 
 Female Δ HEL/WT vs Male Δ HEL/WT 
 -0.2569 
 0.5646 
 FALSE 
 
 
 
      
 
 
  3.17.17  Platelet
distribution width 
 
 
 
 
 
 
 
 
 
 
  
 test 
 Difference 
 p_value 
 Significance 
 
 
 
 
 c2 
 WT/WT vs Δ HEL/WT 
 -0.0295 
 0.9707 
 FALSE 
 
 
 c3 
 Female vs Male 
 0.1420 
 0.1559 
 FALSE 
 
 
 c4 
 Female WT/WT vs Female Δ HEL/WT 
 -0.0089 
 0.9997 
 FALSE 
 
 
 c5 
 Male WT/WT vs Male Δ HEL/WT 
 -0.0500 
 0.9538 
 FALSE 
 
 
 c6 
 Female Δ HEL/WT vs Male Δ HEL/WT 
 0.1214 
 0.6138 
 FALSE 
 
 
 
      
 
 
  3.17.18  Red blood cell
count 
 
 
 
 
 
 
 
 
 
 
  
 test 
 Difference 
 p_value 
 Significance 
 
 
 
 
 c2 
 WT/WT vs Δ HEL/WT 
 -0.0189 
 0.9993 
 FALSE 
 
 
 c3 
 Female vs Male 
 0.1714 
 0.6647 
 FALSE 
 
 
 c4 
 Female WT/WT vs Female Δ HEL/WT 
 -0.0154 
 0.9999 
 FALSE 
 
 
 c5 
 Male WT/WT vs Male Δ HEL/WT 
 -0.0225 
 0.9996 
 FALSE 
 
 
 c6 
 Female Δ HEL/WT vs Male Δ HEL/WT 
 0.1679 
 0.8749 
 FALSE 
 
 
 
      
 
 
  3.17.19  Red blood cell
distribution width 
 
 
 
 
 
 
 
 
 
 
  
 test 
 Difference 
 p_value 
 Significance 
 
 
 
 
 c2 
 WT/WT vs Δ HEL/WT 
 -0.5701 
 0.0320 
 TRUE 
 
 
 c3 
 Female vs Male 
 0.1930 
 0.9189 
 FALSE 
 
 
 c4 
 Female WT/WT vs Female Δ HEL/WT 
 -0.3155 
 0.6805 
 FALSE 
 
 
 c5 
 Male WT/WT vs Male Δ HEL/WT 
 -0.8247 
 0.0265 
 TRUE 
 
 
 c6 
 Female Δ HEL/WT vs Male Δ HEL/WT 
 -0.0616 
 0.9987 
 FALSE 
 
 
 
      
 
 
  3.17.20  White blood cell
count 
 
 
 
 
 
 
 
 
 
 
  
 test 
 Difference 
 p_value 
 Significance 
 
 
 
 
 c2 
 WT/WT vs Δ HEL/WT 
 0.9212 
 0.3082 
 FALSE 
 
 
 c3 
 Female vs Male 
 -0.6608 
 0.5944 
 FALSE 
 
 
 c4 
 Female WT/WT vs Female Δ HEL/WT 
 1.2195 
 0.3469 
 FALSE 
 
 
 c5 
 Male WT/WT vs Male Δ HEL/WT 
 0.6229 
 0.8443 
 FALSE 
 
 
 c6 
 Female Δ HEL/WT vs Male Δ HEL/WT 
 -0.9590 
 0.6135 
 FALSE 
 
 
 
      
 
 
 
  3.18  Biochemistry 
  Number of animals  
   
 
 
 
 STRAIN 
 GENDER 
 count 
 
 
 
 
 WT/WT 
 female 
 8 
 
 
 WT/WT 
 male 
 8 
 
 
 Δ HEL/WT 
 female 
 7 
 
 
 Δ HEL/WT 
 male 
 6 
 
 
 
   
 
  3.18.1  Alanine
aminotransferase 
 
 
 
 
 
 
 
 
 
 
  
 test 
 Difference 
 p_value 
 Significance 
 
 
 
 
 c2 
 WT/WT vs Δ HEL/WT 
 -4.6461 
 0.8817 
 FALSE 
 
 
 c3 
 Female vs Male 
 -22.1320 
 0.1035 
 FALSE 
 
 
 c4 
 Female WT/WT vs Female Δ HEL/WT 
 1.0388 
 0.9994 
 FALSE 
 
 
 c5 
 Male WT/WT vs Male Δ HEL/WT 
 -10.3310 
 0.6559 
 FALSE 
 
 
 c6 
 Female Δ HEL/WT vs Male Δ HEL/WT 
 -27.8168 
 0.1333 
 FALSE 
 
 
 
     
 
 
  3.18.2  Albumin 
 
 
 
 
 
 
 
 
 
 
  
 test 
 Difference 
 p_value 
 Significance 
 
 
 
 
 c2 
 WT/WT vs Δ HEL/WT 
 -0.8243 
 0.1999 
 FALSE 
 
 
 c3 
 Female vs Male 
 1.5684 
 0.0044 
 TRUE 
 
 
 c4 
 Female WT/WT vs Female Δ HEL/WT 
 -0.4333 
 0.8716 
 FALSE 
 
 
 c5 
 Male WT/WT vs Male Δ HEL/WT 
 -1.2154 
 0.1794 
 FALSE 
 
 
 c6 
 Female Δ HEL/WT vs Male Δ HEL/WT 
 1.1773 
 0.2766 
 FALSE 
 
 
 
     
 
 
  3.18.3  Alkaline
phosphatase 
 
 
 
 
 
 
 
 
 
 
  
 test 
 Difference 
 p_value 
 Significance 
 
 
 
 
 c2 
 WT/WT vs Δ HEL/WT 
 10.7298 
 0.1809 
 FALSE 
 
 
 c3 
 Female vs Male 
 3.1120 
 0.9844 
 FALSE 
 
 
 c4 
 Female WT/WT vs Female Δ HEL/WT 
 22.1661 
 0.0198 
 TRUE 
 
 
 c5 
 Male WT/WT vs Male Δ HEL/WT 
 -0.7064 
 0.9997 
 FALSE 
 
 
 c6 
 Female Δ HEL/WT vs Male Δ HEL/WT 
 -8.3242 
 0.8839 
 FALSE 
 
 
 
     
 
 
  3.18.4  Aspartate
aminotransferase 
 
 
 
 
 
 
 
 
 
 
  
 test 
 Difference 
 p_value 
 Significance 
 
 
 
 
 c2 
 WT/WT vs Δ HEL/WT 
 -1.7798 
 0.9688 
 FALSE 
 
 
 c3 
 Female vs Male 
 -0.6369 
 0.9985 
 FALSE 
 
 
 c4 
 Female WT/WT vs Female Δ HEL/WT 
 -0.8929 
 0.9984 
 FALSE 
 
 
 c5 
 Male WT/WT vs Male Δ HEL/WT 
 -2.6667 
 0.9654 
 FALSE 
 
 
 c6 
 Female Δ HEL/WT vs Male Δ HEL/WT 
 -1.5238 
 0.9937 
 FALSE 
 
 
 
     
 
 
  3.18.5  Calcium 
 
 
 
 
 
 
 
 
 
 
  
 test 
 Difference 
 p_value 
 Significance 
 
 
 
 
 c2 
 WT/WT vs Δ HEL/WT 
 -0.0088 
 0.9993 
 FALSE 
 
 
 c3 
 Female vs Male 
 0.0955 
 0.5249 
 FALSE 
 
 
 c4 
 Female WT/WT vs Female Δ HEL/WT 
 -0.0343 
 0.9844 
 FALSE 
 
 
 c5 
 Male WT/WT vs Male Δ HEL/WT 
 0.0167 
 0.9984 
 FALSE 
 
 
 c6 
 Female Δ HEL/WT vs Male Δ HEL/WT 
 0.1210 
 0.6514 
 FALSE 
 
 
 
     
 
 
  3.18.6  Chloride 
 
 
 
 
 
 
 
 
 
 
  
 test 
 Difference 
 p_value 
 Significance 
 
 
 
 
 c2 
 WT/WT vs Δ HEL/WT 
 -0.5636 
 0.6617 
 FALSE 
 
 
 c3 
 Female vs Male 
 1.5770 
 0.0340 
 TRUE 
 
 
 c4 
 Female WT/WT vs Female Δ HEL/WT 
 -0.2912 
 0.9733 
 FALSE 
 
 
 c5 
 Male WT/WT vs Male Δ HEL/WT 
 -0.8361 
 0.6344 
 FALSE 
 
 
 c6 
 Female Δ HEL/WT vs Male Δ HEL/WT 
 1.3045 
 0.3685 
 FALSE 
 
 
 
     
 
 
  3.18.7  Creatinine 
 
 
 
 
 
 
 
 
 
 
  
 test 
 Difference 
 p_value 
 Significance 
 
 
 
 
 c2 
 WT/WT vs Δ HEL/WT 
 0.0003 
 0.9998 
 FALSE 
 
 
 c3 
 Female vs Male 
 0.0188 
 0.0000 
 TRUE 
 
 
 c4 
 Female WT/WT vs Female Δ HEL/WT 
 0.0009 
 0.9978 
 FALSE 
 
 
 c5 
 Male WT/WT vs Male Δ HEL/WT 
 -0.0003 
 0.9999 
 FALSE 
 
 
 c6 
 Female Δ HEL/WT vs Male Δ HEL/WT 
 0.0182 
 0.0047 
 TRUE 
 
 
 
     
 
 
  3.18.8  Glucose 
 
 
 
 
 
 
 
 
 
 
  
 test 
 Difference 
 p_value 
 Significance 
 
 
 
 
 c2 
 WT/WT vs Δ HEL/WT 
 -3.5371 
 0.9544 
 FALSE 
 
 
 c3 
 Female vs Male 
 -23.8878 
 0.0038 
 TRUE 
 
 
 c4 
 Female WT/WT vs Female Δ HEL/WT 
 -2.5772 
 0.9928 
 FALSE 
 
 
 c5 
 Male WT/WT vs Male Δ HEL/WT 
 -4.4970 
 0.9682 
 FALSE 
 
 
 c6 
 Female Δ HEL/WT vs Male Δ HEL/WT 
 -24.8477 
 0.0801 
 FALSE 
 
 
 
     
 
 
  3.18.9 
HDL-cholesterol 
 
 
 
 
 
 
 
 
 
 
  
 test 
 Difference 
 p_value 
 Significance 
 
 
 
 
 c2 
 WT/WT vs Δ HEL/WT 
 4.7982 
 0.4993 
 FALSE 
 
 
 c3 
 Female vs Male 
 -27.8113 
 0.0000 
 TRUE 
 
 
 c4 
 Female WT/WT vs Female Δ HEL/WT 
 7.2660 
 0.4307 
 FALSE 
 
 
 c5 
 Male WT/WT vs Male Δ HEL/WT 
 2.3304 
 0.9616 
 FALSE 
 
 
 c6 
 Female Δ HEL/WT vs Male Δ HEL/WT 
 -30.2791 
 0.0000 
 TRUE 
 
 
 
     
 
 
  3.18.10  Iron 
 
 
 
 
 
 
 
 
 
 
  
 test 
 Difference 
 p_value 
 Significance 
 
 
 
 
 c2 
 WT/WT vs Δ HEL/WT 
 -0.0223 
 0.0905 
 FALSE 
 
 
 c3 
 Female vs Male 
 0.0126 
 0.8192 
 FALSE 
 
 
 c4 
 Female WT/WT vs Female Δ HEL/WT 
 -0.0317 
 0.0839 
 FALSE 
 
 
 c5 
 Male WT/WT vs Male Δ HEL/WT 
 -0.0128 
 0.7598 
 FALSE 
 
 
 c6 
 Female Δ HEL/WT vs Male Δ HEL/WT 
 0.0221 
 0.6548 
 FALSE 
 
 
 
     
 
 
  3.18.11  Phosphate 
 
 
 
 
 
 
 
 
 
 
  
 test 
 Difference 
 p_value 
 Significance 
 
 
 
 
 c2 
 WT/WT vs Δ HEL/WT 
 0.1518 
 0.9303 
 FALSE 
 
 
 c3 
 Female vs Male 
 1.0721 
 0.0814 
 FALSE 
 
 
 c4 
 Female WT/WT vs Female Δ HEL/WT 
 0.0308 
 0.9998 
 FALSE 
 
 
 c5 
 Male WT/WT vs Male Δ HEL/WT 
 0.2727 
 0.8678 
 FALSE 
 
 
 c6 
 Female Δ HEL/WT vs Male Δ HEL/WT 
 1.1931 
 0.1659 
 FALSE 
 
 
 
     
 
 
  3.18.12  Potassium 
 
 
 
 
 
 
 
 
 
 
  
 test 
 Difference 
 p_value 
 Significance 
 
 
 
 
 c2 
 WT/WT vs Δ HEL/WT 
 0.4311 
 0.4775 
 FALSE 
 
 
 c3 
 Female vs Male 
 -1.4582 
 0.0000 
 TRUE 
 
 
 c4 
 Female WT/WT vs Female Δ HEL/WT 
 0.1205 
 0.9912 
 FALSE 
 
 
 c5 
 Male WT/WT vs Male Δ HEL/WT 
 0.7417 
 0.3228 
 FALSE 
 
 
 c6 
 Female Δ HEL/WT vs Male Δ HEL/WT 
 -1.1476 
 0.0566 
 FALSE 
 
 
 
     
 
 
  3.18.13  Sodium 
 
 
 
 
 
 
 
 
 
 
  
 test 
 Difference 
 p_value 
 Significance 
 
 
 
 
 c2 
 WT/WT vs Δ HEL/WT 
 -1.1190 
 0.2090 
 FALSE 
 
 
 c3 
 Female vs Male 
 0.2024 
 0.9841 
 FALSE 
 
 
 c4 
 Female WT/WT vs Female Δ HEL/WT 
 -0.8214 
 0.7213 
 FALSE 
 
 
 c5 
 Male WT/WT vs Male Δ HEL/WT 
 -1.4167 
 0.3169 
 FALSE 
 
 
 c6 
 Female Δ HEL/WT vs Male Δ HEL/WT 
 -0.0952 
 0.9995 
 FALSE 
 
 
 
     
 
 
  3.18.14  Total
bilirubin 
 
 
 
 
 
 
 
 
 
 
  
 test 
 Difference 
 p_value 
 Significance 
 
 
 
 
 c2 
 WT/WT vs Δ HEL/WT 
 -0.0092 
 0.8957 
 FALSE 
 
 
 c3 
 Female vs Male 
 -0.0467 
 0.0596 
 FALSE 
 
 
 c4 
 Female WT/WT vs Female Δ HEL/WT 
 -0.0098 
 0.9496 
 FALSE 
 
 
 c5 
 Male WT/WT vs Male Δ HEL/WT 
 -0.0085 
 0.9666 
 FALSE 
 
 
 c6 
 Female Δ HEL/WT vs Male Δ HEL/WT 
 -0.0461 
 0.2465 
 FALSE 
 
 
 
     
 
 
  3.18.15  Total
cholesterol 
 
 
 
 
 
 
 
 
 
 
  
 test 
 Difference 
 p_value 
 Significance 
 
 
 
 
 c2 
 WT/WT vs Δ HEL/WT 
 6.0794 
 0.6482 
 FALSE 
 
 
 c3 
 Female vs Male 
 -35.7339 
 0.0001 
 TRUE 
 
 
 c4 
 Female WT/WT vs Female Δ HEL/WT 
 10.9660 
 0.4441 
 FALSE 
 
 
 c5 
 Male WT/WT vs Male Δ HEL/WT 
 1.1928 
 0.9984 
 FALSE 
 
 
 c6 
 Female Δ HEL/WT vs Male Δ HEL/WT 
 -40.6205 
 0.0013 
 TRUE 
 
 
 
     
 
 
  3.18.16  Total
protein 
 
 
 
 
 
 
 
 
 
 
  
 test 
 Difference 
 p_value 
 Significance 
 
 
 
 
 c2 
 WT/WT vs Δ HEL/WT 
 -1.0705 
 0.3106 
 FALSE 
 
 
 c3 
 Female vs Male 
 0.8678 
 0.7898 
 FALSE 
 
 
 c4 
 Female WT/WT vs Female Δ HEL/WT 
 -1.0104 
 0.6430 
 FALSE 
 
 
 c5 
 Male WT/WT vs Male Δ HEL/WT 
 -1.1305 
 0.5604 
 FALSE 
 
 
 c6 
 Female Δ HEL/WT vs Male Δ HEL/WT 
 0.8077 
 0.9128 
 FALSE 
 
 
 
     
 
 
  3.18.17 
Triglycerides 
 
 
 
 
 
 
 
 
 
 
  
 test 
 Difference 
 p_value 
 Significance 
 
 
 
 
 c2 
 WT/WT vs Δ HEL/WT 
 9.3123 
 0.8387 
 FALSE 
 
 
 c3 
 Female vs Male 
 -19.3012 
 0.6810 
 FALSE 
 
 
 c4 
 Female WT/WT vs Female Δ HEL/WT 
 14.9870 
 0.7763 
 FALSE 
 
 
 c5 
 Male WT/WT vs Male Δ HEL/WT 
 3.6376 
 0.9956 
 FALSE 
 
 
 c6 
 Female Δ HEL/WT vs Male Δ HEL/WT 
 -24.9758 
 0.6931 
 FALSE 
 
 
 
     
 
 
  3.18.18  Urea (Blood Urea
Nitrogen - BUN) 
 
 
 
 
 
 
 
 
 
 
  
 test 
 Difference 
 p_value 
 Significance 
 
 
 
 
 c2 
 WT/WT vs Δ HEL/WT 
 1.7526 
 0.4573 
 FALSE 
 
 
 c3 
 Female vs Male 
 -10.2962 
 0.0000 
 TRUE 
 
 
 c4 
 Female WT/WT vs Female Δ HEL/WT 
 1.5923 
 0.7751 
 FALSE 
 
 
 c5 
 Male WT/WT vs Male Δ HEL/WT 
 1.9130 
 0.6611 
 FALSE 
 
 
 c6 
 Female Δ HEL/WT vs Male Δ HEL/WT 
 -10.1359 
 0.0012 
 TRUE 
 
 
 
     
 
 
 
  3.19  Organ weight 
  Number of animals  
   
 
 
 
 STRAIN 
 GENDER 
 count 
 
 
 
 
 WT/WT 
 female 
 8 
 
 
 WT/WT 
 male 
 8 
 
 
 Δ HEL/WT 
 female 
 7 
 
 
 Δ HEL/WT 
 male 
 6 
 
 
 
   
 
  3.19.1  Heart weight 
 
 
 
 
 
 
 
 
 
 
  
 test 
 Difference 
 p_value 
 Significance 
 
 
 
 
 c2 
 WT/WT vs Δ HEL/WT 
 15.5833 
 0.2437 
 FALSE 
 
 
 c3 
 Female vs Male 
 -54.1667 
 0.0000 
 TRUE 
 
 
 c4 
 Female WT/WT vs Female Δ HEL/WT 
 24.2500 
 0.1570 
 FALSE 
 
 
 c5 
 Male WT/WT vs Male Δ HEL/WT 
 6.9167 
 0.9370 
 FALSE 
 
 
 c6 
 Female Δ HEL/WT vs Male Δ HEL/WT 
 -62.8333 
 0.0000 
 TRUE 
 
 
 
     
 
 
  3.19.2  Heart weight
normalised against body weight 
 
 
 
 
 
 
 
 
 
 
  
 test 
 Difference 
 p_value 
 Significance 
 
 
 
 
 c2 
 WT/WT vs Δ HEL/WT 
 -2e-04 
 0.8668 
 FALSE 
 
 
 c3 
 Female vs Male 
 -6e-04 
 0.0841 
 FALSE 
 
 
 c4 
 Female WT/WT vs Female Δ HEL/WT 
 1e-04 
 0.9921 
 FALSE 
 
 
 c5 
 Male WT/WT vs Male Δ HEL/WT 
 -5e-04 
 0.5438 
 FALSE 
 
 
 c6 
 Female Δ HEL/WT vs Male Δ HEL/WT 
 -9e-04 
 0.0867 
 FALSE 
 
 
 
     
 
 
  3.19.3  Tibia length 
 
 
 
 
 
 
 
 
 
 
  
 test 
 Difference 
 p_value 
 Significance 
 
 
 
 
 c2 
 WT/WT vs Δ HEL/WT 
 0.8542 
 0.1772 
 FALSE 
 
 
 c3 
 Female vs Male 
 -0.3958 
 0.7739 
 FALSE 
 
 
 c4 
 Female WT/WT vs Female Δ HEL/WT 
 0.6250 
 0.6957 
 FALSE 
 
 
 c5 
 Male WT/WT vs Male Δ HEL/WT 
 1.0833 
 0.2782 
 FALSE 
 
 
 c6 
 Female Δ HEL/WT vs Male Δ HEL/WT 
 -0.1667 
 0.9929 
 FALSE 
 
 
 
     
 
 
  3.19.4  Heart weight
normalised against tibia length 
 
 
 
 
 
 
 
 
 
 
  
 test 
 Difference 
 p_value 
 Significance 
 
 
 
 
 c2 
 WT/WT vs Δ HEL/WT 
 0.3676 
 0.7997 
 FALSE 
 
 
 c3 
 Female vs Male 
 -2.3860 
 0.0000 
 TRUE 
 
 
 c4 
 Female WT/WT vs Female Δ HEL/WT 
 0.8863 
 0.3962 
 FALSE 
 
 
 c5 
 Male WT/WT vs Male Δ HEL/WT 
 -0.1512 
 0.9938 
 FALSE 
 
 
 c6 
 Female Δ HEL/WT vs Male Δ HEL/WT 
 -2.9048 
 0.0000 
 TRUE 
 
 
 
     
 
 
  3.19.5  Body weight 
 
 
 
 
 
 
 
 
 
 
  
 test 
 Difference 
 p_value 
 Significance 
 
 
 
 
 c2 
 WT/WT vs Δ HEL/WT 
 3.7634 
 0.0000 
 TRUE 
 
 
 c3 
 Female vs Male 
 -5.9259 
 0.0000 
 TRUE 
 
 
 c4 
 Female WT/WT vs Female Δ HEL/WT 
 3.5518 
 0.0060 
 TRUE 
 
 
 c5 
 Male WT/WT vs Male Δ HEL/WT 
 3.9750 
 0.0028 
 TRUE 
 
 
 c6 
 Female Δ HEL/WT vs Male Δ HEL/WT 
 -5.7143 
 0.0000 
 TRUE 
 
 
 
     
 
 
  3.19.6  Left kidney
weight 
 
 
 
 
 
 
 
 
 
 
  
 test 
 Difference 
 p_value 
 Significance 
 
 
 
 
 c2 
 WT/WT vs Δ HEL/WT 
 0.0460 
 0e+00 
 TRUE 
 
 
 c3 
 Female vs Male 
 -0.1170 
 0e+00 
 TRUE 
 
 
 c4 
 Female WT/WT vs Female Δ HEL/WT 
 0.0397 
 3e-04 
 TRUE 
 
 
 c5 
 Male WT/WT vs Male Δ HEL/WT 
 0.0524 
 0e+00 
 TRUE 
 
 
 c6 
 Female Δ HEL/WT vs Male Δ HEL/WT 
 -0.1106 
 0e+00 
 TRUE 
 
 
 
     
 
 
  3.19.7  Liver weight 
 
 
 
 
 
 
 
 
 
 
  
 test 
 Difference 
 p_value 
 Significance 
 
 
 
 
 c2 
 WT/WT vs Δ HEL/WT 
 0.1684 
 0.0671 
 FALSE 
 
 
 c3 
 Female vs Male 
 -0.3739 
 0.0001 
 TRUE 
 
 
 c4 
 Female WT/WT vs Female Δ HEL/WT 
 0.2072 
 0.1282 
 FALSE 
 
 
 c5 
 Male WT/WT vs Male Δ HEL/WT 
 0.1296 
 0.5243 
 FALSE 
 
 
 c6 
 Female Δ HEL/WT vs Male Δ HEL/WT 
 -0.4127 
 0.0037 
 TRUE 
 
 
 
     
 
 
  3.19.8  Lung weight 
 
 
 
 
 
 
 
 
 
 
  
 test 
 Difference 
 p_value 
 Significance 
 
 
 
 
 c2 
 WT/WT vs Δ HEL/WT 
 0.0268 
 0.2309 
 FALSE 
 
 
 c3 
 Female vs Male 
 -0.0254 
 0.2751 
 FALSE 
 
 
 c4 
 Female WT/WT vs Female Δ HEL/WT 
 0.0095 
 0.9598 
 FALSE 
 
 
 c5 
 Male WT/WT vs Male Δ HEL/WT 
 0.0440 
 0.1387 
 FALSE 
 
 
 c6 
 Female Δ HEL/WT vs Male Δ HEL/WT 
 -0.0081 
 0.9793 
 FALSE 
 
 
 
     
 
 
  3.19.9  Right kidney
weight 
 
 
 
 
 
 
 
 
 
 
  
 test 
 Difference 
 p_value 
 Significance 
 
 
 
 
 c2 
 WT/WT vs Δ HEL/WT 
 0.0424 
 0.0000 
 TRUE 
 
 
 c3 
 Female vs Male 
 -0.1244 
 0.0000 
 TRUE 
 
 
 c4 
 Female WT/WT vs Female Δ HEL/WT 
 0.0363 
 0.0085 
 TRUE 
 
 
 c5 
 Male WT/WT vs Male Δ HEL/WT 
 0.0485 
 0.0003 
 TRUE 
 
 
 c6 
 Female Δ HEL/WT vs Male Δ HEL/WT 
 -0.1183 
 0.0000 
 TRUE 
 
 
 
     
 
 
  3.19.10  Spleen
weight 
 
 
 
 
 
 
 
 
 
 
  
 test 
 Difference 
 p_value 
 Significance 
 
 
 
 
 c2 
 WT/WT vs Δ HEL/WT 
 0.0109 
 0.2000 
 FALSE 
 
 
 c3 
 Female vs Male 
 0.0373 
 0.0004 
 TRUE 
 
 
 c4 
 Female WT/WT vs Female Δ HEL/WT 
 0.0182 
 0.0953 
 FALSE 
 
 
 c5 
 Male WT/WT vs Male Δ HEL/WT 
 0.0036 
 0.9648 
 FALSE 
 
 
 c6 
 Female Δ HEL/WT vs Male Δ HEL/WT 
 0.0300 
 0.0630 
 FALSE 
 
 
 
     
 
 
  3.19.11  Testes weight
(sin.) 
 
 
 
 test 
 x 
 p_value 
 Significance 
 
 
 
 
 WT vs KO 
 -0.021708333 
 0.007173557 
 TRUE 
 
 
 
    
 
 
  3.19.12  Testes weight
(dx.) 
 
 
 
 test 
 x 
 p_value 
 Significance 
 
 
 
 
 WT vs KO 
 -0.021416667 
 0.008770578 
 TRUE 
 
 
 
    
 
 
 
  3.20  Immunology 
  Number of animals  
    
 
 
 
 STRAIN 
 GENDER 
 count 
 
 
 
 
 WT/WT 
 female 
 8 
 
 
 WT/WT 
 male 
 8 
 
 
 Δ HEL/WT 
 female 
 7 
 
 
 Δ HEL/WT 
 male 
 6 
 
 
 
   
        
  
 
  
 
 
  3.20.1  B cells - % of
live leukocytes (Panel B) 
 
 
 
 
 
 
 
 
 
 
  
 test 
 Difference 
 p_value 
 Significance 
 
 
 
 
 c2 
 WT/WT vs Δ HEL/WT 
 4.4207 
 0.0791 
 FALSE 
 
 
 c3 
 Female vs Male 
 -3.0688 
 0.7309 
 FALSE 
 
 
 c4 
 Female WT/WT vs Female Δ HEL/WT 
 3.5416 
 0.4749 
 FALSE 
 
 
 c5 
 Male WT/WT vs Male Δ HEL/WT 
 5.2998 
 0.1955 
 FALSE 
 
 
 c6 
 Female Δ HEL/WT vs Male Δ HEL/WT 
 -2.1897 
 0.9397 
 FALSE 
 
 
 
    
 
 
  3.20.2  B1a cells - % of B
cells (Panel B) 
 
 
 
 
 
 
 
 
 
 
  
 test 
 Difference 
 p_value 
 Significance 
 
 
 
 
 c2 
 WT/WT vs Δ HEL/WT 
 0.5632 
 0.3923 
 FALSE 
 
 
 c3 
 Female vs Male 
 0.0950 
 0.9937 
 FALSE 
 
 
 c4 
 Female WT/WT vs Female Δ HEL/WT 
 0.6935 
 0.4725 
 FALSE 
 
 
 c5 
 Male WT/WT vs Male Δ HEL/WT 
 0.4329 
 0.8400 
 FALSE 
 
 
 c6 
 Female Δ HEL/WT vs Male Δ HEL/WT 
 -0.0353 
 0.9999 
 FALSE 
 
 
 
    
 
 
  3.20.3  B1a cells - % of
live(Panel B) 
 
 
 
 
 
 
 
 
 
 
  
 test 
 Difference 
 p_value 
 Significance 
 
 
 
 
 c2 
 WT/WT vs Δ HEL/WT 
 0.3166 
 0.2847 
 FALSE 
 
 
 c3 
 Female vs Male 
 -0.0220 
 0.9993 
 FALSE 
 
 
 c4 
 Female WT/WT vs Female Δ HEL/WT 
 0.3271 
 0.5177 
 FALSE 
 
 
 c5 
 Male WT/WT vs Male Δ HEL/WT 
 0.3060 
 0.6432 
 FALSE 
 
 
 c6 
 Female Δ HEL/WT vs Male Δ HEL/WT 
 -0.0326 
 0.9993 
 FALSE 
 
 
 
    
 
 
  3.20.4  CD11b- NK cells -
% of live leukocytes (Panel B) 
 
 
 
 
 
 
 
 
 
 
  
 test 
 Difference 
 p_value 
 Significance 
 
 
 
 
 c2 
 WT/WT vs Δ HEL/WT 
 -0.4117 
 0.4862 
 FALSE 
 
 
 c3 
 Female vs Male 
 0.1674 
 0.9724 
 FALSE 
 
 
 c4 
 Female WT/WT vs Female Δ HEL/WT 
 -0.0975 
 0.9943 
 FALSE 
 
 
 c5 
 Male WT/WT vs Male Δ HEL/WT 
 -0.7259 
 0.3212 
 FALSE 
 
 
 c6 
 Female Δ HEL/WT vs Male Δ HEL/WT 
 -0.1468 
 0.9920 
 FALSE 
 
 
 
    
 
 
  3.20.5  CD11b- NK cells -
% of NK cells (Panel B) 
 
 
 
 
 
 
 
 
 
 
  
 test 
 Difference 
 p_value 
 Significance 
 
 
 
 
 c2 
 WT/WT vs Δ HEL/WT 
 1.2300 
 0.9994 
 FALSE 
 
 
 c3 
 Female vs Male 
 -13.9414 
 0.5355 
 FALSE 
 
 
 c4 
 Female WT/WT vs Female Δ HEL/WT 
 2.6429 
 0.9975 
 FALSE 
 
 
 c5 
 Male WT/WT vs Male Δ HEL/WT 
 -0.1829 
 1.0000 
 FALSE 
 
 
 c6 
 Female Δ HEL/WT vs Male Δ HEL/WT 
 -15.3543 
 0.7457 
 FALSE 
 
 
 
    
 
 
  3.20.6  CD11b-high cDC - %
of conventional DC (Panel B) 
 
 
 
 
 
 
 
 
 
 
  
 test 
 Difference 
 p_value 
 Significance 
 
 
 
 
 c2 
 WT/WT vs Δ HEL/WT 
 -0.1714 
 0.9998 
 FALSE 
 
 
 c3 
 Female vs Male 
 1.3000 
 0.9216 
 FALSE 
 
 
 c4 
 Female WT/WT vs Female Δ HEL/WT 
 -0.0143 
 1.0000 
 FALSE 
 
 
 c5 
 Male WT/WT vs Male Δ HEL/WT 
 -0.3286 
 0.9995 
 FALSE 
 
 
 c6 
 Female Δ HEL/WT vs Male Δ HEL/WT 
 1.1429 
 0.9817 
 FALSE 
 
 
 
    
 
 
  3.20.7  CD11b-high cDC - %
of live leukocytes (Panel B) 
 
 
 
 
 
 
 
 
 
 
  
 test 
 Difference 
 p_value 
 Significance 
 
 
 
 
 c2 
 WT/WT vs Δ HEL/WT 
 0.1680 
 0.0028 
 TRUE 
 
 
 c3 
 Female vs Male 
 -0.0249 
 0.9832 
 FALSE 
 
 
 c4 
 Female WT/WT vs Female Δ HEL/WT 
 0.2979 
 0.0000 
 TRUE 
 
 
 c5 
 Male WT/WT vs Male Δ HEL/WT 
 0.0381 
 0.9424 
 FALSE 
 
 
 c6 
 Female Δ HEL/WT vs Male Δ HEL/WT 
 -0.1548 
 0.3284 
 FALSE 
 
 
 
    
 
 
  3.20.8  CD11b-low cDC - %
of conventional DC (Panel B) 
 
 
 
 
 
 
 
 
 
 
  
 test 
 Difference 
 p_value 
 Significance 
 
 
 
 
 c2 
 WT/WT vs Δ HEL/WT 
 0.6428 
 0.9754 
 FALSE 
 
 
 c3 
 Female vs Male 
 -0.7371 
 0.9752 
 FALSE 
 
 
 c4 
 Female WT/WT vs Female Δ HEL/WT 
 2.0422 
 0.7613 
 FALSE 
 
 
 c5 
 Male WT/WT vs Male Δ HEL/WT 
 -0.7567 
 0.9870 
 FALSE 
 
 
 c6 
 Female Δ HEL/WT vs Male Δ HEL/WT 
 -2.1366 
 0.8259 
 FALSE 
 
 
 
    
 
 
  3.20.9  CD11b-low cDC - %
of of live leukocytes (Panel B) 
 
 
 
 
 
 
 
 
 
 
  
 test 
 Difference 
 p_value 
 Significance 
 
 
 
 
 c2 
 WT/WT vs Δ HEL/WT 
 0.0301 
 0.4404 
 FALSE 
 
 
 c3 
 Female vs Male 
 -0.0068 
 0.9935 
 FALSE 
 
 
 c4 
 Female WT/WT vs Female Δ HEL/WT 
 0.0642 
 0.0937 
 FALSE 
 
 
 c5 
 Male WT/WT vs Male Δ HEL/WT 
 -0.0039 
 0.9991 
 FALSE 
 
 
 c6 
 Female Δ HEL/WT vs Male Δ HEL/WT 
 -0.0409 
 0.6557 
 FALSE 
 
 
 
    
 
 
  3.20.10  CD11b+ NK cells -
% of live leukocytes (Panel B) 
 
 
 
 
 
 
 
 
 
 
  
 test 
 Difference 
 p_value 
 Significance 
 
 
 
 
 c2 
 WT/WT vs Δ HEL/WT 
 -0.7143 
 0.7375 
 FALSE 
 
 
 c3 
 Female vs Male 
 1.6164 
 0.3633 
 FALSE 
 
 
 c4 
 Female WT/WT vs Female Δ HEL/WT 
 -0.3065 
 0.9880 
 FALSE 
 
 
 c5 
 Male WT/WT vs Male Δ HEL/WT 
 -1.1222 
 0.6918 
 FALSE 
 
 
 c6 
 Female Δ HEL/WT vs Male Δ HEL/WT 
 1.2086 
 0.7914 
 FALSE 
 
 
 
    
 
 
  3.20.11  CD11b+ NK cells -
% of NK cells (Panel B) 
 
 
 
 
 
 
 
 
 
 
  
 test 
 Difference 
 p_value 
 Significance 
 
 
 
 
 c2 
 WT/WT vs Δ HEL/WT 
 -1.2357 
 0.9994 
 FALSE 
 
 
 c3 
 Female vs Male 
 13.9414 
 0.5356 
 FALSE 
 
 
 c4 
 Female WT/WT vs Female Δ HEL/WT 
 -2.6486 
 0.9975 
 FALSE 
 
 
 c5 
 Male WT/WT vs Male Δ HEL/WT 
 0.1771 
 1.0000 
 FALSE 
 
 
 c6 
 Female Δ HEL/WT vs Male Δ HEL/WT 
 15.3543 
 0.7457 
 FALSE 
 
 
 
    
 
 
  3.20.12  CD161+ B cells -
% of B cells (Panel B) 
 
 
 
 
 
 
 
 
 
 
  
 test 
 Difference 
 p_value 
 Significance 
 
 
 
 
 c2 
 WT/WT vs Δ HEL/WT 
 -0.0658 
 0.2064 
 FALSE 
 
 
 c3 
 Female vs Male 
 0.1236 
 0.1120 
 FALSE 
 
 
 c4 
 Female WT/WT vs Female Δ HEL/WT 
 -0.0411 
 0.7942 
 FALSE 
 
 
 c5 
 Male WT/WT vs Male Δ HEL/WT 
 -0.0904 
 0.2532 
 FALSE 
 
 
 c6 
 Female Δ HEL/WT vs Male Δ HEL/WT 
 0.0990 
 0.4905 
 FALSE 
 
 
 
    
 
 
  3.20.13  CD161+ B cells -
% of live leukocytes (Panel B) 
 
 
 
 
 
 
 
 
 
 
  
 test 
 Difference 
 p_value 
 Significance 
 
 
 
 
 c2 
 WT/WT vs Δ HEL/WT 
 -0.0293 
 0.2818 
 FALSE 
 
 
 c3 
 Female vs Male 
 0.0425 
 0.3456 
 FALSE 
 
 
 c4 
 Female WT/WT vs Female Δ HEL/WT 
 -0.0176 
 0.8523 
 FALSE 
 
 
 c5 
 Male WT/WT vs Male Δ HEL/WT 
 -0.0411 
 0.3171 
 FALSE 
 
 
 c6 
 Female Δ HEL/WT vs Male Δ HEL/WT 
 0.0307 
 0.7862 
 FALSE 
 
 
 
    
 
 
  3.20.14  CD4 T cells - %
of live leukocytes (Panel A) 
 
 
 
 
 
 
 
 
 
 
  
 test 
 Difference 
 p_value 
 Significance 
 
 
 
 
 c2 
 WT/WT vs Δ HEL/WT 
 -2.4586 
 0.3210 
 FALSE 
 
 
 c3 
 Female vs Male 
 -0.3507 
 0.9967 
 FALSE 
 
 
 c4 
 Female WT/WT vs Female Δ HEL/WT 
 -1.8941 
 0.7491 
 FALSE 
 
 
 c5 
 Male WT/WT vs Male Δ HEL/WT 
 -3.0231 
 0.4832 
 FALSE 
 
 
 c6 
 Female Δ HEL/WT vs Male Δ HEL/WT 
 -0.9152 
 0.9802 
 FALSE 
 
 
 
    
 
 
  3.20.15  CD4- NKT cells -
% of live leukocytes (Panel A) 
 
 
 
 
 
 
 
 
 
 
  
 test 
 Difference 
 p_value 
 Significance 
 
 
 
 
 c2 
 WT/WT vs Δ HEL/WT 
 -0.0104 
 0.9986 
 FALSE 
 
 
 c3 
 Female vs Male 
 0.1502 
 0.1532 
 FALSE 
 
 
 c4 
 Female WT/WT vs Female Δ HEL/WT 
 -0.0317 
 0.9834 
 FALSE 
 
 
 c5 
 Male WT/WT vs Male Δ HEL/WT 
 0.0110 
 0.9995 
 FALSE 
 
 
 c6 
 Female Δ HEL/WT vs Male Δ HEL/WT 
 0.1715 
 0.3500 
 FALSE 
 
 
 
    
 
 
  3.20.16  CD4- NKT cells -
% of NKT cells (Panel A) 
 
 
 
 
 
 
 
 
 
 
  
 test 
 Difference 
 p_value 
 Significance 
 
 
 
 
 c2 
 WT/WT vs Δ HEL/WT 
 -11.3318 
 0.2448 
 FALSE 
 
 
 c3 
 Female vs Male 
 8.6945 
 0.4951 
 FALSE 
 
 
 c4 
 Female WT/WT vs Female Δ HEL/WT 
 -15.9909 
 0.1963 
 FALSE 
 
 
 c5 
 Male WT/WT vs Male Δ HEL/WT 
 -6.6728 
 0.8781 
 FALSE 
 
 
 c6 
 Female Δ HEL/WT vs Male Δ HEL/WT 
 13.3536 
 0.4658 
 FALSE 
 
 
 
    
 
 
  3.20.17  CD4+ NKT cells -
% of live leukocytes (Panel A) 
 
 
 
 
 
 
 
 
 
 
  
 test 
 Difference 
 p_value 
 Significance 
 
 
 
 
 c2 
 WT/WT vs Δ HEL/WT 
 0.1391 
 0.1181 
 FALSE 
 
 
 c3 
 Female vs Male 
 0.1009 
 0.3641 
 FALSE 
 
 
 c4 
 Female WT/WT vs Female Δ HEL/WT 
 0.2114 
 0.0552 
 FALSE 
 
 
 c5 
 Male WT/WT vs Male Δ HEL/WT 
 0.0669 
 0.8856 
 FALSE 
 
 
 c6 
 Female Δ HEL/WT vs Male Δ HEL/WT 
 0.0286 
 0.9894 
 FALSE 
 
 
 
    
 
 
  3.20.18  CD4+ NKT cells -
% of NKT cells (Panel A) 
 
 
 
 
 
 
 
 
 
 
  
 test 
 Difference 
 p_value 
 Significance 
 
 
 
 
 c2 
 WT/WT vs Δ HEL/WT 
 11.3224 
 0.2507 
 FALSE 
 
 
 c3 
 Female vs Male 
 -8.5824 
 0.5085 
 FALSE 
 
 
 c4 
 Female WT/WT vs Female Δ HEL/WT 
 15.8749 
 0.2064 
 FALSE 
 
 
 c5 
 Male WT/WT vs Male Δ HEL/WT 
 6.7700 
 0.8757 
 FALSE 
 
 
 c6 
 Female Δ HEL/WT vs Male Δ HEL/WT 
 -13.1348 
 0.4836 
 FALSE 
 
 
 
    
 
 
  3.20.19  CD4+ T cells - %
of T cells (Panel A) 
 
 
 
 
 
 
 
 
 
 
  
 test 
 Difference 
 p_value 
 Significance 
 
 
 
 
 c2 
 WT/WT vs Δ HEL/WT 
 -0.8317 
 0.9943 
 FALSE 
 
 
 c3 
 Female vs Male 
 2.6819 
 0.9087 
 FALSE 
 
 
 c4 
 Female WT/WT vs Female Δ HEL/WT 
 0.2302 
 0.9999 
 FALSE 
 
 
 c5 
 Male WT/WT vs Male Δ HEL/WT 
 -1.8936 
 0.9800 
 FALSE 
 
 
 c6 
 Female Δ HEL/WT vs Male Δ HEL/WT 
 1.6200 
 0.9916 
 FALSE 
 
 
 
    
 
 
  3.20.20  CD4+ T helper
cells - % of CD4 T cells (Panel A) 
 
 
 
 
 
 
 
 
 
 
  
 test 
 Difference 
 p_value 
 Significance 
 
 
 
 
 c2 
 WT/WT vs Δ HEL/WT 
 -0.9725 
 0.1080 
 FALSE 
 
 
 c3 
 Female vs Male 
 0.5275 
 0.5968 
 FALSE 
 
 
 c4 
 Female WT/WT vs Female Δ HEL/WT 
 0.2464 
 0.9708 
 FALSE 
 
 
 c5 
 Male WT/WT vs Male Δ HEL/WT 
 -2.1914 
 0.0046 
 TRUE 
 
 
 c6 
 Female Δ HEL/WT vs Male Δ HEL/WT 
 -0.6914 
 0.6935 
 FALSE 
 
 
 
    
 
 
  3.20.21  CD4+ T helper
cells - %oflive leukocytes (Panel A 
 
 
 
 
 
 
 
 
 
 
  
 test 
 Difference 
 p_value 
 Significance 
 
 
 
 
 c2 
 WT/WT vs Δ HEL/WT 
 -2.4832 
 0.2692 
 FALSE 
 
 
 c3 
 Female vs Male 
 -0.2931 
 0.9977 
 FALSE 
 
 
 c4 
 Female WT/WT vs Female Δ HEL/WT 
 -1.6931 
 0.7819 
 FALSE 
 
 
 c5 
 Male WT/WT vs Male Δ HEL/WT 
 -3.2734 
 0.3688 
 FALSE 
 
 
 c6 
 Female Δ HEL/WT vs Male Δ HEL/WT 
 -1.0832 
 0.9622 
 FALSE 
 
 
 
    
 
 
  3.20.22  CD8+ T cells - %
of live leukocytes (Panel A) 
 
 
 
 
 
 
 
 
 
 
  
 test 
 Difference 
 p_value 
 Significance 
 
 
 
 
 c2 
 WT/WT vs Δ HEL/WT 
 -0.7481 
 0.8629 
 FALSE 
 
 
 c3 
 Female vs Male 
 -0.5224 
 0.9474 
 FALSE 
 
 
 c4 
 Female WT/WT vs Female Δ HEL/WT 
 -1.2111 
 0.7760 
 FALSE 
 
 
 c5 
 Male WT/WT vs Male Δ HEL/WT 
 -0.2851 
 0.9972 
 FALSE 
 
 
 c6 
 Female Δ HEL/WT vs Male Δ HEL/WT 
 -0.0594 
 1.0000 
 FALSE 
 
 
 
    
 
 
  3.20.23  CD8+ T cells - %
of T cells (Panel A) 
 
 
 
 
 
 
 
 
 
 
  
 test 
 Difference 
 p_value 
 Significance 
 
 
 
 
 c2 
 WT/WT vs Δ HEL/WT 
 0.6451 
 0.9972 
 FALSE 
 
 
 c3 
 Female vs Male 
 -2.6172 
 0.9083 
 FALSE 
 
 
 c4 
 Female WT/WT vs Female Δ HEL/WT 
 -0.5025 
 0.9994 
 FALSE 
 
 
 c5 
 Male WT/WT vs Male Δ HEL/WT 
 1.7927 
 0.9822 
 FALSE 
 
 
 c6 
 Female Δ HEL/WT vs Male Δ HEL/WT 
 -1.4697 
 0.9933 
 FALSE 
 
 
 
    
 
 
  3.20.24  Conventional DC -
% of live leukocytes (Panel B) 
 
 
 
 
 
 
 
 
 
 
  
 test 
 Difference 
 p_value 
 Significance 
 
 
 
 
 c2 
 WT/WT vs Δ HEL/WT 
 0.2170 
 0.0014 
 TRUE 
 
 
 c3 
 Female vs Male 
 -0.0300 
 0.9810 
 FALSE 
 
 
 c4 
 Female WT/WT vs Female Δ HEL/WT 
 0.3796 
 0.0000 
 TRUE 
 
 
 c5 
 Male WT/WT vs Male Δ HEL/WT 
 0.0544 
 0.9096 
 FALSE 
 
 
 c6 
 Female Δ HEL/WT vs Male Δ HEL/WT 
 -0.1926 
 0.2739 
 FALSE 
 
 
 
    
 
 
  3.20.25  Effector CD4- NKT
c. - % of CD4- NKT c. (Panel A) 
 
 
 
 
 
 
 
 
 
 
  
 test 
 Difference 
 p_value 
 Significance 
 
 
 
 
 c2 
 WT/WT vs Δ HEL/WT 
 9.0932 
 0.2433 
 FALSE 
 
 
 c3 
 Female vs Male 
 -4.9282 
 0.7347 
 FALSE 
 
 
 c4 
 Female WT/WT vs Female Δ HEL/WT 
 12.3893 
 0.2212 
 FALSE 
 
 
 c5 
 Male WT/WT vs Male Δ HEL/WT 
 5.7971 
 0.8512 
 FALSE 
 
 
 c6 
 Female Δ HEL/WT vs Male Δ HEL/WT 
 -8.2243 
 0.6648 
 FALSE 
 
 
 
    
 
 
  3.20.26  Effector CD4- NKT
cells - % of live (Panel A) 
 
 
 
 
 
 
 
 
 
 
  
 test 
 Difference 
 p_value 
 Significance 
 
 
 
 
 c2 
 WT/WT vs Δ HEL/WT 
 0.0307 
 0.5536 
 FALSE 
 
 
 c3 
 Female vs Male 
 0.0605 
 0.0841 
 FALSE 
 
 
 c4 
 Female WT/WT vs Female Δ HEL/WT 
 0.0385 
 0.5991 
 FALSE 
 
 
 c5 
 Male WT/WT vs Male Δ HEL/WT 
 0.0229 
 0.9101 
 FALSE 
 
 
 c6 
 Female Δ HEL/WT vs Male Δ HEL/WT 
 0.0527 
 0.4762 
 FALSE 
 
 
 
    
 
 
  3.20.27  Effector CD4+ NKT
c. - % of CD4+ NKT c. (Panel A) 
 
 
 
 
 
 
 
 
 
 
  
 test 
 Difference 
 p_value 
 Significance 
 
 
 
 
 c2 
 WT/WT vs Δ HEL/WT 
 13.7384 
 0.3673 
 FALSE 
 
 
 c3 
 Female vs Male 
 -3.6259 
 0.9725 
 FALSE 
 
 
 c4 
 Female WT/WT vs Female Δ HEL/WT 
 13.2339 
 0.6351 
 FALSE 
 
 
 c5 
 Male WT/WT vs Male Δ HEL/WT 
 14.2429 
 0.6709 
 FALSE 
 
 
 c6 
 Female Δ HEL/WT vs Male Δ HEL/WT 
 -3.1214 
 0.9945 
 FALSE 
 
 
 
    
 
 
  3.20.28  Effector CD4+ NKT
cells - % of live (Panel A) 
 
 
 
 
 
 
 
 
 
 
  
 test 
 Difference 
 p_value 
 Significance 
 
 
 
 
 c2 
 WT/WT vs Δ HEL/WT 
 0.1498 
 0.0413 
 TRUE 
 
 
 c3 
 Female vs Male 
 0.0789 
 0.4865 
 FALSE 
 
 
 c4 
 Female WT/WT vs Female Δ HEL/WT 
 0.2056 
 0.0322 
 TRUE 
 
 
 c5 
 Male WT/WT vs Male Δ HEL/WT 
 0.0940 
 0.6680 
 FALSE 
 
 
 c6 
 Female Δ HEL/WT vs Male Δ HEL/WT 
 0.0230 
 0.9923 
 FALSE 
 
 
 
    
 
 
  3.20.29  Effector CD4+ T
help c.-% of CD4+ T help (Panel A) 
 
 
 
 
 
 
 
 
 
 
  
 test 
 Difference 
 p_value 
 Significance 
 
 
 
 
 c2 
 WT/WT vs Δ HEL/WT 
 6.4015 
 0.0008 
 TRUE 
 
 
 c3 
 Female vs Male 
 10.3422 
 0.0001 
 TRUE 
 
 
 c4 
 Female WT/WT vs Female Δ HEL/WT 
 4.4402 
 0.1801 
 FALSE 
 
 
 c5 
 Male WT/WT vs Male Δ HEL/WT 
 8.3628 
 0.0037 
 TRUE 
 
 
 c6 
 Female Δ HEL/WT vs Male Δ HEL/WT 
 12.3036 
 0.0008 
 TRUE 
 
 
 
    
 
 
  3.20.30  Effector CD4+ T
helper cells - % of live (Panel A) 
 
 
 
 
 
 
 
 
 
 
  
 test 
 Difference 
 p_value 
 Significance 
 
 
 
 
 c2 
 WT/WT vs Δ HEL/WT 
 0.4829 
 0.3511 
 FALSE 
 
 
 c3 
 Female vs Male 
 2.5807 
 0.0000 
 TRUE 
 
 
 c4 
 Female WT/WT vs Female Δ HEL/WT 
 0.0867 
 0.9960 
 FALSE 
 
 
 c5 
 Male WT/WT vs Male Δ HEL/WT 
 0.8791 
 0.1778 
 FALSE 
 
 
 c6 
 Female Δ HEL/WT vs Male Δ HEL/WT 
 2.9769 
 0.0001 
 TRUE 
 
 
 
    
 
 
  3.20.31  Effector CD8+ T
cells - % of CD8+ T cells (Panel A 
 
 
 
 
 
 
 
 
 
 
  
 test 
 Difference 
 p_value 
 Significance 
 
 
 
 
 c2 
 WT/WT vs Δ HEL/WT 
 1.1576 
 0.7706 
 FALSE 
 
 
 c3 
 Female vs Male 
 8.3663 
 0.0004 
 TRUE 
 
 
 c4 
 Female WT/WT vs Female Δ HEL/WT 
 1.4299 
 0.8161 
 FALSE 
 
 
 c5 
 Male WT/WT vs Male Δ HEL/WT 
 0.8854 
 0.9576 
 FALSE 
 
 
 c6 
 Female Δ HEL/WT vs Male Δ HEL/WT 
 8.0940 
 0.0180 
 TRUE 
 
 
 
    
 
 
  3.20.32  Effector CD8+ T
cells - %oflive leukocytes (Pane A 
 
 
 
 
 
 
 
 
 
 
  
 test 
 Difference 
 p_value 
 Significance 
 
 
 
 
 c2 
 WT/WT vs Δ HEL/WT 
 0.0556 
 0.7539 
 FALSE 
 
 
 c3 
 Female vs Male 
 0.2329 
 0.0715 
 FALSE 
 
 
 c4 
 Female WT/WT vs Female Δ HEL/WT 
 0.0899 
 0.6399 
 FALSE 
 
 
 c5 
 Male WT/WT vs Male Δ HEL/WT 
 0.0213 
 0.9937 
 FALSE 
 
 
 c6 
 Female Δ HEL/WT vs Male Δ HEL/WT 
 0.1986 
 0.3736 
 FALSE 
 
 
 
    
 
 
  3.20.33  Effector NK cells
- % of live leukocytes (Panel A) 
 
 
 
 
 
 
 
 
 
 
  
 test 
 Difference 
 p_value 
 Significance 
 
 
 
 
 c2 
 WT/WT vs Δ HEL/WT 
 0.0816 
 0.9017 
 FALSE 
 
 
 c3 
 Female vs Male 
 0.5073 
 0.0452 
 TRUE 
 
 
 c4 
 Female WT/WT vs Female Δ HEL/WT 
 0.1738 
 0.7032 
 FALSE 
 
 
 c5 
 Male WT/WT vs Male Δ HEL/WT 
 -0.0107 
 0.9999 
 FALSE 
 
 
 c6 
 Female Δ HEL/WT vs Male Δ HEL/WT 
 0.4151 
 0.3526 
 FALSE 
 
 
 
    
 
 
  3.20.34  Effector NK cells
- % of NK cells (Panel A) 
 
 
 
 
 
 
 
 
 
 
  
 test 
 Difference 
 p_value 
 Significance 
 
 
 
 
 c2 
 WT/WT vs Δ HEL/WT 
 5.4861 
 0.0000 
 TRUE 
 
 
 c3 
 Female vs Male 
 1.1614 
 0.9061 
 FALSE 
 
 
 c4 
 Female WT/WT vs Female Δ HEL/WT 
 4.8049 
 0.0132 
 TRUE 
 
 
 c5 
 Male WT/WT vs Male Δ HEL/WT 
 6.1673 
 0.0022 
 TRUE 
 
 
 c6 
 Female Δ HEL/WT vs Male Δ HEL/WT 
 1.8426 
 0.8525 
 FALSE 
 
 
 
    
 
 
  3.20.35  Effector Treg
cells - % of live leukocytes (Panel A) 
 
 
 
 
 
 
 
 
 
 
  
 test 
 Difference 
 p_value 
 Significance 
 
 
 
 
 c2 
 WT/WT vs Δ HEL/WT 
 0.0744 
 0.2147 
 FALSE 
 
 
 c3 
 Female vs Male 
 0.1026 
 0.3018 
 FALSE 
 
 
 c4 
 Female WT/WT vs Female Δ HEL/WT 
 -0.0062 
 0.9993 
 FALSE 
 
 
 c5 
 Male WT/WT vs Male Δ HEL/WT 
 0.1549 
 0.0353 
 TRUE 
 
 
 c6 
 Female Δ HEL/WT vs Male Δ HEL/WT 
 0.1831 
 0.0968 
 FALSE 
 
 
 
    
 
 
  3.20.36  Effector Treg
cells - % of Treg cells (Panel A) 
 
 
 
 
 
 
 
 
 
 
  
 test 
 Difference 
 p_value 
 Significance 
 
 
 
 
 c2 
 WT/WT vs Δ HEL/WT 
 5.4316 
 0.0097 
 TRUE 
 
 
 c3 
 Female vs Male 
 7.0321 
 0.0327 
 TRUE 
 
 
 c4 
 Female WT/WT vs Female Δ HEL/WT 
 5.0730 
 0.1227 
 FALSE 
 
 
 c5 
 Male WT/WT vs Male Δ HEL/WT 
 5.7903 
 0.0983 
 FALSE 
 
 
 c6 
 Female Δ HEL/WT vs Male Δ HEL/WT 
 7.3907 
 0.1325 
 FALSE 
 
 
 
    
 
 
  3.20.37  Eosinophils - %
of live leukocytes (Panel B) 
 
 
 
 
 
 
 
 
 
 
  
 test 
 Difference 
 p_value 
 Significance 
 
 
 
 
 c2 
 WT/WT vs Δ HEL/WT 
 0.0504 
 0.9241 
 FALSE 
 
 
 c3 
 Female vs Male 
 0.2242 
 0.1683 
 FALSE 
 
 
 c4 
 Female WT/WT vs Female Δ HEL/WT 
 0.0643 
 0.9347 
 FALSE 
 
 
 c5 
 Male WT/WT vs Male Δ HEL/WT 
 0.0364 
 0.9897 
 FALSE 
 
 
 c6 
 Female Δ HEL/WT vs Male Δ HEL/WT 
 0.2102 
 0.4726 
 FALSE 
 
 
 
    
 
 
  3.20.38  Follicular B
cells - % of B cells (Panel B) 
 
 
 
 
 
 
 
 
 
 
  
 test 
 Difference 
 p_value 
 Significance 
 
 
 
 
 c2 
 WT/WT vs Δ HEL/WT 
 -0.3857 
 0.9713 
 FALSE 
 
 
 c3 
 Female vs Male 
 -1.2571 
 0.4862 
 FALSE 
 
 
 c4 
 Female WT/WT vs Female Δ HEL/WT 
 -1.7571 
 0.4548 
 FALSE 
 
 
 c5 
 Male WT/WT vs Male Δ HEL/WT 
 0.9857 
 0.8718 
 FALSE 
 
 
 c6 
 Female Δ HEL/WT vs Male Δ HEL/WT 
 0.1143 
 0.9998 
 FALSE 
 
 
 
    
 
 
  3.20.39  Follicular B
cells - % of B cells LIVE (Panel B) 
 
 
 
 
 
 
 
 
 
 
  
 test 
 Difference 
 p_value 
 Significance 
 
 
 
 
 c2 
 WT/WT vs Δ HEL/WT 
 3.1091 
 0.1866 
 FALSE 
 
 
 c3 
 Female vs Male 
 -3.5527 
 0.4842 
 FALSE 
 
 
 c4 
 Female WT/WT vs Female Δ HEL/WT 
 1.8494 
 0.8054 
 FALSE 
 
 
 c5 
 Male WT/WT vs Male Δ HEL/WT 
 4.3688 
 0.2146 
 FALSE 
 
 
 c6 
 Female Δ HEL/WT vs Male Δ HEL/WT 
 -2.2929 
 0.8891 
 FALSE 
 
 
 
    
 
 
  3.20.40  Granulocytes - %
of live leukocytes (Panel B) 
 
 
 
 
 
 
 
 
 
 
  
 test 
 Difference 
 p_value 
 Significance 
 
 
 
 
 c2 
 WT/WT vs Δ HEL/WT 
 0.2886 
 0.6983 
 FALSE 
 
 
 c3 
 Female vs Male 
 0.8961 
 0.1651 
 FALSE 
 
 
 c4 
 Female WT/WT vs Female Δ HEL/WT 
 0.3716 
 0.7313 
 FALSE 
 
 
 c5 
 Male WT/WT vs Male Δ HEL/WT 
 0.2055 
 0.9507 
 FALSE 
 
 
 c6 
 Female Δ HEL/WT vs Male Δ HEL/WT 
 0.8130 
 0.4605 
 FALSE 
 
 
 
    
 
 
  3.20.41  Live leukocytes
(Panel A) - % of total events 
 
 
 
 
 
 
 
 
 
 
  
 test 
 Difference 
 p_value 
 Significance 
 
 
 
 
 c2 
 WT/WT vs Δ HEL/WT 
 4.0820 
 0.1556 
 FALSE 
 
 
 c3 
 Female vs Male 
 -4.2895 
 0.1243 
 FALSE 
 
 
 c4 
 Female WT/WT vs Female Δ HEL/WT 
 1.2125 
 0.9633 
 FALSE 
 
 
 c5 
 Male WT/WT vs Male Δ HEL/WT 
 6.9514 
 0.0837 
 FALSE 
 
 
 c6 
 Female Δ HEL/WT vs Male Δ HEL/WT 
 -1.4200 
 0.9596 
 FALSE 
 
 
 
    
 
 
  3.20.42  Live leukocytes
(Panel B) - % of total events 
 
 
 
 
 
 
 
 
 
 
  
 test 
 Difference 
 p_value 
 Significance 
 
 
 
 
 c2 
 WT/WT vs Δ HEL/WT 
 4.6603 
 0.2605 
 FALSE 
 
 
 c3 
 Female vs Male 
 -8.6319 
 0.0791 
 FALSE 
 
 
 c4 
 Female WT/WT vs Female Δ HEL/WT 
 1.5898 
 0.9652 
 FALSE 
 
 
 c5 
 Male WT/WT vs Male Δ HEL/WT 
 7.7308 
 0.1647 
 FALSE 
 
 
 c6 
 Female Δ HEL/WT vs Male Δ HEL/WT 
 -5.5614 
 0.6375 
 FALSE 
 
 
 
    
 
 
  3.20.43  Ly6C+ CD11b- NK
cells - % of live (Panel B) 
 
 
 
 
 
 
 
 
 
 
  
 test 
 Difference 
 p_value 
 Significance 
 
 
 
 
 c2 
 WT/WT vs Δ HEL/WT 
 -0.0755 
 0.5610 
 FALSE 
 
 
 c3 
 Female vs Male 
 -0.0216 
 0.9876 
 FALSE 
 
 
 c4 
 Female WT/WT vs Female Δ HEL/WT 
 0.0188 
 0.9949 
 FALSE 
 
 
 c5 
 Male WT/WT vs Male Δ HEL/WT 
 -0.1698 
 0.1985 
 FALSE 
 
 
 c6 
 Female Δ HEL/WT vs Male Δ HEL/WT 
 -0.1159 
 0.5972 
 FALSE 
 
 
 
    
 
 
  3.20.44  Ly6C+ CD11b- NK
cells - % of NK cells (Panel B) 
 
 
 
 
 
 
 
 
 
 
  
 test 
 Difference 
 p_value 
 Significance 
 
 
 
 
 c2 
 WT/WT vs Δ HEL/WT 
 -0.0970 
 0.9995 
 FALSE 
 
 
 c3 
 Female vs Male 
 -1.9856 
 0.1038 
 FALSE 
 
 
 c4 
 Female WT/WT vs Female Δ HEL/WT 
 0.6257 
 0.9477 
 FALSE 
 
 
 c5 
 Male WT/WT vs Male Δ HEL/WT 
 -0.8197 
 0.9149 
 FALSE 
 
 
 c6 
 Female Δ HEL/WT vs Male Δ HEL/WT 
 -2.7083 
 0.1506 
 FALSE 
 
 
 
    
 
 
  3.20.45  Ly6C+ CD11b+ NK
cells - % of live (Panel B) 
 
 
 
 
 
 
 
 
 
 
  
 test 
 Difference 
 p_value 
 Significance 
 
 
 
 
 c2 
 WT/WT vs Δ HEL/WT 
 -0.1469 
 0.9500 
 FALSE 
 
 
 c3 
 Female vs Male 
 0.6043 
 0.4077 
 FALSE 
 
 
 c4 
 Female WT/WT vs Female Δ HEL/WT 
 0.0318 
 0.9998 
 FALSE 
 
 
 c5 
 Male WT/WT vs Male Δ HEL/WT 
 -0.3257 
 0.8487 
 FALSE 
 
 
 c6 
 Female Δ HEL/WT vs Male Δ HEL/WT 
 0.4256 
 0.8404 
 FALSE 
 
 
 
    
 
 
  3.20.46  Ly6C+ CD11b+ NK
cells - % of NK cells (Panel B) 
 
 
 
 
 
 
 
 
 
 
  
 test 
 Difference 
 p_value 
 Significance 
 
 
 
 
 c2 
 WT/WT vs Δ HEL/WT 
 2.1306 
 0.9675 
 FALSE 
 
 
 c3 
 Female vs Male 
 4.8706 
 0.7215 
 FALSE 
 
 
 c4 
 Female WT/WT vs Female Δ HEL/WT 
 1.9971 
 0.9886 
 FALSE 
 
 
 c5 
 Male WT/WT vs Male Δ HEL/WT 
 2.2640 
 0.9874 
 FALSE 
 
 
 c6 
 Female Δ HEL/WT vs Male Δ HEL/WT 
 5.0040 
 0.8845 
 FALSE 
 
 
 
    
 
 
  3.20.47  Ly6C+ NKT cells -
% of live leukocytes (Panel B) 
 
 
 
 
 
 
 
 
 
 
  
 test 
 Difference 
 p_value 
 Significance 
 
 
 
 
 c2 
 WT/WT vs Δ HEL/WT 
 0.0628 
 0.8536 
 FALSE 
 
 
 c3 
 Female vs Male 
 0.1749 
 0.1284 
 FALSE 
 
 
 c4 
 Female WT/WT vs Female Δ HEL/WT 
 0.1389 
 0.5598 
 FALSE 
 
 
 c5 
 Male WT/WT vs Male Δ HEL/WT 
 -0.0132 
 0.9995 
 FALSE 
 
 
 c6 
 Female Δ HEL/WT vs Male Δ HEL/WT 
 0.0988 
 0.8289 
 FALSE 
 
 
 
    
 
 
  3.20.48  Ly6C+ NKT cells -
% of NKT cells (Panel B) 
 
 
 
 
 
 
 
 
 
 
  
 test 
 Difference 
 p_value 
 Significance 
 
 
 
 
 c2 
 WT/WT vs Δ HEL/WT 
 -6.9440 
 0.7296 
 FALSE 
 
 
 c3 
 Female vs Male 
 0.7360 
 0.9995 
 FALSE 
 
 
 c4 
 Female WT/WT vs Female Δ HEL/WT 
 0.9343 
 0.9996 
 FALSE 
 
 
 c5 
 Male WT/WT vs Male Δ HEL/WT 
 -14.8223 
 0.4472 
 FALSE 
 
 
 c6 
 Female Δ HEL/WT vs Male Δ HEL/WT 
 -7.1423 
 0.8881 
 FALSE 
 
 
 
    
 
 
  3.20.49  Marginal zone B
cells - % B cells LIVE (Panel B) 
 
 
 
 
 
 
 
 
 
 
  
 test 
 Difference 
 p_value 
 Significance 
 
 
 
 
 c2 
 WT/WT vs Δ HEL/WT 
 0.1682 
 0.9570 
 FALSE 
 
 
 c3 
 Female vs Male 
 0.7197 
 0.5992 
 FALSE 
 
 
 c4 
 Female WT/WT vs Female Δ HEL/WT 
 0.4694 
 0.7300 
 FALSE 
 
 
 c5 
 Male WT/WT vs Male Δ HEL/WT 
 -0.1330 
 0.9927 
 FALSE 
 
 
 c6 
 Female Δ HEL/WT vs Male Δ HEL/WT 
 0.4185 
 0.9407 
 FALSE 
 
 
 
    
 
 
  3.20.50  Marginal zone B
cells - % of B cells (Panel B) 
 
 
 
 
 
 
 
 
 
 
  
 test 
 Difference 
 p_value 
 Significance 
 
 
 
 
 c2 
 WT/WT vs Δ HEL/WT 
 -0.4758 
 0.8752 
 FALSE 
 
 
 c3 
 Female vs Male 
 2.1288 
 0.2108 
 FALSE 
 
 
 c4 
 Female WT/WT vs Female Δ HEL/WT 
 0.2088 
 0.9949 
 FALSE 
 
 
 c5 
 Male WT/WT vs Male Δ HEL/WT 
 -1.1604 
 0.5974 
 FALSE 
 
 
 c6 
 Female Δ HEL/WT vs Male Δ HEL/WT 
 1.4442 
 0.7225 
 FALSE 
 
 
 
    
 
 
  3.20.51  Monocytes - % of
live leukocytes (Panel B) 
 
 
 
 
 
 
 
 
 
 
  
 test 
 Difference 
 p_value 
 Significance 
 
 
 
 
 c2 
 WT/WT vs Δ HEL/WT 
 0.0883 
 0.8989 
 FALSE 
 
 
 c3 
 Female vs Male 
 -0.2189 
 0.3240 
 FALSE 
 
 
 c4 
 Female WT/WT vs Female Δ HEL/WT 
 0.0900 
 0.9527 
 FALSE 
 
 
 c5 
 Male WT/WT vs Male Δ HEL/WT 
 0.0866 
 0.9672 
 FALSE 
 
 
 c6 
 Female Δ HEL/WT vs Male Δ HEL/WT 
 -0.2206 
 0.6461 
 FALSE 
 
 
 
    
 
 
  3.20.52  Naive CD8+ T
cells - % of CD8+ T cells (Panel A) 
 
 
 
 
 
 
 
 
 
 
  
 test 
 Difference 
 p_value 
 Significance 
 
 
 
 
 c2 
 WT/WT vs Δ HEL/WT 
 -5.3214 
 0.3534 
 FALSE 
 
 
 c3 
 Female vs Male 
 -17.4441 
 0.0072 
 TRUE 
 
 
 c4 
 Female WT/WT vs Female Δ HEL/WT 
 -6.1069 
 0.4970 
 FALSE 
 
 
 c5 
 Male WT/WT vs Male Δ HEL/WT 
 -4.5358 
 0.7653 
 FALSE 
 
 
 c6 
 Female Δ HEL/WT vs Male Δ HEL/WT 
 -16.6585 
 0.0842 
 FALSE 
 
 
 
    
 
 
  3.20.53  Naive CD8+ T
cells - % of live leukocytes (Panel A 
 
 
 
 
 
 
 
 
 
 
  
 test 
 Difference 
 p_value 
 Significance 
 
 
 
 
 c2 
 WT/WT vs Δ HEL/WT 
 -0.9382 
 0.6791 
 FALSE 
 
 
 c3 
 Female vs Male 
 -1.9330 
 0.3458 
 FALSE 
 
 
 c4 
 Female WT/WT vs Female Δ HEL/WT 
 -1.2120 
 0.7027 
 FALSE 
 
 
 c5 
 Male WT/WT vs Male Δ HEL/WT 
 -0.6643 
 0.9484 
 FALSE 
 
 
 c6 
 Female Δ HEL/WT vs Male Δ HEL/WT 
 -1.6591 
 0.7146 
 FALSE 
 
 
 
    
 
 
  3.20.54  NK cells (Panel
A) - % of live l(Panel A) 
 
 
 
 
 
 
 
 
 
 
  
 test 
 Difference 
 p_value 
 Significance 
 
 
 
 
 c2 
 WT/WT vs Δ HEL/WT 
 -1.3504 
 0.3775 
 FALSE 
 
 
 c3 
 Female vs Male 
 2.0114 
 0.3451 
 FALSE 
 
 
 c4 
 Female WT/WT vs Female Δ HEL/WT 
 -0.8611 
 0.8660 
 FALSE 
 
 
 c5 
 Male WT/WT vs Male Δ HEL/WT 
 -1.8397 
 0.4431 
 FALSE 
 
 
 c6 
 Female Δ HEL/WT vs Male Δ HEL/WT 
 1.5221 
 0.7797 
 FALSE 
 
 
 
    
 
 
  3.20.55  NK cells (Panel
B) - % of live leukocytes (Panel B 
 
 
 
 
 
 
 
 
 
 
  
 test 
 Difference 
 p_value 
 Significance 
 
 
 
 
 c2 
 WT/WT vs Δ HEL/WT 
 -1.1345 
 0.6203 
 FALSE 
 
 
 c3 
 Female vs Male 
 1.8564 
 0.4980 
 FALSE 
 
 
 c4 
 Female WT/WT vs Female Δ HEL/WT 
 -0.4260 
 0.9864 
 FALSE 
 
 
 c5 
 Male WT/WT vs Male Δ HEL/WT 
 -1.8430 
 0.5354 
 FALSE 
 
 
 c6 
 Female Δ HEL/WT vs Male Δ HEL/WT 
 1.1479 
 0.9123 
 FALSE 
 
 
 
    
 
 
  3.20.56  NKT cells (panel
A) - % of live (Panel A) 
 
 
 
 
 
 
 
 
 
 
  
 test 
 Difference 
 p_value 
 Significance 
 
 
 
 
 c2 
 WT/WT vs Δ HEL/WT 
 0.1348 
 0.6815 
 FALSE 
 
 
 c3 
 Female vs Male 
 0.2457 
 0.1890 
 FALSE 
 
 
 c4 
 Female WT/WT vs Female Δ HEL/WT 
 0.1892 
 0.6407 
 FALSE 
 
 
 c5 
 Male WT/WT vs Male Δ HEL/WT 
 0.0804 
 0.9703 
 FALSE 
 
 
 c6 
 Female Δ HEL/WT vs Male Δ HEL/WT 
 0.1913 
 0.7170 
 FALSE 
 
 
 
    
 
 
  3.20.57  NKT cells (panel
B) - % of live leukocytes (Panel 
 
 
 
 
 
 
 
 
 
 
  
 test 
 Difference 
 p_value 
 Significance 
 
 
 
 
 c2 
 WT/WT vs Δ HEL/WT 
 0.2175 
 0.4931 
 FALSE 
 
 
 c3 
 Female vs Male 
 0.3993 
 0.0546 
 FALSE 
 
 
 c4 
 Female WT/WT vs Female Δ HEL/WT 
 0.3036 
 0.4628 
 FALSE 
 
 
 c5 
 Male WT/WT vs Male Δ HEL/WT 
 0.1313 
 0.9373 
 FALSE 
 
 
 c6 
 Female Δ HEL/WT vs Male Δ HEL/WT 
 0.3131 
 0.5151 
 FALSE 
 
 
 
    
 
 
  3.20.58  Resting CD4- NKT
c. - % of CD4- NKT c. (Panel A) 
 
 
 
 
 
 
 
 
 
 
  
 test 
 Difference 
 p_value 
 Significance 
 
 
 
 
 c2 
 WT/WT vs Δ HEL/WT 
 -7.2358 
 0.6605 
 FALSE 
 
 
 c3 
 Female vs Male 
 12.4642 
 0.2082 
 FALSE 
 
 
 c4 
 Female WT/WT vs Female Δ HEL/WT 
 -10.5804 
 0.5862 
 FALSE 
 
 
 c5 
 Male WT/WT vs Male Δ HEL/WT 
 -3.8911 
 0.9759 
 FALSE 
 
 
 c6 
 Female Δ HEL/WT vs Male Δ HEL/WT 
 15.8089 
 0.3466 
 FALSE 
 
 
 
    
 
 
  3.20.59  Resting CD4- NKT
cells - % of live (Panel A) 
 
 
 
 
 
 
 
 
 
 
  
 test 
 Difference 
 p_value 
 Significance 
 
 
 
 
 c2 
 WT/WT vs Δ HEL/WT 
 -0.0408 
 0.7922 
 FALSE 
 
 
 c3 
 Female vs Male 
 0.0858 
 0.2561 
 FALSE 
 
 
 c4 
 Female WT/WT vs Female Δ HEL/WT 
 -0.0719 
 0.6127 
 FALSE 
 
 
 c5 
 Male WT/WT vs Male Δ HEL/WT 
 -0.0097 
 0.9988 
 FALSE 
 
 
 c6 
 Female Δ HEL/WT vs Male Δ HEL/WT 
 0.1169 
 0.3235 
 FALSE 
 
 
 
    
 
 
  3.20.60  Resting CD4+ NKT
c. - % of CD4+ NKT c. (Panel A) 
 
 
 
 
 
 
 
 
 
 
  
 test 
 Difference 
 p_value 
 Significance 
 
 
 
 
 c2 
 WT/WT vs Δ HEL/WT 
 -11.1394 
 0.0358 
 TRUE 
 
 
 c3 
 Female vs Male 
 2.3206 
 0.9380 
 FALSE 
 
 
 c4 
 Female WT/WT vs Female Δ HEL/WT 
 -10.5788 
 0.2048 
 FALSE 
 
 
 c5 
 Male WT/WT vs Male Δ HEL/WT 
 -11.7000 
 0.2220 
 FALSE 
 
 
 c6 
 Female Δ HEL/WT vs Male Δ HEL/WT 
 1.7600 
 0.9911 
 FALSE 
 
 
 
    
 
 
  3.20.61  Resting CD4+ NKT
cells - % of live (Panel A) 
 
 
 
 
 
 
 
 
 
 
  
 test 
 Difference 
 p_value 
 Significance 
 
 
 
 
 c2 
 WT/WT vs Δ HEL/WT 
 -0.0081 
 0.6488 
 FALSE 
 
 
 c3 
 Female vs Male 
 0.0162 
 0.1025 
 FALSE 
 
 
 c4 
 Female WT/WT vs Female Δ HEL/WT 
 0.0033 
 0.9838 
 FALSE 
 
 
 c5 
 Male WT/WT vs Male Δ HEL/WT 
 -0.0195 
 0.2500 
 FALSE 
 
 
 c6 
 Female Δ HEL/WT vs Male Δ HEL/WT 
 0.0048 
 0.9674 
 FALSE 
 
 
 
    
 
 
  3.20.62  Resting CD4+ T
help c. - % of CD4+ T help (Panel 
 
 
 
 
 
 
 
 
 
 
  
 test 
 Difference 
 p_value 
 Significance 
 
 
 
 
 c2 
 WT/WT vs Δ HEL/WT 
 -6.5498 
 0.0004 
 TRUE 
 
 
 c3 
 Female vs Male 
 -11.3385 
 0.0000 
 TRUE 
 
 
 c4 
 Female WT/WT vs Female Δ HEL/WT 
 -4.9817 
 0.1004 
 FALSE 
 
 
 c5 
 Male WT/WT vs Male Δ HEL/WT 
 -8.1179 
 0.0041 
 TRUE 
 
 
 c6 
 Female Δ HEL/WT vs Male Δ HEL/WT 
 -12.9066 
 0.0005 
 TRUE 
 
 
 
    
 
 
  3.20.63  Resting CD4+ T
helper cells - % of live (Panel A) 
 
 
 
 
 
 
 
 
 
 
  
 test 
 Difference 
 p_value 
 Significance 
 
 
 
 
 c2 
 WT/WT vs Δ HEL/WT 
 -3.1115 
 0.0392 
 TRUE 
 
 
 c3 
 Female vs Male 
 -2.1172 
 0.2568 
 FALSE 
 
 
 c4 
 Female WT/WT vs Female Δ HEL/WT 
 -2.2288 
 0.4570 
 FALSE 
 
 
 c5 
 Male WT/WT vs Male Δ HEL/WT 
 -3.9943 
 0.1003 
 FALSE 
 
 
 c6 
 Female Δ HEL/WT vs Male Δ HEL/WT 
 -3.0000 
 0.3040 
 FALSE 
 
 
 
    
 
 
  3.20.64  Resting CD8+ T
cells - % of CD8+ T cells (Panel A) 
 
 
 
 
 
 
 
 
 
 
  
 test 
 Difference 
 p_value 
 Significance 
 
 
 
 
 c2 
 WT/WT vs Δ HEL/WT 
 3.3695 
 0.5316 
 FALSE 
 
 
 c3 
 Female vs Male 
 0.5480 
 0.9961 
 FALSE 
 
 
 c4 
 Female WT/WT vs Female Δ HEL/WT 
 3.2732 
 0.7524 
 FALSE 
 
 
 c5 
 Male WT/WT vs Male Δ HEL/WT 
 3.4657 
 0.7879 
 FALSE 
 
 
 c6 
 Female Δ HEL/WT vs Male Δ HEL/WT 
 0.6443 
 0.9981 
 FALSE 
 
 
 
    
 
 
  3.20.65  Resting CD8+ T
cells - %of live (Panel A) 
 
 
 
 
 
 
 
 
 
 
  
 test 
 Difference 
 p_value 
 Significance 
 
 
 
 
 c2 
 WT/WT vs Δ HEL/WT 
 0.1514 
 0.7694 
 FALSE 
 
 
 c3 
 Female vs Male 
 -0.0072 
 1.0000 
 FALSE 
 
 
 c4 
 Female WT/WT vs Female Δ HEL/WT 
 0.0634 
 0.9900 
 FALSE 
 
 
 c5 
 Male WT/WT vs Male Δ HEL/WT 
 0.2394 
 0.7391 
 FALSE 
 
 
 c6 
 Female Δ HEL/WT vs Male Δ HEL/WT 
 0.0809 
 0.9858 
 FALSE 
 
 
 
    
 
 
  3.20.66  Resting NK cells
- % of live leukocytes (Panel A) 
 
 
 
 
 
 
 
 
 
 
  
 test 
 Difference 
 p_value 
 Significance 
 
 
 
 
 c2 
 WT/WT vs Δ HEL/WT 
 -1.4129 
 0.2235 
 FALSE 
 
 
 c3 
 Female vs Male 
 1.3755 
 0.5174 
 FALSE 
 
 
 c4 
 Female WT/WT vs Female Δ HEL/WT 
 -1.0233 
 0.7178 
 FALSE 
 
 
 c5 
 Male WT/WT vs Male Δ HEL/WT 
 -1.8025 
 0.3406 
 FALSE 
 
 
 c6 
 Female Δ HEL/WT vs Male Δ HEL/WT 
 0.9859 
 0.8848 
 FALSE 
 
 
 
    
 
 
  3.20.67  Resting NK cells
- % of NK cells (Panel A) 
 
 
 
 
 
 
 
 
 
 
  
 test 
 Difference 
 p_value 
 Significance 
 
 
 
 
 c2 
 WT/WT vs Δ HEL/WT 
 -8.8541 
 0.3036 
 FALSE 
 
 
 c3 
 Female vs Male 
 -1.6684 
 0.9872 
 FALSE 
 
 
 c4 
 Female WT/WT vs Female Δ HEL/WT 
 -6.8339 
 0.7346 
 FALSE 
 
 
 c5 
 Male WT/WT vs Male Δ HEL/WT 
 -10.8743 
 0.4788 
 FALSE 
 
 
 c6 
 Female Δ HEL/WT vs Male Δ HEL/WT 
 -3.6886 
 0.9609 
 FALSE 
 
 
 
    
 
 
  3.20.68  Resting Treg
cells - % of live (Panel A) 
 
 
 
 
 
 
 
 
 
 
  
 test 
 Difference 
 p_value 
 Significance 
 
 
 
 
 c2 
 WT/WT vs Δ HEL/WT 
 -0.0512 
 0.9261 
 FALSE 
 
 
 c3 
 Female vs Male 
 -0.1483 
 0.2914 
 FALSE 
 
 
 c4 
 Female WT/WT vs Female Δ HEL/WT 
 -0.2089 
 0.2405 
 FALSE 
 
 
 c5 
 Male WT/WT vs Male Δ HEL/WT 
 0.1066 
 0.8266 
 FALSE 
 
 
 c6 
 Female Δ HEL/WT vs Male Δ HEL/WT 
 0.0094 
 0.9998 
 FALSE 
 
 
 
    
 
 
  3.20.69  Resting Treg
cells - % of Treg cells (Panel A) 
 
 
 
 
 
 
 
 
 
 
  
 test 
 Difference 
 p_value 
 Significance 
 
 
 
 
 c2 
 WT/WT vs Δ HEL/WT 
 -5.5197 
 0.0108 
 TRUE 
 
 
 c3 
 Female vs Male 
 -7.4452 
 0.0266 
 TRUE 
 
 
 c4 
 Female WT/WT vs Female Δ HEL/WT 
 -5.1598 
 0.1269 
 FALSE 
 
 
 c5 
 Male WT/WT vs Male Δ HEL/WT 
 -5.8796 
 0.1023 
 FALSE 
 
 
 c6 
 Female Δ HEL/WT vs Male Δ HEL/WT 
 -7.8052 
 0.1181 
 FALSE 
 
 
 
    
 
 
  3.20.70  T cells (Panel A)
- % of live leukocytes (Panel A) 
 
 
 
 
 
 
 
 
 
 
  
 test 
 Difference 
 p_value 
 Significance 
 
 
 
 
 c2 
 WT/WT vs Δ HEL/WT 
 -3.1367 
 0.1109 
 FALSE 
 
 
 c3 
 Female vs Male 
 -1.4087 
 0.8121 
 FALSE 
 
 
 c4 
 Female WT/WT vs Female Δ HEL/WT 
 -2.9003 
 0.3884 
 FALSE 
 
 
 c5 
 Male WT/WT vs Male Δ HEL/WT 
 -3.3731 
 0.3493 
 FALSE 
 
 
 c6 
 Female Δ HEL/WT vs Male Δ HEL/WT 
 -1.6450 
 0.8850 
 FALSE 
 
 
 
    
 
 
  3.20.71  T cells (panel B)
- % of live leukocytes (Panel B) 
 
 
 
 
 
 
 
 
 
 
  
 test 
 Difference 
 p_value 
 Significance 
 
 
 
 
 c2 
 WT/WT vs Δ HEL/WT 
 -3.5600 
 0.1312 
 FALSE 
 
 
 c3 
 Female vs Male 
 -1.4606 
 0.8614 
 FALSE 
 
 
 c4 
 Female WT/WT vs Female Δ HEL/WT 
 -3.9583 
 0.2715 
 FALSE 
 
 
 c5 
 Male WT/WT vs Male Δ HEL/WT 
 -3.1617 
 0.5401 
 FALSE 
 
 
 c6 
 Female Δ HEL/WT vs Male Δ HEL/WT 
 -1.0623 
 0.9769 
 FALSE 
 
 
 
    
 
 
  3.20.72  Treg cells - % of
CD4 T cells (Panel A) 
 
 
 
 
 
 
 
 
 
 
  
 test 
 Difference 
 p_value 
 Significance 
 
 
 
 
 c2 
 WT/WT vs Δ HEL/WT 
 0.9618 
 0.1108 
 FALSE 
 
 
 c3 
 Female vs Male 
 -0.5411 
 0.5720 
 FALSE 
 
 
 c4 
 Female WT/WT vs Female Δ HEL/WT 
 -0.2455 
 0.9706 
 FALSE 
 
 
 c5 
 Male WT/WT vs Male Δ HEL/WT 
 2.1691 
 0.0046 
 TRUE 
 
 
 c6 
 Female Δ HEL/WT vs Male Δ HEL/WT 
 0.6663 
 0.7137 
 FALSE 
 
 
 
    
 
 
  3.20.73  Treg cells - % of
live leukocytes (Panel A) 
 
 
 
 
 
 
 
 
 
 
  
 test 
 Difference 
 p_value 
 Significance 
 
 
 
 
 c2 
 WT/WT vs Δ HEL/WT 
 0.0475 
 0.9779 
 FALSE 
 
 
 c3 
 Female vs Male 
 -0.1033 
 0.8491 
 FALSE 
 
 
 c4 
 Female WT/WT vs Female Δ HEL/WT 
 -0.1728 
 0.6920 
 FALSE 
 
 
 c5 
 Male WT/WT vs Male Δ HEL/WT 
 0.2678 
 0.4356 
 FALSE 
 
 
 c6 
 Female Δ HEL/WT vs Male Δ HEL/WT 
 0.1169 
 0.9221 
 FALSE 
 
 
 
     
 
 
 
  3.21  Gross Pathology 
  Number of animals  
    
 
 
 
 STRAIN 
 GENDER 
 count 
 
 
 
 
 WT/WT 
 female 
 8 
 
 
 WT/WT 
 male 
 8 
 
 
 Δ HEL/WT 
 female 
 7 
 
 
 Δ HEL/WT 
 male 
 6 
 
 
 
   
 
  3.21.1  Adrenal gland 
    
    
 
 
  3.21.2  Bone 
    
    
 
 
  3.21.3  Bone marrow 
    
    
 
 
  3.21.4  Brain 
    
    
 
 
  3.21.5  Epididymis 
    
    
 
 
  3.21.6  Esophagus 
    
    
 
 
  3.21.7  Eye with optic
nerve 
    
    
 
 
  3.21.8  Femur 
    
    
 
 
  3.21.9  Gall bladder 
    
    
 
 
  3.21.10  Heart 
    
    
 
 
  3.21.11  Kidney 
    
    
 
 
  3.21.12  Large
intestine 
    
    
 
 
  3.21.13  Liver 
    
    
 
 
  3.21.14  Lung 
    
    
 
 
  3.21.15  Lymph node 
    
    
 
 
  3.21.16  Mammary
gland 
    
    
 
 
  3.21.17  Ovary 
    
    
 
 
  3.21.18  Pancreas 
    
    
 
 
  3.21.19  Prostate 
    
    
 
 
  3.21.20  Salivary
gland 
    
    
 
 
  3.21.21  Seminal
vesicle 
    
    
 
 
  3.21.22  Skeletal
muscle 
    
    
 
 
  3.21.23  Skin 
    
    
 
 
  3.21.24  Small
intestine 
    
    
 
 
  3.21.25  Spinal cord 
    
    
 
 
  3.21.26  Spleen 
    
    
 
 
  3.21.27  Stomach 
    
    
 
 
  3.21.28  Testes 
    
    
 
 
  3.21.29  Thymus 
   
 
 
 
  
 female WT/WT 
 female Δ HEL/WT 
 male WT/WT 
 
 
 
 
 female Δ HEL/WT 
 0.300 
 NA 
 NA 
 
 
 male WT/WT 
 1.000 
 0.6831 
 NA 
 
 
 male Δ HEL/WT 
 0.018 
 0.2051 
 0.0769 
 
 
 
   
   
    
 
 
  3.21.30  Thyroid 
    
    
 
 
  3.21.31  Tooth 
    
    
 
 
  3.21.32  Trachea 
    
    
 
 
  3.21.33  Urinary
bladder 
    
    
 
 
  3.21.34  Uterus 
    
    
 
 
 
 
 
 
 
 
 
 
 
 SPECIMEN_ID 
 GENE 
 GENDER 
 DATE 
 Tissue 
 Ontology 
 
 
 
 
 28Y-25531 
 deltaHEL1 
 male 
 2017-07-16 
 Thymus 
 MP:0000703:abnormal thymus morphology 
 
 
 28Y-25532 
 deltaHEL1 
 male 
 2017-07-16 
 Thymus 
 MP:0000703:abnormal thymus morphology 
 
 
 28Y-25576 
 deltaHEL1 
 male 
 2017-07-16 
 Thymus 
 MP:0000703:abnormal thymus morphology 
 
 
 28Y-25577 
 deltaHEL1 
 male 
 2017-07-16 
 Thymus 
 MP:0000703:abnormal thymus morphology 
 
 
 28Y-25579 
 deltaHEL1 
 male 
 2017-07-16 
 Thymus 
 MP:0000703:abnormal thymus morphology 
 
 
 28Y-25589 
 deltaHEL1 
 female 
 2017-07-17 
 Thymus 
 MP:0000703:abnormal thymus morphology 
 
 
 28Y-25592 
 deltaHEL1 
 female 
 2018-07-18 
 Thymus 
 MP:0000703:abnormal thymus morphology 
 
 
 
 
 
 


 
 

 

 

 

 

 

 

 
 

 
 
