# Supplementary material for: Functional canonical RNAi in mice expressing a truncated Dicer isoform and long dsRNA
Source: EMBO Rep. 2024 May 20;25(7):9. doi: 10.1038/s44319-024-00148-z (PMC11239679; doi:10.1038/s44319-024-00148-z)
Supplement: Supplementary file 9 — Expanded View Figures [file 44319_2024_148_MOESM9_ESM.pdf]

Expanded View Figures

A

	M x F	wt/wt	ΔHEL1/wt	ΔHEL1/ΔHEL1	M	F	litters	litter size
B6	*ΔHEL1/wt x wt/wt*	44%	56%	-	43%	57%	19	7.7±2.7
	wt/wt x ΔHEL1/wt	53%	47%	-	55%	45%	36	5.9±2.4
	ΔHEL1 /wt x ΔHEL1/wt	36%	64%	0%	54%	46%	22	5.3±2.3
ICR	*ΔHEL1/wt x wt/wt*	49%	51%	-	48%	52%	10	12.3±3.0
	wt/wt x ΔHEL1/wt	52%	48%	-	40%	60%	9	9.3±4.6
	ΔHEL1 /wt x ΔHEL1/wt	35%	65%	0%	49%	51%	25	7.4±2.8

B

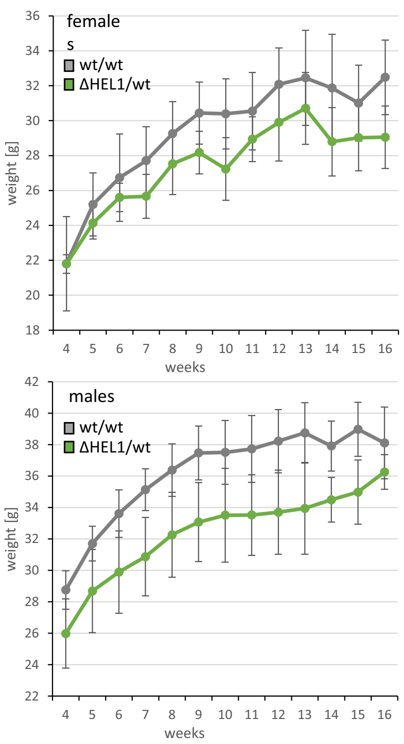

C

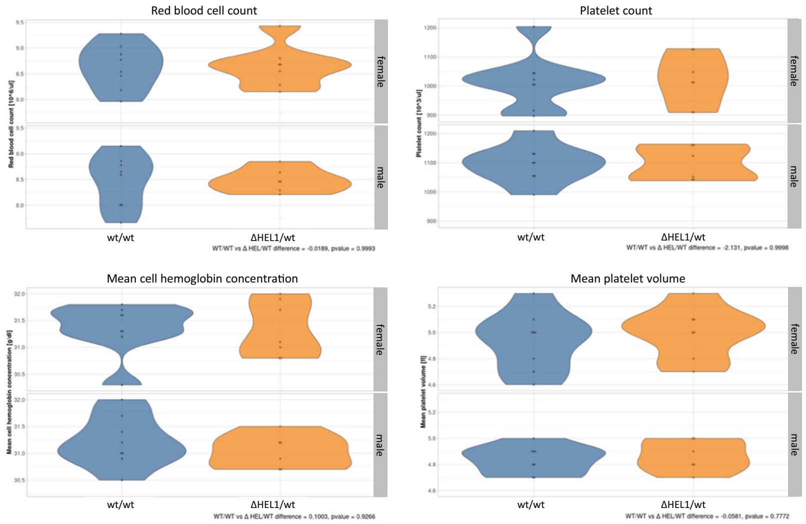

D

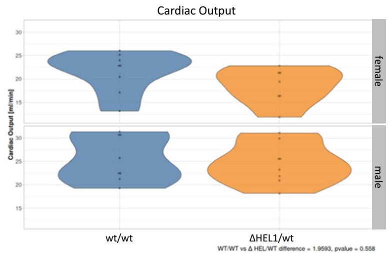

Figure EV1. Selected phenotype features.

(**A**) Breeding performance of heterozygous mutants expressed as percentages. (**B**) Growth curves of males and females on the ICR background. 8 animals were analyzed for each genotype. Data are presented as mean +/− SD error bars. (**C**) *Dicer*$^{\Delta HEL1/wt}$ animals have normal cardiac output. (**D**) Blood parameters disrupted in *Dicer*$^{\Delta HEL1/\Delta HEL1}$ (Zapletal et al, 2022) are normal in *Dicer*$^{\Delta HEL1/wt}$ mice. For details on the methodology, see the full phenotyping report in the Source Data for EV1. Source data are available online for this figure.

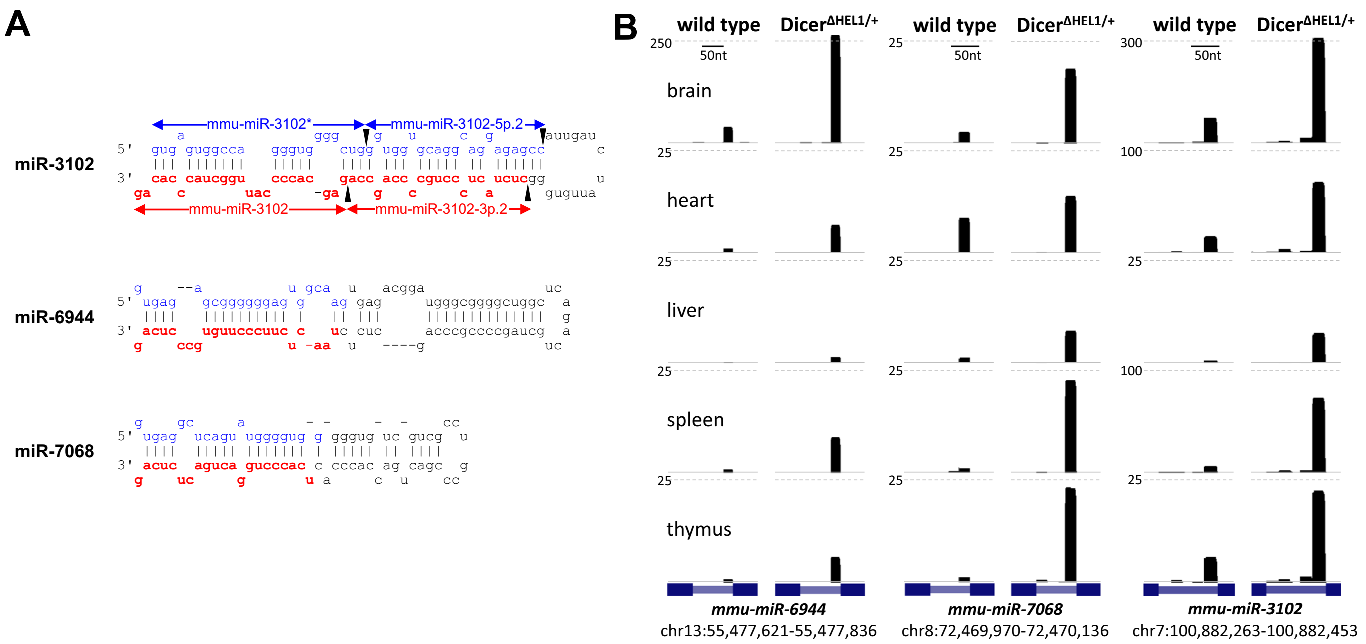

Figure EV2. Supplementary data for miRNome changes.

(**A**) Most upregulated mirtrons. Mirtron precursor schemes were adapted from structures presented in miRBase (Kozomara et al, 2019). (**B**) UCSC browser snapshots showing abundance of 21–23 nt reads in mirtron loci in normal and ΔHEL1 mice. The vertical scale is in counts per million (CPM) of 18–32 nt reads.

© The Author(s)

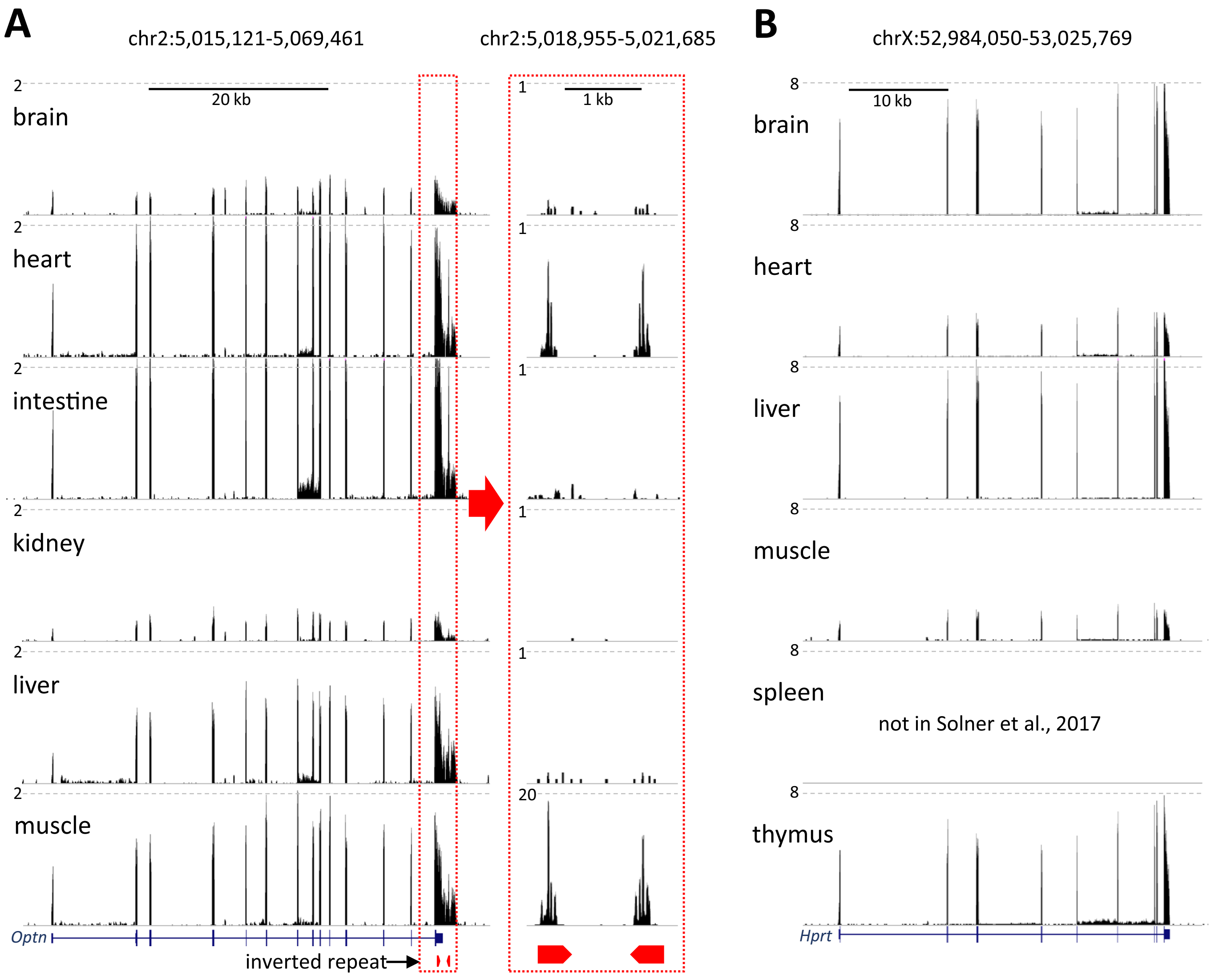

Figure EV3. Supplementary data for siRNA analysis.

(**A**) *Optn* expression and siRNA production in different organs in wild-type mice. On the left side is a UCSC browser snapshot of *Optn* transcript expression in different organs using publicly available RNA-seq libraries from different organs (Sollner et al, 2017). The vertical scale is counts per million of reads (CPM). Next to it are 21–23 nt RNAs from small RNA sequencing data from the same organs (Isakova et al, 2020) mapped into the *Optn* inverted repeat region (red pentagons). The vertical scale is in counts per million of 19–32 nt reads. (**B**) UCSC browser snapshot of *Hprt* expression in different organs in wild-type mice using publicly available RNA-seq libraries from different organs (Sollner et al, 2017).

© The Author(s)

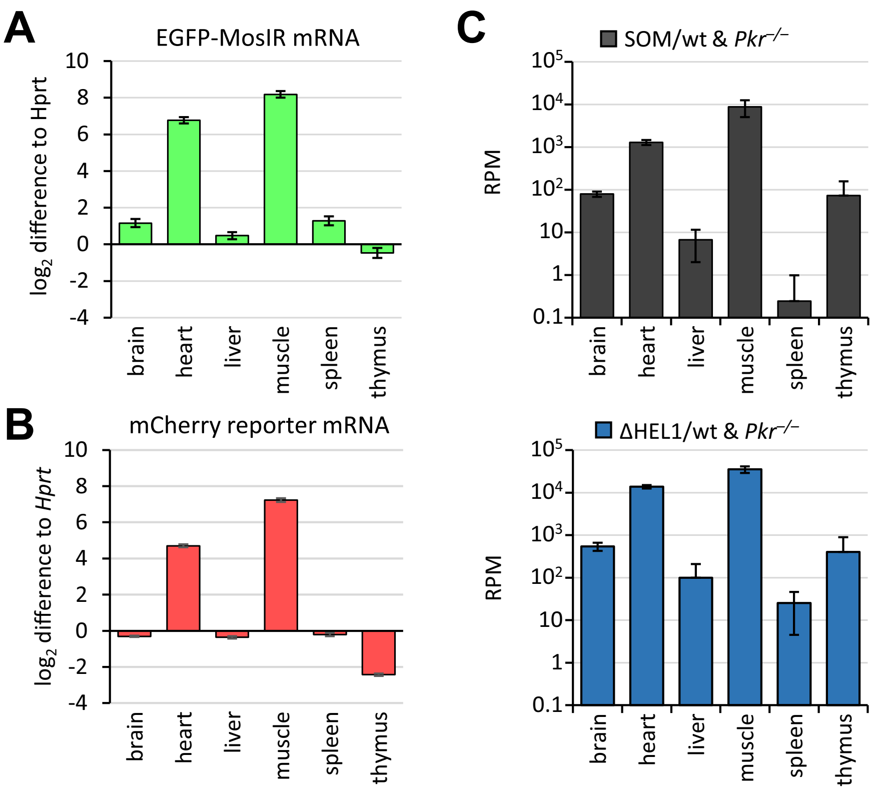

Figure EV4. Supplementary data for RNAi analysis—logscale presentation of transcript and siRNA abundance.

(**A**) qPCR analysis of CAG-EGFP-MosIR transgene expression in *Dicer*$^{SOM/wt}$ *Pkr*$^{-/-}$ organs from Fig. 5C presented as $\log_2$ relative difference to *Hprt* expression. Data come from three (muscle and spleen) or five (other organs) biological replicates analyzed in technical triplicates. (**B**) qPCR analysis of CAG-mCherry-Mos transgene expression in *Dicer*$^{SOM/wt}$ *Pkr*$^{-/-}$ organs of a single animal from Fig. 6B presented as $\log_2$ relative difference to *Hprt* expression. qPCR analysis was done in technical triplicates. (**C**) MosIR endo-siRNA abundance—shown is 21–23 nt RNA abundance in reads per million (RPM) of all 18–32 nt mapped small RNA reads. All bar graphs depict mean +/− SD error bars. Source data are available online for this figure.

 © The Author(s)
